# Supplementary material for: Early and Late Buzzards: Comparing Different Approaches for Quantile‐Based Multiple Testing in Heavy‐Tailed Wildlife Research Data
Source: Biom J. 2025 Jul 4;67(4):e70065. doi: 10.1002/bimj.70065 (PMC12227168; doi:10.1002/bimj.70065)
Supplement: Supplementary file 1 — Supporting File 1: bimj70065‐sup‐0001‐SuppMat.pdf. [file BIMJ-67-e70065-s001.pdf]

Supplement of: Early and Late Buzzards: Comparing Different Approaches for Quantile-based Multiple Testing in Heavy-Tailed Wildlife Research Data

Marléne Baumeister[*,1,2], Merle Munko[*,3], Kai-Philipp Gladow[4], Marc Ditzhaus[3], Nayden Chakarov[4,5], Markus Pauly[1,2]

April 23, 2025

Abstract

In this project, we compare different inference approaches for two-sided and non-inferiority hypotheses formulated in terms of medians or IQRs in an extensive simulation study. We consider multiple contrast testing procedures combined with a bootstrap method as well as testing procedures with Bonferroni correction. As an example of a multiple testing problem based on heavy-tailed data we analyse an ecological trait variation in early and late breeding in a medium-sized bird of prey. This Supplement contains the detailed simulation results. Further plots and tables on the simulation results of the paper are provided.

This document is licensed under Creative Commons Attribution 4.0 International (CC-BY 4.0).

[*]These authors contributed equally to this work.
[1]Department of Statistics, TU Dortmund University, Germany
[2]Research Center Trustworthy Data Science and Security, UA Ruhr, Germany
[3]Department of Mathematics, Otto-von-Guericke University Magdeburg, Germany
[4]Department of Animal Behaviour, Bielefeld University, Germany
[5]Joint Institute for Individualisation in a Changing Environment (JICE), Bielefeld University and University of Münster, Germany

Contents

1 Plot on the Impact of (Un)Balanced Designs 3

2 Plots and Tables for Empirical Global Power 4

3 Plots for Empirical Local Power 80

4 Additional Simulation Study 85

1 Plot on the Impact of (Un)Balanced Designs

To investigate the impact of balanced and unbalanced designs, Figure 1 shows the rejection rates under the null hypothesis exemplarily for the non-inferiority Dunnett-type tests. This setting is chosen for illustration due to the real data example in Section 5 of the paper. It is observable that the empirical FWERs for balanced and unbalanced designs are comparable for all methods and variance estimators.

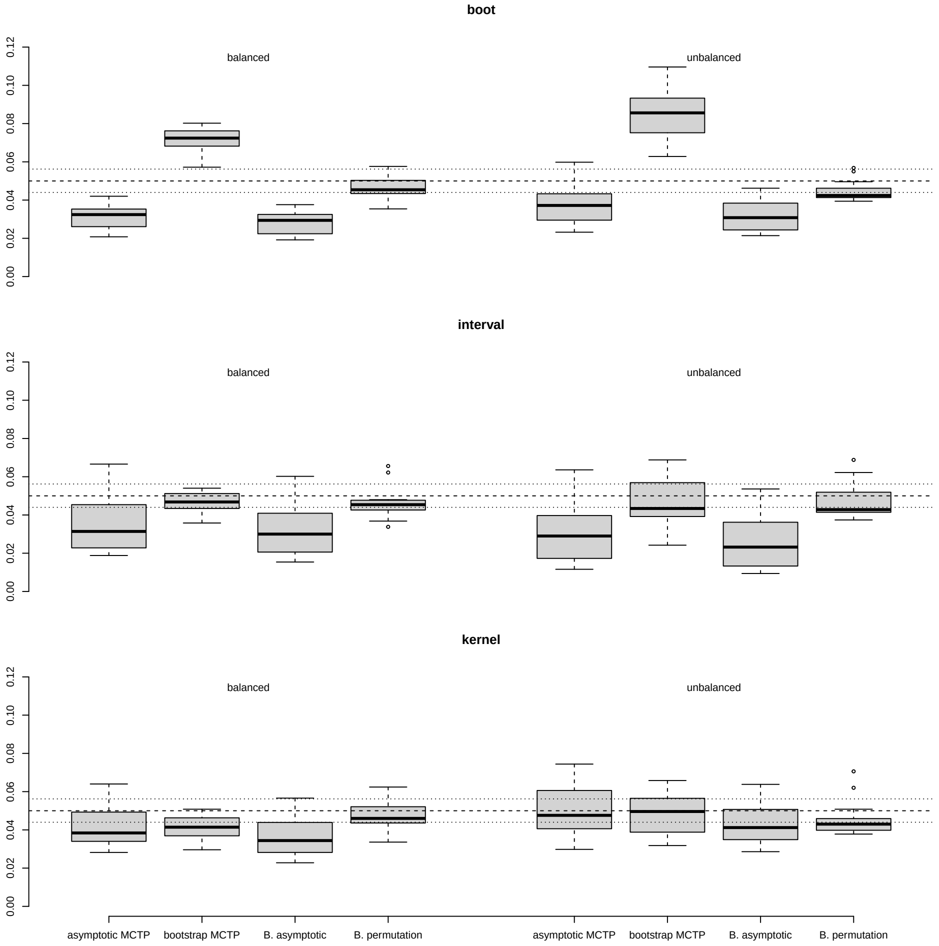

Figure 1: Empirical FWERs for non-inferiority Dunnett-type tests with different designs (left: balanced and right: unbalanced) and variance estimators (from left to right: bootstrap, interval-based or kernel).

2 Plots and Tables for Empirical Global Power

Plots for the analyzing the global power are shown in Figures 2–10. Here and throughout, the following abbreviations are used: boot, interval, kernel - bootstrap, interval-based, and kernel variance estimator, respectively. The results of all different scenarios can be found in Tables 1–72. The following abbreviations are used in the tables: bal and unb for balanced ($\mathbf{n}_1$) and unbalanced ($\mathbf{n}_2$) designs, hom, pos and neg for homoscedastic scenario ($\boldsymbol{\sigma}_1$), positive ($\boldsymbol{\sigma}_2$) and negative ($\boldsymbol{\sigma}_3$) pairing, asymp., boot. and perm. for asymptotic, bootstrap and permutation, respectively, and B. for Bonferroni-adjusted.

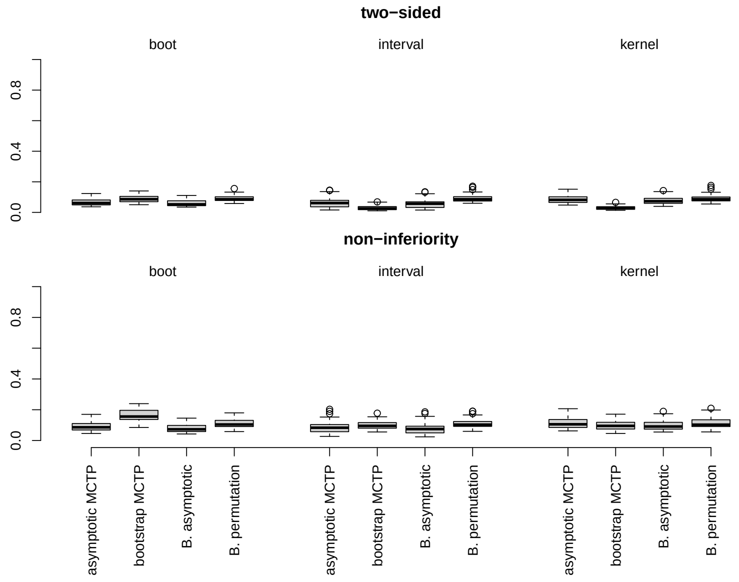

Figure 2: Empirical global power with $\delta = 0.5$ for Dunnett-type contrasts with different hypotheses (top: two-sided, bottom: non-inferiority) and variance estimators.

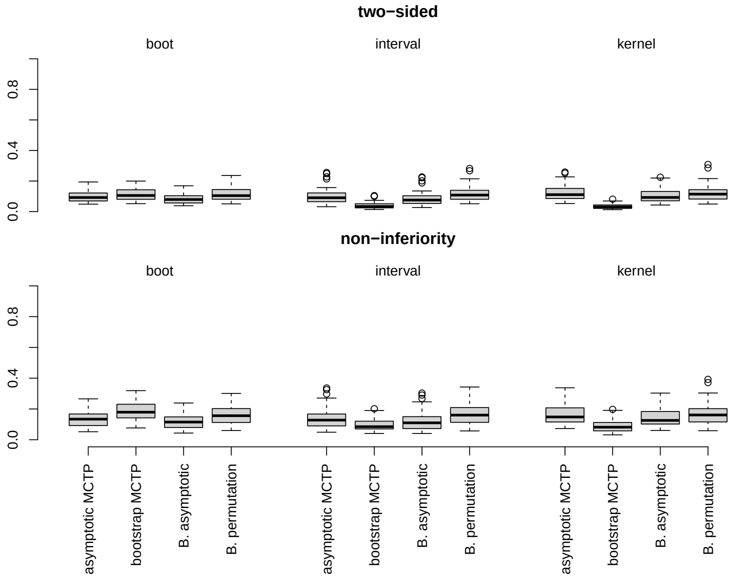

Figure 3: Empirical global power with $\delta = 0.5$ for Tukey-type contrasts with different hypotheses (top: two-sided, bottom: non-inferiority) and variance estimators.

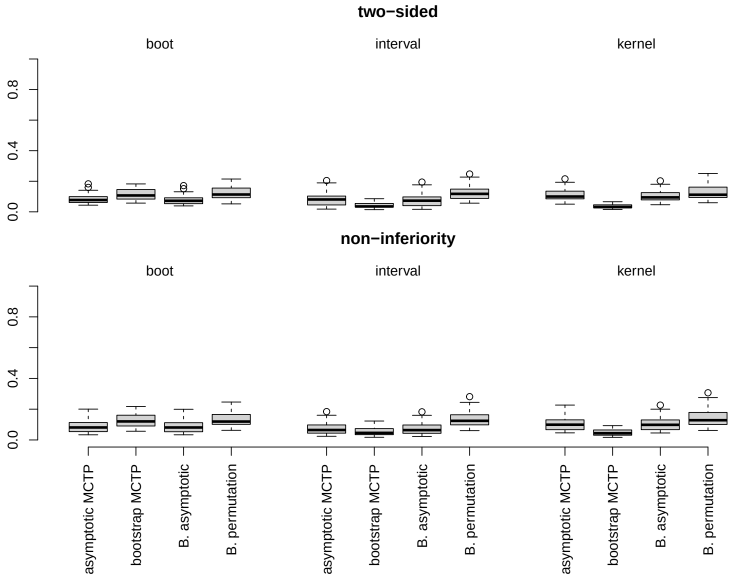

Figure 4: Empirical global power with $\delta = 0.5$ for Grand-mean-type contrasts with different hypotheses (top: two-sided, bottom: non-inferiority) and variance estimators.

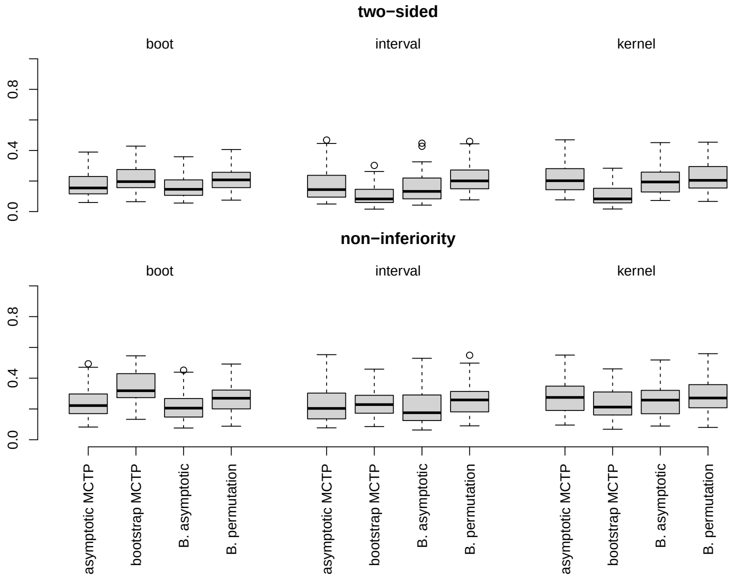

Figure 5: Empirical global power with $\delta = 1.0$ for Dunnett-type contrasts with different hypotheses (top: two-sided, bottom: non-inferiority) and variance estimators.

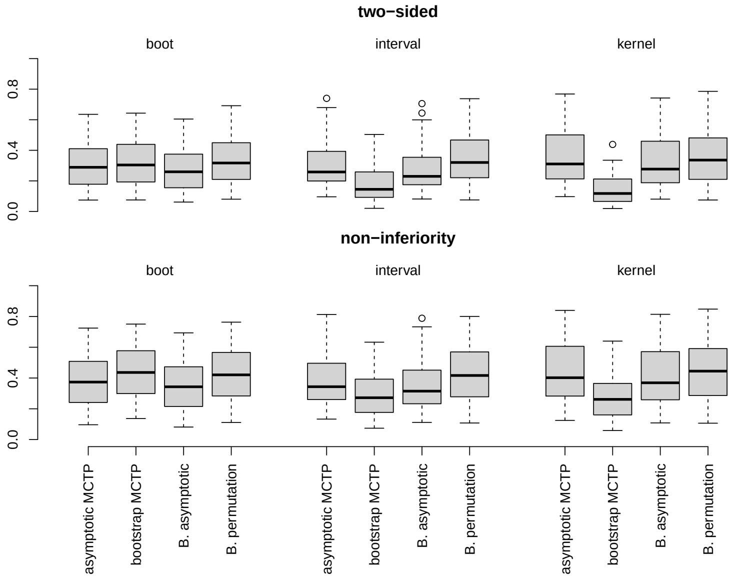

Figure 6: Empirical global power with $\delta = 1.0$ for Tukey-type contrasts with different hypotheses (top: two-sided, bottom: non-inferiority) and variance estimators.

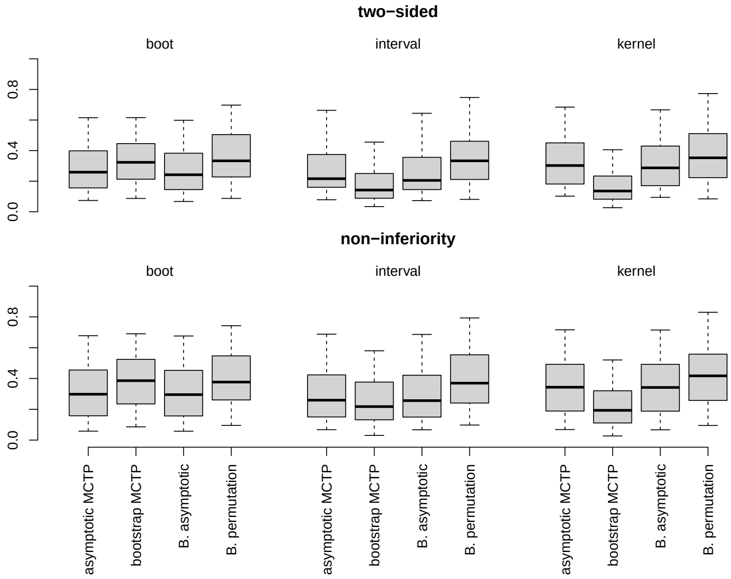

Figure 7: Empirical global power with $\delta = 1.0$ for Grand-mean-type contrasts with different hypotheses (top: two-sided, bottom: non-inferiority) and variance estimators.

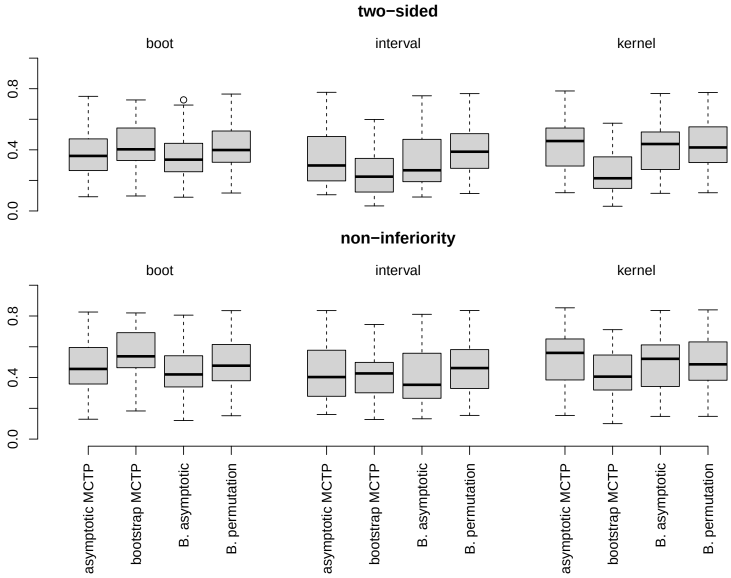

Figure 8: Empirical global power with $\delta = 1.5$ for Dunnett-type contrasts with different hypotheses (top: two-sided, bottom: non-inferiority) and variance estimators.

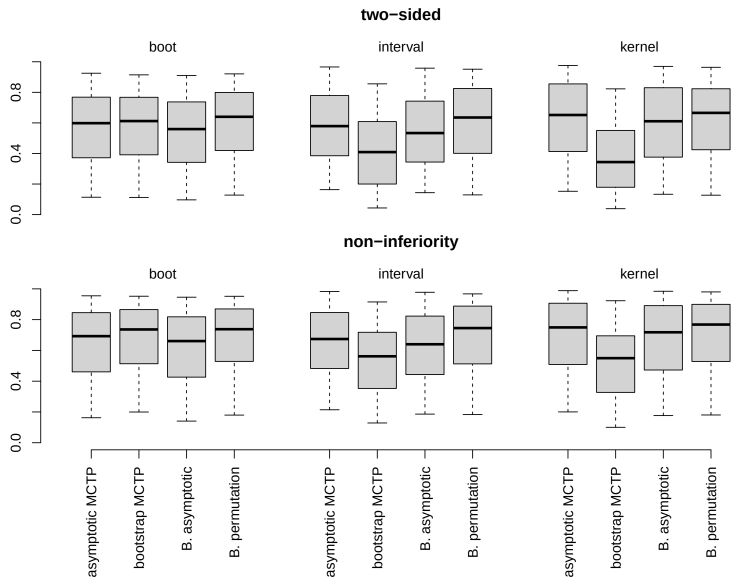

Figure 9: Empirical global power with $\delta = 1.5$ for Tukey-type contrasts with different hypotheses (top: two-sided, bottom: non-inferiority) and variance estimators.

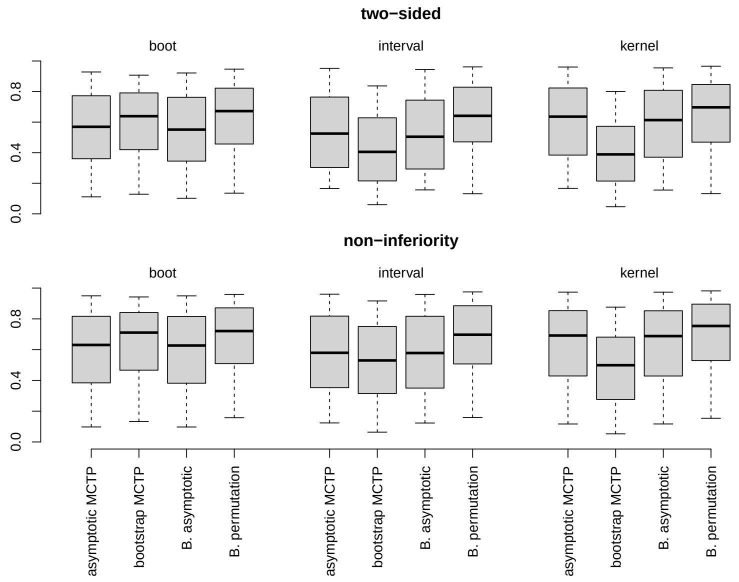

Figure 10: Empirical global power with $\delta = 1.5$ for Grand-mean-type contrasts with different hypotheses (top: two-sided, bottom: non-inferiority) and variance estimators.

Setting				Method			
Distribution	Design		δ	asymp. MCTP	boot. MCTP	B. asymp.	B. perm.
χ^2_3	bal	hom	0.0	3.66	**4.74**	3.32	**4.68**
χ^2_3	bal	neg	0.0	4.30	5.72	3.68	**5.40**
χ^2_3	bal	pos	0.0	**4.60**	**5.02**	4.40	6.32
χ^2_3	unb	hom	0.0	3.42	**4.66**	3.08	3.80
χ^2_3	unb	neg	0.0	**4.44**	5.64	3.76	**5.00**
χ^2_3	unb	pos	0.0	4.02	4.32	3.74	**4.66**
$\mathcal{LN}(0,1)$	bal	hom	0.0	2.28	3.50	1.94	4.30
$\mathcal{LN}(0,1)$	bal	neg	0.0	2.42	4.20	1.96	**4.58**
$\mathcal{LN}(0,1)$	bal	pos	0.0	2.62	3.82	2.42	**5.34**
$\mathcal{LN}(0,1)$	unb	hom	0.0	2.22	3.46	1.96	4.04
$\mathcal{LN}(0,1)$	unb	neg	0.0	3.22	**5.02**	2.96	**5.54**
$\mathcal{LN}(0,1)$	unb	pos	0.0	2.48	3.70	2.22	**4.72**
$\mathcal{N}(0,1)$	bal	hom	0.0	3.40	4.36	3.12	3.62
$\mathcal{N}(0,1)$	bal	neg	0.0	**4.54**	5.66	3.92	**4.60**
$\mathcal{N}(0,1)$	bal	pos	0.0	**4.60**	**4.90**	4.36	**5.40**
$\mathcal{N}(0,1)$	unb	hom	0.0	4.06	**5.00**	3.42	3.90
$\mathcal{N}(0,1)$	unb	neg	0.0	**4.94**	5.94	4.08	**4.80**
$\mathcal{N}(0,1)$	unb	pos	0.0	4.28	**5.00**	4.02	**4.96**
t_2	bal	hom	0.0	2.74	**4.88**	2.40	4.36
t_2	bal	neg	0.0	2.96	**5.24**	2.56	**4.48**
t_2	bal	pos	0.0	2.56	4.26	2.34	5.66
t_2	unb	hom	0.0	2.16	4.04	1.82	3.88
t_2	unb	neg	0.0	2.42	4.16	1.94	4.18
t_2	unb	pos	0.0	2.32	3.98	2.24	**5.24**
t3	bal	hom	0.0	3.36	**5.28**	3.06	**4.68**
t3	bal	neg	0.0	3.20	**5.42**	2.58	**4.52**
t3	bal	pos	0.0	3.76	**5.12**	3.54	5.92
t3	unb	hom	0.0	2.84	**4.52**	2.52	4.24
t3	unb	neg	0.0	3.12	**5.12**	2.60	**4.82**
t3	unb	pos	0.0	2.78	**4.56**	2.54	**5.02**

Table 1: Empirical FWER in % of two-sided Dunnett-type tests with bootstrap estimator

Setting				Method			
Distribution	Design		δ	asymp. MCTP	boot. MCTP	B. asymp.	B. perm.
χ^2_3	bal	hom	0.0	3.52	6.80	3.28	**4.52**
χ^2_3	bal	neg	0.0	4.20	7.74	3.76	**5.24**
χ^2_3	bal	pos	0.0	3.52	6.48	3.32	**5.12**
χ^2_3	unb	hom	0.0	4.18	8.78	3.46	4.22
χ^2_3	unb	neg	0.0	**5.52**	10.26	**4.62**	**5.50**
χ^2_3	unb	pos	0.0	3.72	6.84	3.48	4.28
$\mathcal{LN}(0,1)$	bal	hom	0.0	2.46	5.82	2.22	4.18
$\mathcal{LN}(0,1)$	bal	neg	0.0	3.24	7.50	2.94	5.76
$\mathcal{LN}(0,1)$	bal	pos	0.0	2.08	5.72	1.92	**4.54**
$\mathcal{LN}(0,1)$	unb	hom	0.0	2.68	6.78	2.46	4.12
$\mathcal{LN}(0,1)$	unb	neg	0.0	**4.44**	8.56	3.92	5.68
$\mathcal{LN}(0,1)$	unb	pos	0.0	2.32	6.28	2.20	3.94
$\mathcal{N}(0,1)$	bal	hom	0.0	3.24	6.88	2.60	3.54
$\mathcal{N}(0,1)$	bal	neg	0.0	3.88	7.46	3.22	4.08
$\mathcal{N}(0,1)$	bal	pos	0.0	3.88	7.02	3.60	**4.94**
$\mathcal{N}(0,1)$	unb	hom	0.0	**4.66**	9.20	3.92	4.14
$\mathcal{N}(0,1)$	unb	neg	0.0	5.98	10.96	**4.56**	**4.96**
$\mathcal{N}(0,1)$	unb	pos	0.0	3.96	7.46	3.76	4.10
t_2	bal	hom	0.0	2.54	7.48	2.26	**4.52**
t_2	bal	neg	0.0	2.68	8.02	2.08	4.20
t_2	bal	pos	0.0	2.46	6.84	2.18	**4.86**
t_2	unb	hom	0.0	2.68	8.36	2.14	4.02
t_2	unb	neg	0.0	3.22	9.46	2.42	4.18
t_2	unb	pos	0.0	2.52	7.58	2.30	**4.46**
t_3	bal	hom	0.0	3.54	7.94	3.04	**4.78**
t_3	bal	neg	0.0	3.42	8.00	2.62	**4.48**
t_3	bal	pos	0.0	3.18	7.24	3.00	**5.18**
t_3	unb	hom	0.0	3.58	8.92	2.88	4.24
t_3	unb	neg	0.0	4.22	10.26	2.92	**4.74**
t_3	unb	pos	0.0	3.46	7.96	3.08	**4.50**

Table 2: Empirical FWER in % of non-inferiority Dunnett-type tests with bootstrap estimator

Setting				Method			
Distribution	Design		δ	asymp. MCTP	boot. MCTP	B. asymp.	B. perm.
χ^2_3	bal	hom	0.0	**5.00**	1.54	**4.54**	4.32
χ^2_3	bal	neg	0.0	6.68	2.16	5.64	**5.56**
χ^2_3	bal	pos	0.0	6.34	1.68	6.02	6.22
χ^2_3	unb	hom	0.0	4.04	1.18	3.54	3.98
χ^2_3	unb	neg	0.0	**4.44**	1.64	3.90	**5.40**
χ^2_3	unb	pos	0.0	4.36	1.08	3.90	5.64
$\mathcal{LN}(0,1)$	bal	hom	0.0	**5.50**	0.80	**5.10**	**4.50**
$\mathcal{LN}(0,1)$	bal	neg	0.0	**5.42**	1.38	**4.74**	**5.22**
$\mathcal{LN}(0,1)$	bal	pos	0.0	6.20	1.22	5.66	6.20
$\mathcal{LN}(0,1)$	unb	hom	0.0	3.64	0.80	3.26	4.18
$\mathcal{LN}(0,1)$	unb	neg	0.0	**4.74**	2.12	4.10	6.18
$\mathcal{LN}(0,1)$	unb	pos	0.0	3.94	0.78	3.62	5.64
$\mathcal{N}(0,1)$	bal	hom	0.0	3.10	1.36	2.68	3.76
$\mathcal{N}(0,1)$	bal	neg	0.0	3.56	2.10	3.04	**4.80**
$\mathcal{N}(0,1)$	bal	pos	0.0	4.00	1.80	3.78	5.88
$\mathcal{N}(0,1)$	unb	hom	0.0	2.18	0.98	1.78	4.04
$\mathcal{N}(0,1)$	unb	neg	0.0	2.48	1.28	1.96	**4.86**
$\mathcal{N}(0,1)$	unb	pos	0.0	2.38	0.92	2.12	**5.46**
t_2	bal	hom	0.0	1.90	1.28	1.76	**4.86**
t_2	bal	neg	0.0	2.30	1.72	1.98	**4.88**
t_2	bal	pos	0.0	1.88	1.16	1.70	**5.52**
t_2	unb	hom	0.0	0.98	0.58	0.90	4.22
t_2	unb	neg	0.0	0.94	0.48	0.82	4.12
t_2	unb	pos	0.0	1.00	0.54	0.96	5.70
t_3	bal	hom	0.0	2.38	1.60	2.18	**4.52**
t_3	bal	neg	0.0	2.36	1.46	2.04	**4.88**
t_3	bal	pos	0.0	3.14	1.56	2.92	5.82
t_3	unb	hom	0.0	1.20	0.68	1.00	**4.44**
t_3	unb	neg	0.0	1.60	0.74	1.38	**4.56**
t_3	unb	pos	0.0	1.56	0.62	1.40	5.64

Table 3: Empirical FWER in % of two-sided Dunnett-type tests with interval-based estimator

Setting				Method			
Distribution	Design		δ	asymp. MCTP	boot. MCTP	B. asymp.	B. perm.
χ_3^2	bal	hom	0.0	**4.88**	**4.42**	4.24	4.12
χ_3^2	bal	neg	0.0	6.66	**5.30**	5.86	6.22
χ_3^2	bal	pos	0.0	4.20	4.26	3.94	4.14
χ_3^2	unb	hom	0.0	**5.18**	6.22	**4.60**	4.28
χ_3^2	unb	neg	0.0	6.04	6.88	**5.04**	6.22
χ_3^2	unb	pos	0.0	3.90	4.34	3.58	3.96
$\mathcal{LN}(0,1)$	bal	hom	0.0	**5.22**	4.18	**4.70**	**4.76**
$\mathcal{LN}(0,1)$	bal	neg	0.0	6.60	**5.08**	6.02	6.56
$\mathcal{LN}(0,1)$	bal	pos	0.0	3.88	3.58	3.70	3.68
$\mathcal{LN}(0,1)$	unb	hom	0.0	4.04	**4.64**	3.66	4.12
$\mathcal{LN}(0,1)$	unb	neg	0.0	6.36	6.42	**5.36**	6.88
$\mathcal{LN}(0,1)$	unb	pos	0.0	3.36	3.90	3.24	4.34
$\mathcal{N}(0,1)$	bal	hom	0.0	2.58	**4.68**	2.04	3.38
$\mathcal{N}(0,1)$	bal	neg	0.0	3.90	**5.40**	3.08	**4.64**
$\mathcal{N}(0,1)$	bal	pos	0.0	3.14	**4.48**	3.00	4.38
$\mathcal{N}(0,1)$	unb	hom	0.0	2.90	**5.16**	2.32	3.74
$\mathcal{N}(0,1)$	unb	neg	0.0	3.64	6.60	2.90	**5.20**
$\mathcal{N}(0,1)$	unb	pos	0.0	2.56	4.04	2.06	**4.74**
t_2	bal	hom	0.0	2.16	**4.68**	1.76	**4.52**
t_2	bal	neg	0.0	1.88	**4.92**	1.54	**4.54**
t_2	bal	pos	0.0	1.88	4.16	1.76	**4.76**
t_2	unb	hom	0.0	1.24	3.42	0.94	4.08
t_2	unb	neg	0.0	1.38	3.96	1.02	4.18
t_2	unb	pos	0.0	1.16	2.42	1.02	**5.46**
t_3	bal	hom	0.0	2.38	**5.16**	2.10	**4.48**
t_3	bal	neg	0.0	2.64	**5.40**	2.16	**4.80**
t_3	bal	pos	0.0	2.18	**4.44**	2.08	**4.78**
t_3	unb	hom	0.0	1.92	3.94	1.36	4.16
t_3	unb	neg	0.0	2.02	4.34	1.46	4.24
t_3	unb	pos	0.0	1.54	3.36	1.30	**5.18**

Table 4: Empirical FWER in % of non-inferiority Dunnett-type tests with interval-based estimator

Setting				Method			
Distribution	Design		δ	asymp. MCTP	boot. MCTP	B. asymp.	B. perm.
χ^2_3	bal	hom	0.0	**5.08**	1.40	**4.78**	4.14
χ^2_3	bal	neg	0.0	6.58	1.90	5.70	5.84
χ^2_3	bal	pos	0.0	6.56	1.48	6.12	6.82
χ^2_3	unb	hom	0.0	**5.50**	1.42	**5.00**	4.14
χ^2_3	unb	neg	0.0	6.62	1.84	5.74	5.78
χ^2_3	unb	pos	0.0	**5.62**	1.26	**5.26**	**4.82**
$\mathcal{LN}(0,1)$	bal	hom	0.0	3.90	0.74	3.46	4.36
$\mathcal{LN}(0,1)$	bal	neg	0.0	4.14	1.08	3.50	**5.34**
$\mathcal{LN}(0,1)$	bal	pos	0.0	4.22	1.30	3.88	6.04
$\mathcal{LN}(0,1)$	unb	hom	0.0	3.50	0.60	3.04	3.86
$\mathcal{LN}(0,1)$	unb	neg	0.0	**5.62**	2.10	**4.84**	6.36
$\mathcal{LN}(0,1)$	unb	pos	0.0	3.86	0.76	3.46	**4.52**
$\mathcal{N}(0,1)$	bal	hom	0.0	**4.50**	1.32	3.92	3.52
$\mathcal{N}(0,1)$	bal	neg	0.0	**5.54**	1.92	**4.92**	**4.78**
$\mathcal{N}(0,1)$	bal	pos	0.0	6.28	1.54	5.96	5.94
$\mathcal{N}(0,1)$	unb	hom	0.0	6.08	1.38	**5.28**	4.30
$\mathcal{N}(0,1)$	unb	neg	0.0	7.02	1.98	6.04	**5.08**
$\mathcal{N}(0,1)$	unb	pos	0.0	5.68	1.02	**5.22**	**4.62**
t_2	bal	hom	0.0	3.14	1.54	2.88	**4.80**
t_2	bal	neg	0.0	3.38	2.16	2.84	**4.62**
t_2	bal	pos	0.0	2.92	1.24	2.74	**5.60**
t_2	unb	hom	0.0	3.46	0.94	3.06	4.02
t_2	unb	neg	0.0	3.96	1.22	3.28	**4.56**
t_2	unb	pos	0.0	3.28	0.86	3.10	**4.48**
t_3	bal	hom	0.0	3.80	1.52	3.34	**4.92**
t_3	bal	neg	0.0	3.54	1.52	2.82	**4.68**
t_3	bal	pos	0.0	4.14	1.12	3.96	5.94
t_3	unb	hom	0.0	**4.54**	1.42	3.92	**4.76**
t_3	unb	neg	0.0	**5.08**	1.62	4.10	**5.08**
t_3	unb	pos	0.0	4.38	1.06	3.94	**4.68**

Table 5: Empirical FWER in % of two-sided Dunnett-type tests with kernel estimator

Setting				Method			
Distribution	Design		δ	asymp. MCTP	boot. MCTP	B. asymp.	B. perm.
χ_3^2	bal	hom	0.0	**5.04**	3.72	**4.46**	4.38
χ_3^2	bal	neg	0.0	6.40	**4.58**	5.66	6.02
χ_3^2	bal	pos	0.0	**4.54**	3.58	4.32	**4.78**
χ_3^2	unb	hom	0.0	6.00	**5.32**	**5.12**	4.32
χ_3^2	unb	neg	0.0	7.44	6.58	6.38	6.20
χ_3^2	unb	pos	0.0	**4.76**	3.64	**4.50**	3.86
$\mathcal{LN}(0,1)$	bal	hom	0.0	3.78	3.68	3.44	4.40
$\mathcal{LN}(0,1)$	bal	neg	0.0	**5.26**	**5.08**	**4.68**	6.24
$\mathcal{LN}(0,1)$	bal	pos	0.0	2.82	2.96	2.62	4.16
$\mathcal{LN}(0,1)$	unb	hom	0.0	3.94	4.26	3.60	3.96
$\mathcal{LN}(0,1)$	unb	neg	0.0	6.52	6.08	5.80	7.06
$\mathcal{LN}(0,1)$	unb	pos	0.0	2.98	3.18	2.86	3.78
$\mathcal{N}(0,1)$	bal	hom	0.0	4.04	3.98	3.44	3.36
$\mathcal{N}(0,1)$	bal	neg	0.0	**4.82**	**4.94**	4.00	4.34
$\mathcal{N}(0,1)$	bal	pos	0.0	**5.36**	4.14	**5.00**	**5.46**
$\mathcal{N}(0,1)$	unb	hom	0.0	6.12	**4.96**	**5.02**	3.96
$\mathcal{N}(0,1)$	unb	neg	0.0	7.38	6.54	6.06	**5.08**
$\mathcal{N}(0,1)$	unb	pos	0.0	**5.52**	3.96	**4.86**	4.12
t_2	bal	hom	0.0	3.18	**4.44**	2.88	**4.60**
t_2	bal	neg	0.0	2.82	**4.88**	2.28	4.06
t_2	bal	pos	0.0	2.98	3.56	2.70	**5.20**
t_2	unb	hom	0.0	3.80	**4.62**	3.04	4.00
t_2	unb	neg	0.0	**4.44**	**5.60**	3.38	**4.52**
t_2	unb	pos	0.0	3.42	3.56	2.90	**4.52**
t_3	bal	hom	0.0	3.84	4.40	3.24	**4.62**
t_3	bal	neg	0.0	3.68	**4.68**	2.76	4.40
t_3	bal	pos	0.0	3.62	3.70	3.22	**5.22**
t_3	unb	hom	0.0	**4.66**	**5.00**	3.74	4.24
t_3	unb	neg	0.0	**5.50**	5.70	4.12	**4.66**
t_3	unb	pos	0.0	4.18	3.80	3.78	4.30

Table 6: Empirical FWER in % of non-inferiority Dunnett-type tests with kernel estimator

Setting				Method			
Distribution	Design		δ	asymp. MCTP	boot. MCTP	B. asymp.	B. perm.
χ^2_3	bal	hom	0.0	4.38	**4.78**	3.56	4.34
χ^2_3	bal	neg	0.0	**4.62**	**4.82**	3.60	**4.84**
χ^2_3	bal	pos	0.0	**4.76**	**4.78**	3.82	**5.20**
χ^2_3	unb	hom	0.0	**4.56**	**4.84**	3.78	4.14
χ^2_3	unb	neg	0.0	**5.40**	**5.44**	**4.58**	**5.24**
χ^2_3	unb	pos	0.0	4.14	4.24	3.32	4.24
$\mathcal{LN}(0,1)$	bal	hom	0.0	2.56	3.60	1.98	4.06
$\mathcal{LN}(0,1)$	bal	neg	0.0	2.56	3.76	1.98	**5.16**
$\mathcal{LN}(0,1)$	bal	pos	0.0	2.58	3.72	2.04	**4.76**
$\mathcal{LN}(0,1)$	unb	hom	0.0	2.80	3.60	2.18	4.06
$\mathcal{LN}(0,1)$	unb	neg	0.0	4.18	**5.24**	3.26	6.42
$\mathcal{LN}(0,1)$	unb	pos	0.0	2.84	3.96	2.10	**4.68**
$\mathcal{N}(0,1)$	bal	hom	0.0	4.04	**4.72**	3.40	3.46
$\mathcal{N}(0,1)$	bal	neg	0.0	**5.60**	5.80	**4.62**	**5.18**
$\mathcal{N}(0,1)$	bal	pos	0.0	**5.28**	**5.48**	4.40	**4.72**
$\mathcal{N}(0,1)$	unb	hom	0.0	**4.92**	**4.96**	3.94	3.94
$\mathcal{N}(0,1)$	unb	neg	0.0	**5.52**	**5.14**	**4.60**	**4.92**
$\mathcal{N}(0,1)$	unb	pos	0.0	**5.00**	**5.04**	4.08	**4.44**
t_2	bal	hom	0.0	2.82	**4.62**	2.30	4.22
t_2	bal	neg	0.0	3.32	**5.08**	2.84	**5.06**
t_2	bal	pos	0.0	2.94	**4.46**	2.28	**4.44**
t_2	unb	hom	0.0	2.64	4.00	2.12	4.02
t_2	unb	neg	0.0	3.04	4.32	2.22	**4.90**
t_2	unb	pos	0.0	2.96	**4.48**	2.20	**4.56**
t_3	bal	hom	0.0	3.92	**5.20**	3.24	**4.70**
t_3	bal	neg	0.0	3.10	**4.68**	2.40	**4.52**
t_3	bal	pos	0.0	3.84	**5.34**	2.98	**5.04**
t_3	unb	hom	0.0	3.40	**4.48**	2.84	**4.68**
t_3	unb	neg	0.0	3.86	**4.48**	3.04	**4.98**
t_3	unb	pos	0.0	3.20	**4.80**	2.66	**4.62**

Table 7: Empirical FWER in % of two-sided Tukey-type tests with bootstrap estimator

Setting				Method			
Distribution	Design		δ	asymp. MCTP	boot. MCTP	B. asymp.	B. perm.
χ_3^2	bal	hom	0.0	3.82	5.86	3.16	4.30
χ_3^2	bal	neg	0.0	**4.44**	6.34	3.66	**5.50**
χ_3^2	bal	pos	0.0	4.00	**5.48**	3.24	**4.52**
χ_3^2	unb	hom	0.0	**4.52**	7.04	3.84	4.14
χ_3^2	unb	neg	0.0	6.64	8.62	**5.50**	6.24
χ_3^2	unb	pos	0.0	4.08	6.20	3.46	3.96
$\mathcal{LN}(0,1)$	bal	hom	0.0	2.84	**5.24**	2.26	4.36
$\mathcal{LN}(0,1)$	bal	neg	0.0	3.10	5.84	2.72	5.74
$\mathcal{LN}(0,1)$	bal	pos	0.0	1.94	**4.52**	1.56	3.80
$\mathcal{LN}(0,1)$	unb	hom	0.0	3.10	5.70	2.60	3.98
$\mathcal{LN}(0,1)$	unb	neg	0.0	**5.50**	8.16	**4.44**	7.30
$\mathcal{LN}(0,1)$	unb	pos	0.0	2.68	**5.14**	2.16	3.80
$\mathcal{N}(0,1)$	bal	hom	0.0	3.46	**5.56**	2.92	3.70
$\mathcal{N}(0,1)$	bal	neg	0.0	**4.74**	6.32	4.02	**4.84**
$\mathcal{N}(0,1)$	bal	pos	0.0	4.32	5.72	3.56	**4.44**
$\mathcal{N}(0,1)$	unb	hom	0.0	**4.96**	6.68	4.04	3.86
$\mathcal{N}(0,1)$	unb	neg	0.0	5.72	7.54	**4.82**	**5.06**
$\mathcal{N}(0,1)$	unb	pos	0.0	**4.44**	6.34	3.86	3.98
t_2	bal	hom	0.0	2.78	6.02	2.34	**4.50**
t_2	bal	neg	0.0	2.92	5.94	2.58	**4.84**
t_2	bal	pos	0.0	2.70	**5.44**	2.14	**4.42**
t_2	unb	hom	0.0	2.92	5.86	2.38	4.36
t_2	unb	neg	0.0	3.70	6.60	2.96	**5.06**
t_2	unb	pos	0.0	2.68	5.82	2.30	4.30
t_3	bal	hom	0.0	3.38	6.02	2.94	**4.82**
t_3	bal	neg	0.0	2.96	5.98	2.34	**4.56**
t_3	bal	pos	0.0	3.24	5.86	2.82	**4.58**
t_3	unb	hom	0.0	3.82	6.74	3.14	**4.42**
t_3	unb	neg	0.0	4.24	7.36	3.30	**5.36**
t_3	unb	pos	0.0	3.66	6.22	2.98	**4.50**

Table 8: Empirical FWER in % of non-inferiority Tukey-type tests with bootstrap estimator

Setting				Method			
Distribution	Design		δ	asymp. MCTP	boot. MCTP	B. asymp.	B. perm.
χ^2_3	bal	hom	0.0	6.00	1.34	**4.56**	4.24
χ^2_3	bal	neg	0.0	7.28	1.50	6.18	6.04
χ^2_3	bal	pos	0.0	6.78	1.20	**5.36**	**5.04**
χ^2_3	unb	hom	0.0	**5.04**	1.46	4.02	4.20
χ^2_3	unb	neg	0.0	6.00	1.66	**4.86**	6.06
χ^2_3	unb	pos	0.0	**4.54**	0.92	3.68	**4.78**
$\mathcal{LN}(0,1)$	bal	hom	0.0	6.04	0.70	**4.66**	**4.48**
$\mathcal{LN}(0,1)$	bal	neg	0.0	6.46	1.00	**5.22**	**5.42**
$\mathcal{LN}(0,1)$	bal	pos	0.0	6.16	0.82	**4.88**	**5.18**
$\mathcal{LN}(0,1)$	unb	hom	0.0	**4.50**	0.78	3.68	4.06
$\mathcal{LN}(0,1)$	unb	neg	0.0	6.56	1.64	**5.30**	7.22
$\mathcal{LN}(0,1)$	unb	pos	0.0	4.32	0.76	3.20	**4.96**
$\mathcal{N}(0,1)$	bal	hom	0.0	3.12	0.94	2.48	3.60
$\mathcal{N}(0,1)$	bal	neg	0.0	4.34	1.66	3.58	**5.22**
$\mathcal{N}(0,1)$	bal	pos	0.0	4.38	1.78	3.78	**5.38**
$\mathcal{N}(0,1)$	unb	hom	0.0	3.30	0.92	2.70	4.14
$\mathcal{N}(0,1)$	unb	neg	0.0	3.14	1.26	2.46	**4.68**
$\mathcal{N}(0,1)$	unb	pos	0.0	3.12	0.98	2.44	**5.22**
t_2	bal	hom	0.0	2.12	1.18	1.78	**4.68**
t_2	bal	neg	0.0	2.66	1.34	2.12	**5.28**
t_2	bal	pos	0.0	1.82	1.04	1.50	**4.68**
t_2	unb	hom	0.0	1.82	0.92	1.46	4.16
t_2	unb	neg	0.0	1.90	0.70	1.40	**4.54**
t_2	unb	pos	0.0	1.74	0.88	1.38	**4.94**
t_3	bal	hom	0.0	2.74	1.04	2.26	4.38
t_3	bal	neg	0.0	2.58	1.00	1.80	**4.66**
t_3	bal	pos	0.0	3.04	1.30	2.34	**4.92**
t_3	unb	hom	0.0	2.28	1.02	1.70	**4.48**
t_3	unb	neg	0.0	2.44	1.04	1.82	**5.06**
t_3	unb	pos	0.0	2.28	0.88	1.76	**5.48**

Table 9: Empirical FWER in % of two-sided Tukey-type tests with interval-based estimator

Setting				Method			
Distribution	Design		δ	asymp. MCTP	boot. MCTP	B. asymp.	B. perm.
χ^2_3	bal	hom	0.0	**5.50**	3.04	**4.52**	4.06
χ^2_3	bal	neg	0.0	7.56	3.08	6.42	6.40
χ^2_3	bal	pos	0.0	**4.52**	2.40	3.62	3.54
χ^2_3	unb	hom	0.0	5.88	3.32	**4.94**	4.00
χ^2_3	unb	neg	0.0	8.12	3.88	6.92	7.08
χ^2_3	unb	pos	0.0	**4.48**	2.90	3.82	4.10
$\mathcal{LN}(0,1)$	bal	hom	0.0	5.86	2.82	**4.84**	**4.62**
$\mathcal{LN}(0,1)$	bal	neg	0.0	7.32	3.26	6.30	7.08
$\mathcal{LN}(0,1)$	bal	pos	0.0	3.96	1.66	3.12	3.32
$\mathcal{LN}(0,1)$	unb	hom	0.0	**5.16**	2.68	4.30	4.38
$\mathcal{LN}(0,1)$	unb	neg	0.0	8.18	4.34	7.34	8.52
$\mathcal{LN}(0,1)$	unb	pos	0.0	3.86	2.24	3.24	4.28
$\mathcal{N}(0,1)$	bal	hom	0.0	3.04	2.56	2.56	3.56
$\mathcal{N}(0,1)$	bal	neg	0.0	4.32	3.10	3.68	5.64
$\mathcal{N}(0,1)$	bal	pos	0.0	3.34	2.58	2.80	4.20
$\mathcal{N}(0,1)$	unb	hom	0.0	3.48	2.38	2.92	3.94
$\mathcal{N}(0,1)$	unb	neg	0.0	4.22	2.48	3.48	**5.00**
$\mathcal{N}(0,1)$	unb	pos	0.0	3.06	2.36	2.62	**4.54**
t_2	bal	hom	0.0	2.16	2.48	1.76	**4.64**
t_2	bal	neg	0.0	2.56	2.64	2.12	**5.28**
t_2	bal	pos	0.0	1.90	2.40	1.38	**4.44**
t_2	unb	hom	0.0	1.82	1.82	1.50	**4.42**
t_2	unb	neg	0.0	2.18	1.80	1.64	**4.96**
t_2	unb	pos	0.0	1.64	1.88	1.28	**5.02**
t_3	bal	hom	0.0	2.44	2.36	2.02	**4.56**
t_3	bal	neg	0.0	2.60	2.36	2.02	**4.96**
t_3	bal	pos	0.0	2.32	2.54	1.96	4.24
t_3	unb	hom	0.0	2.32	1.84	1.84	**4.46**
t_3	unb	neg	0.0	2.82	2.12	2.26	**5.14**
t_3	unb	pos	0.0	2.16	2.04	1.66	**4.88**

Table 10: Empirical FWER in % of non-inferiority Tukey-type tests with interval-based estimator

Setting				Method			
Distribution	Design		δ	asymp. MCTP	boot. MCTP	B. asymp.	B. perm.
χ_3^2	bal	hom	0.0	6.02	1.22	**4.92**	4.14
χ_3^2	bal	neg	0.0	7.22	1.48	5.66	5.92
χ_3^2	bal	pos	0.0	6.94	1.38	5.84	5.66
χ_3^2	unb	hom	0.0	6.66	1.16	**5.54**	4.24
χ_3^2	unb	neg	0.0	8.18	1.54	6.96	6.22
χ_3^2	unb	pos	0.0	6.12	0.84	**4.82**	4.12
$\mathcal{LN}(0,1)$	bal	hom	0.0	4.30	0.66	3.38	**4.58**
$\mathcal{LN}(0,1)$	bal	neg	0.0	**4.78**	1.00	3.58	5.76
$\mathcal{LN}(0,1)$	bal	pos	0.0	4.24	0.92	3.28	**5.42**
$\mathcal{LN}(0,1)$	unb	hom	0.0	4.18	0.78	3.38	3.92
$\mathcal{LN}(0,1)$	unb	neg	0.0	6.68	1.38	**5.46**	7.10
$\mathcal{LN}(0,1)$	unb	pos	0.0	4.12	0.72	3.38	**4.52**
$\mathcal{N}(0,1)$	bal	hom	0.0	**5.28**	0.96	**4.42**	3.44
$\mathcal{N}(0,1)$	bal	neg	0.0	6.92	1.48	**5.54**	**5.16**
$\mathcal{N}(0,1)$	bal	pos	0.0	6.54	1.54	**5.58**	**5.08**
$\mathcal{N}(0,1)$	unb	hom	0.0	7.40	1.34	6.22	4.24
$\mathcal{N}(0,1)$	unb	neg	0.0	7.72	1.48	6.58	**5.28**
$\mathcal{N}(0,1)$	unb	pos	0.0	6.36	1.32	**5.34**	4.20
t_2	bal	hom	0.0	2.94	1.40	2.52	**4.44**
t_2	bal	neg	0.0	3.64	1.28	3.04	**5.20**
t_2	bal	pos	0.0	3.18	1.28	2.42	**4.50**
t_2	unb	hom	0.0	3.70	0.96	2.86	3.86
t_2	unb	neg	0.0	4.40	0.86	3.42	**5.02**
t_2	unb	pos	0.0	3.40	0.84	2.68	4.24
t_3	bal	hom	0.0	4.34	1.32	3.68	**4.54**
t_3	bal	neg	0.0	3.66	1.12	2.78	**4.54**
t_3	bal	pos	0.0	4.02	1.14	3.30	**4.70**
t_3	unb	hom	0.0	**4.94**	1.34	4.18	**4.42**
t_3	unb	neg	0.0	5.64	1.24	**4.50**	**5.56**
t_3	unb	pos	0.0	4.34	1.00	3.48	4.12

Table 11: Empirical FWER in % of two-sided Tukey-type tests with kernel estimator

Setting				Method			
Distribution	Design		δ	asymp. MCTP	boot. MCTP	B. asymp.	B. perm.
χ_3^2	bal	hom	0.0	5.80	2.48	**4.96**	4.20
χ_3^2	bal	neg	0.0	7.12	2.32	5.98	6.58
χ_3^2	bal	pos	0.0	**5.00**	1.96	4.30	**4.50**
χ_3^2	unb	hom	0.0	6.46	3.12	**5.54**	4.38
χ_3^2	unb	neg	0.0	9.38	4.08	7.78	7.32
χ_3^2	unb	pos	0.0	**5.28**	2.48	**4.56**	3.94
$\mathcal{LN}(0,1)$	bal	hom	0.0	4.26	2.16	3.58	4.38
$\mathcal{LN}(0,1)$	bal	neg	0.0	**5.58**	2.70	**4.70**	7.12
$\mathcal{LN}(0,1)$	bal	pos	0.0	2.78	1.40	2.24	3.44
$\mathcal{LN}(0,1)$	unb	hom	0.0	**4.42**	2.34	3.76	3.94
$\mathcal{LN}(0,1)$	unb	neg	0.0	8.24	4.14	7.02	8.86
$\mathcal{LN}(0,1)$	unb	pos	0.0	3.26	2.00	2.64	3.40
$\mathcal{N}(0,1)$	bal	hom	0.0	**4.80**	1.94	4.10	3.48
$\mathcal{N}(0,1)$	bal	neg	0.0	6.26	2.34	**5.30**	**5.18**
$\mathcal{N}(0,1)$	bal	pos	0.0	5.82	2.44	**4.84**	**4.74**
$\mathcal{N}(0,1)$	unb	hom	0.0	6.54	2.54	**5.48**	4.00
$\mathcal{N}(0,1)$	unb	neg	0.0	7.54	2.82	6.50	**5.42**
$\mathcal{N}(0,1)$	unb	pos	0.0	5.78	2.24	**4.82**	4.20
t_2	bal	hom	0.0	3.08	2.64	2.54	**4.92**
t_2	bal	neg	0.0	3.22	2.26	2.78	**4.64**
t_2	bal	pos	0.0	2.88	2.14	2.38	**4.62**
t_2	unb	hom	0.0	3.84	2.12	3.20	4.06
t_2	unb	neg	0.0	**4.98**	2.50	3.90	**5.52**
t_2	unb	pos	0.0	3.56	1.76	2.76	4.18
t_3	bal	hom	0.0	3.64	2.26	2.90	**4.66**
t_3	bal	neg	0.0	3.52	1.86	2.84	**4.42**
t_3	bal	pos	0.0	3.44	2.04	2.84	**4.52**
t_3	unb	hom	0.0	**4.92**	2.46	4.08	**4.68**
t_3	unb	neg	0.0	**5.54**	2.70	**4.48**	**5.54**
t_3	unb	pos	0.0	**4.52**	2.10	3.72	4.32

Table 12: Empirical FWER in % of non-inferiority Tukey-type tests with kernel estimator

Setting				Method			
Distribution	Design		δ	asymp. MCTP	boot. MCTP	B. asymp.	B. perm.
χ^2_3	bal	hom	0.0	4.24	**5.24**	3.96	**5.14**
χ^2_3	bal	neg	0.0	4.14	**5.40**	3.96	**4.90**
χ^2_3	bal	pos	0.0	4.08	**5.60**	3.84	**5.02**
χ^2_3	unb	hom	0.0	3.88	**5.06**	3.50	4.30
χ^2_3	unb	neg	0.0	**4.42**	**5.56**	3.86	**5.18**
χ^2_3	unb	pos	0.0	4.24	**5.28**	3.72	**5.02**
$\mathcal{LN}(0,1)$	bal	hom	0.0	2.60	**4.64**	2.38	**4.80**
$\mathcal{LN}(0,1)$	bal	neg	0.0	2.56	4.32	2.40	**5.22**
$\mathcal{LN}(0,1)$	bal	pos	0.0	2.50	4.34	2.16	**5.18**
$\mathcal{LN}(0,1)$	unb	hom	0.0	2.44	3.74	2.24	4.08
$\mathcal{LN}(0,1)$	unb	neg	0.0	2.94	**5.12**	2.70	**5.60**
$\mathcal{LN}(0,1)$	unb	pos	0.0	2.48	4.22	2.26	**5.28**
$\mathcal{N}(0,1)$	bal	hom	0.0	3.96	**4.76**	3.64	4.06
$\mathcal{N}(0,1)$	bal	neg	0.0	**4.48**	**5.58**	4.12	**5.22**
$\mathcal{N}(0,1)$	bal	pos	0.0	**4.52**	**5.42**	4.06	**4.98**
$\mathcal{N}(0,1)$	unb	hom	0.0	**4.66**	**5.42**	4.40	**4.92**
$\mathcal{N}(0,1)$	unb	neg	0.0	**4.52**	**5.58**	4.16	**5.26**
$\mathcal{N}(0,1)$	unb	pos	0.0	4.18	**5.14**	4.00	**5.38**
t_2	bal	hom	0.0	2.70	5.86	2.54	**5.36**
t_2	bal	neg	0.0	2.80	5.64	2.60	**5.52**
t_2	bal	pos	0.0	2.80	**5.10**	2.54	**4.90**
t_2	unb	hom	0.0	2.22	4.40	2.02	**4.76**
t_2	unb	neg	0.0	2.28	**4.74**	2.00	**5.48**
t_2	unb	pos	0.0	2.32	**4.80**	2.16	5.72
t_3	bal	hom	0.0	3.34	**5.30**	3.00	**5.08**
t_3	bal	neg	0.0	2.90	**5.08**	2.62	**4.54**
t_3	bal	pos	0.0	3.38	**5.56**	2.96	**5.10**
t_3	unb	hom	0.0	2.90	**4.64**	2.60	**4.66**
t_3	unb	neg	0.0	3.40	**5.40**	3.04	**5.46**
t_3	unb	pos	0.0	2.98	**5.22**	2.70	**5.46**

Table 13: Empirical FWER in % of two-sided Grand-mean-type tests with bootstrap estimator

Setting				Method			
Distribution	Design		δ	asymp. MCTP	boot. MCTP	B. asymp.	B. perm.
χ^2_3	bal	hom	0.0	2.94	**4.78**	2.94	**4.94**
χ^2_3	bal	neg	0.0	2.70	**5.34**	2.70	6.20
χ^2_3	bal	pos	0.0	2.82	**4.78**	2.82	**5.54**
χ^2_3	unb	hom	0.0	2.76	**4.60**	2.72	**4.54**
χ^2_3	unb	neg	0.0	3.34	**5.60**	3.22	6.24
χ^2_3	unb	pos	0.0	2.98	**4.78**	2.94	**5.32**
$\mathcal{LN}(0,1)$	bal	hom	0.0	1.62	4.00	1.62	**4.80**
$\mathcal{LN}(0,1)$	bal	neg	0.0	1.68	**4.50**	1.66	5.72
$\mathcal{LN}(0,1)$	bal	pos	0.0	1.62	**4.44**	1.62	5.82
$\mathcal{LN}(0,1)$	unb	hom	0.0	1.72	3.74	1.72	4.34
$\mathcal{LN}(0,1)$	unb	neg	0.0	1.94	**4.50**	1.90	5.66
$\mathcal{LN}(0,1)$	unb	pos	0.0	1.74	3.82	1.74	5.76
$\mathcal{N}(0,1)$	bal	hom	0.0	3.46	4.36	3.46	4.38
$\mathcal{N}(0,1)$	bal	neg	0.0	4.26	**5.32**	4.24	**5.28**
$\mathcal{N}(0,1)$	bal	pos	0.0	3.94	**5.56**	3.90	**5.58**
$\mathcal{N}(0,1)$	unb	hom	0.0	4.16	**5.36**	4.14	**5.32**
$\mathcal{N}(0,1)$	unb	neg	0.0	4.30	**5.52**	4.22	**5.50**
$\mathcal{N}(0,1)$	unb	pos	0.0	4.04	**5.02**	4.02	**5.38**
t_2	bal	hom	0.0	2.54	**5.42**	2.52	**5.10**
t_2	bal	neg	0.0	2.76	5.70	2.74	5.68
t_2	bal	pos	0.0	2.38	**4.80**	2.38	**5.52**
t_2	unb	hom	0.0	2.20	**4.72**	2.16	**4.70**
t_2	unb	neg	0.0	2.30	**4.88**	2.26	**5.62**
t_2	unb	pos	0.0	2.22	**4.60**	2.20	5.76
t_3	bal	hom	0.0	3.08	**5.16**	3.04	**5.16**
t_3	bal	neg	0.0	2.88	**5.14**	2.88	**5.54**
t_3	bal	pos	0.0	3.12	**5.04**	3.08	**5.06**
t_3	unb	hom	0.0	3.28	**5.38**	3.26	**5.26**
t_3	unb	neg	0.0	3.40	5.84	3.34	6.12
t_3	unb	pos	0.0	3.40	**5.36**	3.40	6.00

Table 14: Empirical FWER in % of non-inferiority Grand-mean-type tests with bootstrap estimator

Setting				Method			
Distribution	Design		δ	asymp. MCTP	boot. MCTP	B. asymp.	B. perm.
χ_3^2	bal	hom	0.0	6.94	2.20	6.44	**4.72**
χ_3^2	bal	neg	0.0	7.66	2.48	7.02	5.86
χ_3^2	bal	pos	0.0	7.06	2.20	6.48	**5.28**
χ_3^2	unb	hom	0.0	**5.22**	1.52	**4.70**	4.32
χ_3^2	unb	neg	0.0	**5.10**	1.74	**4.56**	6.22
χ_3^2	unb	pos	0.0	**5.08**	1.64	**4.60**	6.12
$\mathcal{LN}(0,1)$	bal	hom	0.0	7.48	1.74	6.86	**4.84**
$\mathcal{LN}(0,1)$	bal	neg	0.0	7.28	1.76	6.66	**5.58**
$\mathcal{LN}(0,1)$	bal	pos	0.0	6.86	1.48	6.24	5.78
$\mathcal{LN}(0,1)$	unb	hom	0.0	4.06	1.50	3.78	4.32
$\mathcal{LN}(0,1)$	unb	neg	0.0	**5.26**	1.94	**4.76**	6.70
$\mathcal{LN}(0,1)$	unb	pos	0.0	4.16	1.58	3.56	5.92
$\mathcal{N}(0,1)$	bal	hom	0.0	3.26	1.16	2.94	4.38
$\mathcal{N}(0,1)$	bal	neg	0.0	3.90	2.32	3.72	**5.54**
$\mathcal{N}(0,1)$	bal	pos	0.0	4.12	1.86	3.80	**5.06**
$\mathcal{N}(0,1)$	unb	hom	0.0	2.84	1.10	2.54	**4.82**
$\mathcal{N}(0,1)$	unb	neg	0.0	2.14	1.02	1.92	**4.96**
$\mathcal{N}(0,1)$	unb	pos	0.0	2.80	1.22	2.52	6.08
t_2	bal	hom	0.0	2.06	1.62	1.92	**5.46**
t_2	bal	neg	0.0	2.10	1.80	1.86	5.70
t_2	bal	pos	0.0	1.60	1.36	1.30	**4.56**
t_2	unb	hom	0.0	0.68	0.46	0.54	**4.56**
t_2	unb	neg	0.0	0.78	0.36	0.68	**4.46**
t_2	unb	pos	0.0	1.06	0.86	0.98	6.56
t_3	bal	hom	0.0	2.30	1.56	2.10	**4.72**
t_3	bal	neg	0.0	2.16	1.24	1.96	**5.02**
t_3	bal	pos	0.0	2.52	1.66	2.20	**5.16**
t_3	unb	hom	0.0	1.26	0.62	1.12	**5.00**
t_3	unb	neg	0.0	1.34	0.68	1.16	**5.14**
t_3	unb	pos	0.0	1.50	0.76	1.36	6.42

Table 15: Empirical FWER in % of two-sided Grand-mean-type tests with interval-based estimator

Setting				Method			
Distribution	Design		δ	asymp. MCTP	boot. MCTP	B. asymp.	B. perm.
χ^2_3	bal	hom	0.0	3.56	1.40	3.54	**5.00**
χ^2_3	bal	neg	0.0	4.12	1.80	4.10	6.28
χ^2_3	bal	pos	0.0	3.52	1.52	3.48	**5.44**
χ^2_3	unb	hom	0.0	2.78	1.20	2.72	**5.02**
χ^2_3	unb	neg	0.0	3.46	1.44	3.44	6.02
χ^2_3	unb	pos	0.0	2.94	1.14	2.92	6.16
$\mathcal{LN}(0,1)$	bal	hom	0.0	3.06	0.96	3.04	**5.08**
$\mathcal{LN}(0,1)$	bal	neg	0.0	3.40	1.30	3.38	5.66
$\mathcal{LN}(0,1)$	bal	pos	0.0	2.92	1.02	2.80	**5.38**
$\mathcal{LN}(0,1)$	unb	hom	0.0	2.42	1.22	2.40	**4.46**
$\mathcal{LN}(0,1)$	unb	neg	0.0	2.80	1.30	2.76	6.30
$\mathcal{LN}(0,1)$	unb	pos	0.0	2.60	1.30	2.58	6.66
$\mathcal{N}(0,1)$	bal	hom	0.0	2.20	1.52	2.20	**4.42**
$\mathcal{N}(0,1)$	bal	neg	0.0	2.74	1.82	2.72	**5.62**
$\mathcal{N}(0,1)$	bal	pos	0.0	2.76	1.82	2.74	**5.18**
$\mathcal{N}(0,1)$	unb	hom	0.0	2.72	1.64	2.70	**5.10**
$\mathcal{N}(0,1)$	unb	neg	0.0	2.26	1.34	2.22	**5.54**
$\mathcal{N}(0,1)$	unb	pos	0.0	2.58	1.82	2.56	6.48
t_2	bal	hom	0.0	1.74	2.04	1.72	**4.80**
t_2	bal	neg	0.0	1.80	2.32	1.78	5.66
t_2	bal	pos	0.0	1.50	1.74	1.46	**5.26**
t_2	unb	hom	0.0	0.72	0.80	0.68	**4.72**
t_2	unb	neg	0.0	0.70	0.80	0.66	**4.62**
t_2	unb	pos	0.0	0.86	0.98	0.84	7.14
t_3	bal	hom	0.0	1.94	1.82	1.94	**5.12**
t_3	bal	neg	0.0	2.18	1.82	2.18	**5.62**
t_3	bal	pos	0.0	2.28	2.16	2.26	**5.22**
t_3	unb	hom	0.0	1.40	1.24	1.40	**5.30**
t_3	unb	neg	0.0	1.34	1.18	1.32	5.70
t_3	unb	pos	0.0	1.58	1.22	1.56	7.34

Table 16: Empirical FWER in % of non-inferiority Grand-mean-type tests with interval-based estimator

Setting				Method			
Distribution	Design		δ	asymp. MCTP	boot. MCTP	B. asymp.	B. perm.
χ^2_3	bal	hom	0.0	6.74	1.56	6.28	**4.78**
χ^2_3	bal	neg	0.0	7.30	1.68	6.68	5.74
χ^2_3	bal	pos	0.0	7.04	1.64	6.40	5.72
χ^2_3	unb	hom	0.0	6.48	1.38	6.10	4.40
χ^2_3	unb	neg	0.0	6.98	1.80	6.30	5.84
χ^2_3	unb	pos	0.0	6.40	1.42	5.92	**5.46**
$\mathcal{LN}(0,1)$	bal	hom	0.0	5.82	1.42	**5.20**	**4.92**
$\mathcal{LN}(0,1)$	bal	neg	0.0	5.72	1.48	**5.14**	5.86
$\mathcal{LN}(0,1)$	bal	pos	0.0	**5.36**	1.40	**4.82**	**5.58**
$\mathcal{LN}(0,1)$	unb	hom	0.0	4.10	1.40	3.66	4.32
$\mathcal{LN}(0,1)$	unb	neg	0.0	5.68	2.08	**5.06**	6.28
$\mathcal{LN}(0,1)$	unb	pos	0.0	4.20	1.20	3.78	**5.48**
$\mathcal{N}(0,1)$	bal	hom	0.0	**5.22**	1.06	**4.84**	4.36
$\mathcal{N}(0,1)$	bal	neg	0.0	5.78	1.76	**5.32**	**5.28**
$\mathcal{N}(0,1)$	bal	pos	0.0	6.48	1.44	5.86	5.88
$\mathcal{N}(0,1)$	unb	hom	0.0	7.02	1.42	6.58	**5.20**
$\mathcal{N}(0,1)$	unb	neg	0.0	6.62	1.58	6.06	**5.40**
$\mathcal{N}(0,1)$	unb	pos	0.0	6.12	1.52	**5.58**	**5.04**
t_2	bal	hom	0.0	3.14	1.70	2.82	**5.24**
t_2	bal	neg	0.0	3.10	1.52	2.74	**5.40**
t_2	bal	pos	0.0	2.66	1.52	2.38	**5.12**
t_2	unb	hom	0.0	3.60	1.38	3.28	**4.78**
t_2	unb	neg	0.0	3.92	1.34	3.48	**5.58**
t_2	unb	pos	0.0	3.12	1.26	2.80	**4.76**
t_3	bal	hom	0.0	3.98	1.40	3.60	**5.38**
t_3	bal	neg	0.0	3.52	1.24	3.06	**4.56**
t_3	bal	pos	0.0	3.98	1.30	3.66	**5.16**
t_3	unb	hom	0.0	**4.44**	1.40	4.10	**4.80**
t_3	unb	neg	0.0	**5.26**	1.60	**4.70**	5.78
t_3	unb	pos	0.0	4.14	1.24	3.72	**4.98**

Table 17: Empirical FWER in % of two-sided Grand-mean-type tests with kernel estimator

Setting				Method			
Distribution	Design		δ	asymp. MCTP	boot. MCTP	B. asymp.	B. perm.
χ_3^2	bal	hom	0.0	3.94	1.50	3.92	**4.78**
χ_3^2	bal	neg	0.0	4.30	1.84	4.28	6.64
χ_3^2	bal	pos	0.0	3.96	1.58	3.90	5.78
χ_3^2	unb	hom	0.0	4.24	1.32	4.20	**4.88**
χ_3^2	unb	neg	0.0	**5.24**	1.56	**5.18**	7.12
χ_3^2	unb	pos	0.0	4.04	1.60	4.00	**4.98**
$\mathcal{LN}(0,1)$	bal	hom	0.0	2.16	1.14	2.16	**5.14**
$\mathcal{LN}(0,1)$	bal	neg	0.0	2.44	1.36	2.40	5.76
$\mathcal{LN}(0,1)$	bal	pos	0.0	2.44	1.42	2.44	5.90
$\mathcal{LN}(0,1)$	unb	hom	0.0	2.34	1.18	2.34	**4.44**
$\mathcal{LN}(0,1)$	unb	neg	0.0	3.04	1.42	3.00	6.58
$\mathcal{LN}(0,1)$	unb	pos	0.0	2.32	1.06	2.32	**5.44**
$\mathcal{N}(0,1)$	bal	hom	0.0	**4.62**	1.42	**4.56**	4.20
$\mathcal{N}(0,1)$	bal	neg	0.0	**5.36**	1.70	**5.32**	**5.50**
$\mathcal{N}(0,1)$	bal	pos	0.0	**5.26**	1.84	**5.24**	6.00
$\mathcal{N}(0,1)$	unb	hom	0.0	6.76	1.90	6.72	**5.28**
$\mathcal{N}(0,1)$	unb	neg	0.0	6.66	1.54	6.58	5.92
$\mathcal{N}(0,1)$	unb	pos	0.0	6.52	1.92	6.50	**5.46**
t_2	bal	hom	0.0	2.88	1.84	2.88	**4.96**
t_2	bal	neg	0.0	3.26	2.14	3.20	5.74
t_2	bal	pos	0.0	2.84	1.64	2.78	5.74
t_2	unb	hom	0.0	3.46	1.52	3.46	**4.68**
t_2	unb	neg	0.0	3.72	1.22	3.62	5.96
t_2	unb	pos	0.0	3.26	1.30	3.26	**5.36**
t_3	bal	hom	0.0	3.82	1.92	3.80	**5.40**
t_3	bal	neg	0.0	3.38	1.74	3.38	5.72
t_3	bal	pos	0.0	3.26	1.82	3.24	**5.04**
t_3	unb	hom	0.0	**4.74**	1.56	**4.68**	**5.30**
t_3	unb	neg	0.0	**5.26**	1.86	**5.18**	7.08
t_3	unb	pos	0.0	**4.48**	1.40	**4.48**	**5.36**

Table 18: Empirical FWER in % of non-inferiority Grand-mean-type tests with kernel estimator

Setting				Method			
Distribution	Design		δ	asymp. MCTP	boot. MCTP	B. asymp.	B. perm.
χ_3^2	bal	hom	0.5	5.46	6.56	5.02	6.64
χ_3^2	bal	neg	0.5	5.22	6.98	4.78	6.42
χ_3^2	bal	pos	0.5	4.60	5.24	4.38	5.98
χ_3^2	unb	hom	0.5	5.94	7.50	5.18	6.74
χ_3^2	unb	neg	0.5	6.82	8.26	6.10	8.02
χ_3^2	unb	pos	0.5	4.18	5.02	3.98	5.80
$\mathcal{LN}(0,1)$	bal	hom	0.5	7.48	10.58	7.00	12.72
$\mathcal{LN}(0,1)$	bal	neg	0.5	5.68	8.62	5.20	10.22
$\mathcal{LN}(0,1)$	bal	pos	0.5	4.18	5.60	4.00	8.70
$\mathcal{LN}(0,1)$	unb	hom	0.5	10.26	14.02	9.28	15.60
$\mathcal{LN}(0,1)$	unb	neg	0.5	9.52	12.06	8.58	13.24
$\mathcal{LN}(0,1)$	unb	pos	0.5	4.66	6.54	4.38	8.46
$\mathcal{N}(0,1)$	bal	hom	0.5	11.66	13.02	10.74	12.42
$\mathcal{N}(0,1)$	bal	neg	0.5	7.50	9.32	6.68	7.84
$\mathcal{N}(0,1)$	bal	pos	0.5	8.12	8.32	7.82	9.78
$\mathcal{N}(0,1)$	unb	hom	0.5	12.32	13.48	11.08	11.74
$\mathcal{N}(0,1)$	unb	neg	0.5	8.92	10.46	7.62	8.66
$\mathcal{N}(0,1)$	unb	pos	0.5	8.40	9.10	7.84	9.42
t_2	bal	hom	0.5	6.40	10.26	5.62	9.90
t_2	bal	neg	0.5	4.48	8.60	3.64	7.90
t_2	bal	pos	0.5	4.44	6.92	4.16	8.18
t_2	unb	hom	0.5	6.72	10.04	5.92	10.08
t_2	unb	neg	0.5	4.90	7.66	4.06	7.36
t_2	unb	pos	0.5	3.66	6.48	3.44	7.78
t_3	bal	hom	0.5	8.30	11.16	7.60	10.58
t_3	bal	neg	0.5	5.90	8.86	5.24	7.56
t_3	bal	pos	0.5	5.40	7.52	5.16	8.68
t_3	unb	hom	0.5	8.06	10.86	7.18	10.42
t_3	unb	neg	0.5	6.28	8.70	5.14	7.90
t_3	unb	pos	0.5	5.62	7.78	5.08	8.32

Table 19: Empirical Power for $\delta = 0.5$ in % of two-sided Dunnett-type tests with bootstrap estimator

Setting				Method			
Distribution	Design		δ	asymp. MCTP	boot. MCTP	B. asymp.	B. perm.
χ^2_3	bal	hom	0.5	6.94	11.86	6.14	8.52
χ^2_3	bal	neg	0.5	5.96	11.34	5.28	7.54
χ^2_3	bal	pos	0.5	4.60	8.48	4.26	6.46
χ^2_3	unb	hom	0.5	8.16	13.66	7.06	8.14
χ^2_3	unb	neg	0.5	9.00	14.20	7.88	9.06
χ^2_3	unb	pos	0.5	5.24	9.70	4.96	5.78
$\mathcal{LN}(0,1)$	bal	hom	0.5	10.56	18.98	9.72	16.48
$\mathcal{LN}(0,1)$	bal	neg	0.5	8.26	15.48	7.32	13.10
$\mathcal{LN}(0,1)$	bal	pos	0.5	5.60	11.98	5.34	10.94
$\mathcal{LN}(0,1)$	unb	hom	0.5	15.20	23.94	14.00	17.98
$\mathcal{LN}(0,1)$	unb	neg	0.5	12.86	19.62	11.54	14.92
$\mathcal{LN}(0,1)$	unb	pos	0.5	6.84	14.10	6.36	10.28
$\mathcal{N}(0,1)$	bal	hom	0.5	15.76	21.90	14.26	17.36
$\mathcal{N}(0,1)$	bal	neg	0.5	9.82	16.20	8.20	10.12
$\mathcal{N}(0,1)$	bal	pos	0.5	10.64	14.98	9.82	12.82
$\mathcal{N}(0,1)$	unb	hom	0.5	17.00	23.78	14.52	14.76
$\mathcal{N}(0,1)$	unb	neg	0.5	12.40	19.64	9.92	10.08
$\mathcal{N}(0,1)$	unb	pos	0.5	11.04	15.94	10.22	10.76
t_2	bal	hom	0.5	9.62	18.52	8.48	13.64
t_2	bal	neg	0.5	6.48	14.48	5.28	9.20
t_2	bal	pos	0.5	6.26	13.26	5.80	10.52
t_2	unb	hom	0.5	9.68	19.94	8.32	11.72
t_2	unb	neg	0.5	6.84	15.66	5.44	7.80
t_2	unb	pos	0.5	5.82	13.42	5.14	9.84
t_3	bal	hom	0.5	11.46	19.74	10.22	14.10
t_3	bal	neg	0.5	8.42	15.80	6.84	10.10
t_3	bal	pos	0.5	7.70	13.90	7.18	11.52
t_3	unb	hom	0.5	11.36	19.62	9.68	11.94
t_3	unb	neg	0.5	8.72	17.36	6.80	8.66
t_3	unb	pos	0.5	7.58	14.60	6.68	9.46

Table 20: Empirical Power for $\delta = 0.5$ in % of non-inferiority Dunnett-type tests with bootstrap estimator

Setting				Method			
Distribution	Design		δ	asymp. MCTP	boot. MCTP	B. asymp.	B. perm.
χ^2_3	bal	hom	0.5	7.72	2.44	6.92	6.64
χ^2_3	bal	neg	0.5	7.78	3.34	7.04	7.78
χ^2_3	bal	pos	0.5	6.46	1.72	6.18	6.10
χ^2_3	unb	hom	0.5	6.56	2.46	5.78	6.92
χ^2_3	unb	neg	0.5	7.98	3.76	6.88	9.44
χ^2_3	unb	pos	0.5	5.38	1.68	4.96	6.48
$\mathcal{LN}(0,1)$	bal	hom	0.5	14.32	4.50	13.20	14.90
$\mathcal{LN}(0,1)$	bal	neg	0.5	12.14	4.74	10.62	13.34
$\mathcal{LN}(0,1)$	bal	pos	0.5	8.22	1.68	7.92	8.46
$\mathcal{LN}(0,1)$	unb	hom	0.5	14.52	6.70	13.44	17.10
$\mathcal{LN}(0,1)$	unb	neg	0.5	13.58	6.86	12.20	16.38
$\mathcal{LN}(0,1)$	unb	pos	0.5	6.64	2.08	6.22	8.82
$\mathcal{N}(0,1)$	bal	hom	0.5	10.06	5.78	9.32	12.38
$\mathcal{N}(0,1)$	bal	neg	0.5	6.62	3.78	5.78	8.64
$\mathcal{N}(0,1)$	bal	pos	0.5	5.92	2.60	5.60	8.24
$\mathcal{N}(0,1)$	unb	hom	0.5	7.88	4.40	6.82	11.34
$\mathcal{N}(0,1)$	unb	neg	0.5	5.50	3.20	4.48	8.70
$\mathcal{N}(0,1)$	unb	pos	0.5	5.40	2.78	5.04	10.28
t_2	bal	hom	0.5	4.46	3.00	4.10	9.12
t_2	bal	neg	0.5	3.10	2.12	2.78	7.64
t_2	bal	pos	0.5	2.94	1.76	2.80	7.42
t_2	unb	hom	0.5	3.02	2.06	2.54	8.64
t_2	unb	neg	0.5	1.78	1.34	1.54	5.96
t_2	unb	pos	0.5	1.58	1.04	1.52	7.26
t_3	bal	hom	0.5	6.30	4.38	5.90	10.36
t_3	bal	neg	0.5	4.70	3.12	4.00	8.52
t_3	bal	pos	0.5	4.16	2.28	3.94	7.80
t_3	unb	hom	0.5	3.64	2.24	3.22	8.94
t_3	unb	neg	0.5	2.66	1.56	2.12	6.58
t_3	unb	pos	0.5	2.56	1.52	2.30	8.30

Table 21: Empirical Power for $\delta = 0.5$ in % of two-sided Dunnett-type tests with interval-based estimator

Setting				Method			
Distribution	Design		δ	asymp. MCTP	boot. MCTP	B. asymp.	B. perm.
χ^2_3	bal	hom	0.5	9.38	8.48	8.82	8.58
χ^2_3	bal	neg	0.5	9.06	8.00	8.00	9.18
χ^2_3	bal	pos	0.5	6.32	5.56	6.22	5.96
χ^2_3	unb	hom	0.5	9.40	9.32	8.30	7.58
χ^2_3	unb	neg	0.5	10.82	10.96	9.30	10.46
χ^2_3	unb	pos	0.5	6.42	6.52	5.82	6.30
$\mathcal{LN}(0,1)$	bal	hom	0.5	18.94	13.88	17.56	19.00
$\mathcal{LN}(0,1)$	bal	neg	0.5	15.24	11.64	13.74	16.50
$\mathcal{LN}(0,1)$	bal	pos	0.5	10.36	7.46	9.74	9.98
$\mathcal{LN}(0,1)$	unb	hom	0.5	20.28	17.74	18.62	19.04
$\mathcal{LN}(0,1)$	unb	neg	0.5	17.42	15.42	15.70	17.44
$\mathcal{LN}(0,1)$	unb	pos	0.5	9.50	9.04	8.80	9.70
$\mathcal{N}(0,1)$	bal	hom	0.5	14.08	15.42	12.38	16.62
$\mathcal{N}(0,1)$	bal	neg	0.5	9.40	11.50	7.94	11.14
$\mathcal{N}(0,1)$	bal	pos	0.5	8.06	9.82	7.60	10.90
$\mathcal{N}(0,1)$	unb	hom	0.5	12.30	14.76	10.66	13.68
$\mathcal{N}(0,1)$	unb	neg	0.5	8.56	11.80	6.60	10.04
$\mathcal{N}(0,1)$	unb	pos	0.5	8.04	9.72	7.30	11.18
t_2	bal	hom	0.5	7.22	11.46	6.40	12.36
t_2	bal	neg	0.5	4.80	9.10	3.76	9.46
t_2	bal	pos	0.5	4.74	7.86	4.38	10.26
t_2	unb	hom	0.5	5.10	9.24	4.32	10.58
t_2	unb	neg	0.5	3.26	6.38	2.50	6.86
t_2	unb	pos	0.5	2.68	5.58	2.40	9.08
t_3	bal	hom	0.5	8.96	12.94	7.98	13.66
t_3	bal	neg	0.5	6.96	10.54	5.80	10.36
t_3	bal	pos	0.5	5.72	9.02	5.24	10.24
t_3	unb	hom	0.5	6.24	9.86	4.98	11.00
t_3	unb	neg	0.5	4.38	8.84	3.44	7.38
t_3	unb	pos	0.5	4.22	7.46	3.62	10.28

Table 22: Empirical Power for $\delta = 0.5$ in % of non-inferiority Dunnett-type tests with interval-based estimator

Setting				Method			
Distribution	Design		δ	asymp. MCTP	boot. MCTP	B. asymp.	B. perm.
χ^2_3	bal	hom	0.5	7.60	1.92	7.04	6.44
χ^2_3	bal	neg	0.5	7.80	2.68	6.94	7.36
χ^2_3	bal	pos	0.5	6.88	1.58	6.62	6.60
χ^2_3	unb	hom	0.5	8.30	1.98	7.56	6.56
χ^2_3	unb	neg	0.5	9.86	3.50	8.72	9.00
χ^2_3	unb	pos	0.5	5.96	1.54	5.72	5.48
$\mathcal{LN}(0,1)$	bal	hom	0.5	12.08	4.02	11.22	15.22
$\mathcal{LN}(0,1)$	bal	neg	0.5	9.56	4.16	8.76	13.16
$\mathcal{LN}(0,1)$	bal	pos	0.5	6.22	1.36	5.86	8.88
$\mathcal{LN}(0,1)$	unb	hom	0.5	15.14	5.56	14.26	17.62
$\mathcal{LN}(0,1)$	unb	neg	0.5	14.74	6.54	13.38	16.40
$\mathcal{LN}(0,1)$	unb	pos	0.5	6.28	1.62	5.90	7.66
$\mathcal{N}(0,1)$	bal	hom	0.5	13.62	4.86	12.66	12.08
$\mathcal{N}(0,1)$	bal	neg	0.5	9.16	3.36	7.76	7.84
$\mathcal{N}(0,1)$	bal	pos	0.5	9.08	2.38	8.66	9.44
$\mathcal{N}(0,1)$	unb	hom	0.5	15.08	4.68	13.58	11.60
$\mathcal{N}(0,1)$	unb	neg	0.5	11.64	3.72	9.90	8.56
$\mathcal{N}(0,1)$	unb	pos	0.5	10.72	2.70	10.12	8.70
t_2	bal	hom	0.5	6.50	3.32	5.98	9.72
t_2	bal	neg	0.5	4.82	2.38	3.94	7.52
t_2	bal	pos	0.5	4.76	1.90	4.42	8.10
t_2	unb	hom	0.5	8.74	3.38	7.72	10.04
t_2	unb	neg	0.5	6.92	2.86	5.86	7.66
t_2	unb	pos	0.5	5.18	1.98	4.92	6.80
t_3	bal	hom	0.5	8.72	4.20	8.30	10.54
t_3	bal	neg	0.5	6.58	3.26	5.72	7.60
t_3	bal	pos	0.5	6.36	2.26	6.02	8.64
t_3	unb	hom	0.5	10.20	3.34	9.18	10.06
t_3	unb	neg	0.5	8.06	3.10	6.82	7.88
t_3	unb	pos	0.5	6.68	2.08	6.20	7.40

Table 23: Empirical Power for $\delta = 0.5$ in % of two-sided Dunnett-type tests with kernel estimator

Setting				Method			
Distribution	Design		δ	asymp. MCTP	boot. MCTP	B. asymp.	B. perm.
χ^2_3	bal	hom	0.5	9.04	6.64	8.38	8.34
χ^2_3	bal	neg	0.5	8.94	7.20	7.84	9.06
χ^2_3	bal	pos	0.5	6.26	4.60	5.90	6.20
χ^2_3	unb	hom	0.5	10.24	8.02	9.30	8.18
χ^2_3	unb	neg	0.5	11.72	9.66	10.16	10.04
χ^2_3	unb	pos	0.5	6.62	5.62	6.30	5.60
$\mathcal{LN}(0,1)$	bal	hom	0.5	16.42	13.44	15.28	19.82
$\mathcal{LN}(0,1)$	bal	neg	0.5	13.64	11.30	11.82	16.64
$\mathcal{LN}(0,1)$	bal	pos	0.5	7.56	6.66	7.28	10.24
$\mathcal{LN}(0,1)$	unb	hom	0.5	20.66	17.10	18.94	20.94
$\mathcal{LN}(0,1)$	unb	neg	0.5	18.42	15.34	16.78	18.60
$\mathcal{LN}(0,1)$	unb	pos	0.5	8.38	7.98	7.88	9.40
$\mathcal{N}(0,1)$	bal	hom	0.5	17.86	13.46	16.38	16.82
$\mathcal{N}(0,1)$	bal	neg	0.5	11.48	10.58	9.60	10.10
$\mathcal{N}(0,1)$	bal	pos	0.5	12.26	8.38	11.44	12.48
$\mathcal{N}(0,1)$	unb	hom	0.5	19.90	13.64	17.40	14.68
$\mathcal{N}(0,1)$	unb	neg	0.5	14.58	11.94	11.72	10.32
$\mathcal{N}(0,1)$	unb	pos	0.5	12.72	8.58	11.82	10.88
t_2	bal	hom	0.5	9.90	11.10	8.74	13.48
t_2	bal	neg	0.5	6.60	8.80	5.52	9.02
t_2	bal	pos	0.5	6.84	7.14	6.20	10.78
t_2	unb	hom	0.5	12.64	11.84	10.78	12.36
t_2	unb	neg	0.5	8.94	9.34	7.12	8.60
t_2	unb	pos	0.5	6.72	7.06	6.12	8.78
t_3	bal	hom	0.5	12.14	12.28	10.80	14.16
t_3	bal	neg	0.5	8.86	9.84	7.38	10.20
t_3	bal	pos	0.5	8.48	7.82	7.76	11.38
t_3	unb	hom	0.5	13.72	11.48	11.84	12.30
t_3	unb	neg	0.5	10.82	10.30	8.88	9.38
t_3	unb	pos	0.5	8.88	7.46	7.90	9.08

Table 24: Empirical Power for $\delta = 0.5$ in % of non-inferiority Dunnett-type tests with kernel estimator

Setting				Method			
Distribution	Design		δ	asymp. MCTP	boot. MCTP	B. asymp.	B. perm.
χ^2_3	bal	hom	0.5	6.98	7.30	5.66	6.88
χ^2_3	bal	neg	0.5	7.32	7.24	6.06	8.00
χ^2_3	bal	pos	0.5	4.86	5.20	3.86	5.06
χ^2_3	unb	hom	0.5	8.44	8.64	7.24	8.28
χ^2_3	unb	neg	0.5	10.08	9.78	8.60	10.24
χ^2_3	unb	pos	0.5	5.10	5.72	4.26	5.08
$\mathcal{LN}(0,1)$	bal	hom	0.5	12.18	14.28	10.30	16.90
$\mathcal{LN}(0,1)$	bal	neg	0.5	10.62	12.58	9.22	16.56
$\mathcal{LN}(0,1)$	bal	pos	0.5	4.94	6.04	4.12	8.08
$\mathcal{LN}(0,1)$	unb	hom	0.5	17.84	19.96	15.60	23.54
$\mathcal{LN}(0,1)$	unb	neg	0.5	17.80	19.30	15.54	23.64
$\mathcal{LN}(0,1)$	unb	pos	0.5	6.64	8.04	5.50	9.38
$\mathcal{N}(0,1)$	bal	hom	0.5	16.38	16.56	14.08	15.28
$\mathcal{N}(0,1)$	bal	neg	0.5	12.80	11.68	10.94	11.98
$\mathcal{N}(0,1)$	bal	pos	0.5	8.72	8.90	7.26	8.90
$\mathcal{N}(0,1)$	unb	hom	0.5	19.40	17.62	16.90	17.16
$\mathcal{N}(0,1)$	unb	neg	0.5	17.34	14.96	14.60	15.48
$\mathcal{N}(0,1)$	unb	pos	0.5	10.06	9.82	8.72	9.26
t_2	bal	hom	0.5	9.08	12.40	7.78	12.84
t_2	bal	neg	0.5	7.12	10.14	5.74	10.14
t_2	bal	pos	0.5	5.00	7.84	3.84	7.68
t_2	unb	hom	0.5	10.94	13.24	9.30	13.56
t_2	unb	neg	0.5	9.24	10.96	7.60	11.64
t_2	unb	pos	0.5	5.16	7.60	4.18	7.62
t_3	bal	hom	0.5	12.04	14.54	10.40	13.46
t_3	bal	neg	0.5	9.16	11.04	8.04	10.66
t_3	bal	pos	0.5	6.60	8.12	5.66	8.06
t_3	unb	hom	0.5	13.20	14.88	11.04	14.42
t_3	unb	neg	0.5	11.74	12.44	9.76	12.66
t_3	unb	pos	0.5	7.78	9.60	6.48	9.06

Table 25: Empirical Power for $\delta = 0.5$ in % of two-sided Tukey-type tests with bootstrap estimator

Setting				Method			
Distribution	Design		δ	asymp. MCTP	boot. MCTP	B. asymp.	B. perm.
χ^2_3	bal	hom	0.5	8.84	11.86	7.70	9.80
χ^2_3	bal	neg	0.5	8.74	11.06	7.58	10.68
χ^2_3	bal	pos	0.5	5.20	7.68	4.32	5.98
χ^2_3	unb	hom	0.5	11.96	15.18	10.10	10.86
χ^2_3	unb	neg	0.5	13.54	15.30	11.78	13.34
χ^2_3	unb	pos	0.5	6.48	9.84	5.56	6.40
$\mathcal{LN}(0,1)$	bal	hom	0.5	16.74	24.26	14.86	24.18
$\mathcal{LN}(0,1)$	bal	neg	0.5	14.82	20.36	12.62	23.04
$\mathcal{LN}(0,1)$	bal	pos	0.5	6.92	12.50	5.62	11.18
$\mathcal{LN}(0,1)$	unb	hom	0.5	24.48	31.90	21.24	30.10
$\mathcal{LN}(0,1)$	unb	neg	0.5	24.18	28.60	21.08	30.10
$\mathcal{LN}(0,1)$	unb	pos	0.5	9.82	16.22	8.18	12.60
$\mathcal{N}(0,1)$	bal	hom	0.5	22.32	25.24	20.16	22.82
$\mathcal{N}(0,1)$	bal	neg	0.5	17.08	19.74	15.16	18.24
$\mathcal{N}(0,1)$	bal	pos	0.5	11.86	14.54	10.44	12.90
$\mathcal{N}(0,1)$	unb	hom	0.5	26.58	28.74	23.86	23.92
$\mathcal{N}(0,1)$	unb	neg	0.5	23.58	25.32	20.76	21.42
$\mathcal{N}(0,1)$	unb	pos	0.5	14.30	16.22	12.58	12.68
t_2	bal	hom	0.5	12.92	20.88	11.30	18.46
t_2	bal	neg	0.5	10.04	17.14	8.42	15.24
t_2	bal	pos	0.5	7.20	12.56	5.96	11.20
t_2	unb	hom	0.5	15.60	23.06	13.36	18.58
t_2	unb	neg	0.5	13.32	19.78	11.00	15.98
t_2	unb	pos	0.5	7.38	13.12	6.42	10.88
t_3	bal	hom	0.5	16.58	22.82	14.60	19.22
t_3	bal	neg	0.5	13.44	18.68	11.66	17.06
t_3	bal	pos	0.5	9.20	14.22	7.94	12.42
t_3	unb	hom	0.5	18.98	24.52	16.86	20.24
t_3	unb	neg	0.5	16.48	21.74	14.10	17.56
t_3	unb	pos	0.5	11.26	15.22	9.48	12.68

Table 26: Empirical Power for $\delta = 0.5$ in % of non-inferiority Tukey-type tests with bootstrap estimator

Setting				Method			
Distribution	Design		δ	asymp. MCTP	boot. MCTP	B. asymp.	B. perm.
χ_3^2	bal	hom	0.5	9.76	2.36	8.30	7.46
χ_3^2	bal	neg	0.5	11.14	2.76	9.64	9.70
χ_3^2	bal	pos	0.5	7.02	1.38	5.52	5.14
χ_3^2	unb	hom	0.5	9.12	3.02	7.48	7.74
χ_3^2	unb	neg	0.5	12.22	4.12	10.38	12.94
χ_3^2	unb	pos	0.5	6.34	1.52	5.14	6.00
$\mathcal{LN}(0,1)$	bal	hom	0.5	22.72	6.62	20.14	20.84
$\mathcal{LN}(0,1)$	bal	neg	0.5	21.38	7.28	18.76	21.46
$\mathcal{LN}(0,1)$	bal	pos	0.5	10.06	1.76	8.28	8.42
$\mathcal{LN}(0,1)$	unb	hom	0.5	25.60	9.96	22.68	26.72
$\mathcal{LN}(0,1)$	unb	neg	0.5	24.86	10.44	22.18	28.20
$\mathcal{LN}(0,1)$	unb	pos	0.5	9.46	2.44	7.78	9.78
$\mathcal{N}(0,1)$	bal	hom	0.5	14.38	6.36	12.30	15.52
$\mathcal{N}(0,1)$	bal	neg	0.5	11.92	4.96	10.14	13.94
$\mathcal{N}(0,1)$	bal	pos	0.5	6.56	2.66	5.56	7.72
$\mathcal{N}(0,1)$	unb	hom	0.5	15.70	7.36	13.52	17.22
$\mathcal{N}(0,1)$	unb	neg	0.5	14.12	5.78	11.76	15.60
$\mathcal{N}(0,1)$	unb	pos	0.5	7.78	3.00	6.34	10.16
t_2	bal	hom	0.5	6.48	3.48	5.34	12.06
t_2	bal	neg	0.5	5.18	2.72	4.26	10.20
t_2	bal	pos	0.5	3.70	1.84	2.92	6.98
t_2	unb	hom	0.5	7.16	4.12	5.48	12.28
t_2	unb	neg	0.5	5.88	3.22	4.62	10.18
t_2	unb	pos	0.5	3.20	1.90	2.64	7.54
t_3	bal	hom	0.5	8.98	5.12	7.62	12.60
t_3	bal	neg	0.5	7.42	3.94	5.90	11.42
t_3	bal	pos	0.5	5.14	2.36	4.08	8.06
t_3	unb	hom	0.5	9.56	4.90	7.92	13.36
t_3	unb	neg	0.5	8.12	4.10	6.68	12.14
t_3	unb	pos	0.5	4.78	2.52	4.08	9.14

Table 27: Empirical Power for $\delta = 0.5$ in % of two-sided Tukey-type tests with interval-based estimator

Setting				Method			
Distribution	Design		δ	asymp. MCTP	boot. MCTP	B. asymp.	B. perm.
χ^2_3	bal	hom	0.5	12.74	6.70	11.08	10.38
χ^2_3	bal	neg	0.5	14.52	7.02	12.62	13.66
χ^2_3	bal	pos	0.5	7.22	4.08	6.02	5.74
χ^2_3	unb	hom	0.5	13.88	7.84	11.72	10.18
χ^2_3	unb	neg	0.5	16.56	8.42	14.54	16.02
χ^2_3	unb	pos	0.5	8.14	4.90	6.94	6.42
$\mathcal{LN}(0,1)$	bal	hom	0.5	29.72	16.62	26.76	28.76
$\mathcal{LN}(0,1)$	bal	neg	0.5	27.06	13.92	24.64	28.94
$\mathcal{LN}(0,1)$	bal	pos	0.5	12.82	6.98	10.90	11.40
$\mathcal{LN}(0,1)$	unb	hom	0.5	33.60	20.14	30.32	32.84
$\mathcal{LN}(0,1)$	unb	neg	0.5	32.56	18.98	29.02	34.26
$\mathcal{LN}(0,1)$	unb	pos	0.5	13.54	8.54	11.70	12.84
$\mathcal{N}(0,1)$	bal	hom	0.5	19.80	15.72	17.50	23.38
$\mathcal{N}(0,1)$	bal	neg	0.5	16.70	12.04	15.00	20.90
$\mathcal{N}(0,1)$	bal	pos	0.5	9.18	7.30	7.88	11.30
$\mathcal{N}(0,1)$	unb	hom	0.5	22.50	15.44	19.72	22.90
$\mathcal{N}(0,1)$	unb	neg	0.5	19.94	12.66	17.08	21.34
$\mathcal{N}(0,1)$	unb	pos	0.5	10.80	7.92	9.64	13.12
t_2	bal	hom	0.5	9.94	10.34	8.88	17.56
t_2	bal	neg	0.5	7.94	7.62	6.54	15.98
t_2	bal	pos	0.5	5.36	5.66	4.28	10.54
t_2	unb	hom	0.5	11.06	10.18	9.30	16.92
t_2	unb	neg	0.5	9.00	8.38	7.24	14.52
t_2	unb	pos	0.5	4.88	4.82	4.08	10.84
t_3	bal	hom	0.5	12.76	12.04	11.54	18.32
t_3	bal	neg	0.5	10.48	9.38	9.16	17.24
t_3	bal	pos	0.5	7.00	7.10	5.98	11.22
t_3	unb	hom	0.5	13.44	11.06	11.66	19.02
t_3	unb	neg	0.5	12.14	9.40	10.02	16.40
t_3	unb	pos	0.5	7.34	6.52	6.04	12.52

Table 28: Empirical Power for $\delta = 0.5$ in % of non-inferiority Tukey-type tests with interval-based estimator

Setting				Method			
Distribution	Design		δ	asymp. MCTP	boot. MCTP	B. asymp.	B. perm.
χ_3^2	bal	hom	0.5	9.38	1.90	8.06	7.02
χ_3^2	bal	neg	0.5	11.22	2.36	9.24	9.60
χ_3^2	bal	pos	0.5	7.40	1.26	5.86	5.62
χ_3^2	unb	hom	0.5	10.94	1.92	9.26	7.88
χ_3^2	unb	neg	0.5	14.06	3.18	12.28	12.30
χ_3^2	unb	pos	0.5	7.12	1.36	6.00	4.94
$\mathcal{LN}(0,1)$	bal	hom	0.5	18.64	5.56	15.96	20.68
$\mathcal{LN}(0,1)$	bal	neg	0.5	17.76	5.42	15.76	21.58
$\mathcal{LN}(0,1)$	bal	pos	0.5	7.18	1.50	5.84	8.20
$\mathcal{LN}(0,1)$	unb	hom	0.5	25.14	6.94	21.96	28.56
$\mathcal{LN}(0,1)$	unb	neg	0.5	25.88	8.12	22.48	30.82
$\mathcal{LN}(0,1)$	unb	pos	0.5	8.62	1.88	7.40	9.24
$\mathcal{N}(0,1)$	bal	hom	0.5	18.86	5.70	16.70	15.22
$\mathcal{N}(0,1)$	bal	neg	0.5	15.18	4.34	13.20	12.06
$\mathcal{N}(0,1)$	bal	pos	0.5	10.36	2.56	8.84	8.56
$\mathcal{N}(0,1)$	unb	hom	0.5	22.74	5.46	20.10	17.08
$\mathcal{N}(0,1)$	unb	neg	0.5	20.38	4.22	17.52	15.54
$\mathcal{N}(0,1)$	unb	pos	0.5	12.62	2.62	11.10	8.94
t_2	bal	hom	0.5	9.52	3.98	7.64	12.74
t_2	bal	neg	0.5	7.50	2.84	6.14	10.34
t_2	bal	pos	0.5	5.28	2.16	4.30	7.56
t_2	unb	hom	0.5	12.58	3.56	10.50	13.36
t_2	unb	neg	0.5	10.36	2.74	8.60	12.04
t_2	unb	pos	0.5	6.20	2.02	5.22	7.08
t_3	bal	hom	0.5	12.24	4.70	10.76	12.94
t_3	bal	neg	0.5	9.96	3.64	8.30	10.80
t_3	bal	pos	0.5	7.52	2.42	6.14	8.30
t_3	unb	hom	0.5	14.52	4.16	12.24	14.34
t_3	unb	neg	0.5	13.10	3.62	10.90	12.96
t_3	unb	pos	0.5	8.54	2.10	7.08	8.12

Table 29: Empirical Power for $\delta = 0.5$ in % of two-sided Tukey-type tests with kernel estimator

Setting				Method			
Distribution	Design		δ	asymp. MCTP	boot. MCTP	B. asymp.	B. perm.
χ_3^2	bal	hom	0.5	12.04	5.02	10.34	9.78
χ_3^2	bal	neg	0.5	13.68	5.44	11.94	12.90
χ_3^2	bal	pos	0.5	7.26	3.18	6.04	5.86
χ_3^2	unb	hom	0.5	14.92	7.14	12.74	11.34
χ_3^2	unb	neg	0.5	17.78	7.84	15.64	15.96
χ_3^2	unb	pos	0.5	8.22	4.28	7.06	6.20
$\mathcal{LN}(0,1)$	bal	hom	0.5	24.94	14.70	22.22	29.16
$\mathcal{LN}(0,1)$	bal	neg	0.5	23.42	12.22	21.04	30.38
$\mathcal{LN}(0,1)$	bal	pos	0.5	9.90	5.36	7.92	11.58
$\mathcal{LN}(0,1)$	unb	hom	0.5	33.76	19.72	30.30	37.12
$\mathcal{LN}(0,1)$	unb	neg	0.5	33.58	19.08	30.12	39.22
$\mathcal{LN}(0,1)$	unb	pos	0.5	12.08	7.74	10.26	13.12
$\mathcal{N}(0,1)$	bal	hom	0.5	25.00	12.50	22.40	21.76
$\mathcal{N}(0,1)$	bal	neg	0.5	19.58	9.82	17.40	18.08
$\mathcal{N}(0,1)$	bal	pos	0.5	13.54	6.12	11.80	12.84
$\mathcal{N}(0,1)$	unb	hom	0.5	29.08	13.34	26.74	24.24
$\mathcal{N}(0,1)$	unb	neg	0.5	26.66	11.74	23.28	22.02
$\mathcal{N}(0,1)$	unb	pos	0.5	16.22	7.20	14.60	12.44
t_2	bal	hom	0.5	13.90	9.80	12.12	18.84
t_2	bal	neg	0.5	10.52	7.34	8.84	15.30
t_2	bal	pos	0.5	7.28	5.22	6.20	10.98
t_2	unb	hom	0.5	17.12	10.18	15.10	18.80
t_2	unb	neg	0.5	14.68	8.46	12.34	16.20
t_2	unb	pos	0.5	8.42	4.90	7.42	9.98
t_3	bal	hom	0.5	16.84	11.24	15.26	18.82
t_3	bal	neg	0.5	14.28	8.56	12.38	16.68
t_3	bal	pos	0.5	10.10	6.26	8.92	12.38
t_3	unb	hom	0.5	20.74	10.76	18.36	20.20
t_3	unb	neg	0.5	17.86	9.60	15.36	17.84
t_3	unb	pos	0.5	11.54	5.78	10.20	11.50

Table 30: Empirical Power for $\delta = 0.5$ in % of non-inferiority Tukey-type tests with kernel estimator

Setting				Method			
Distribution	Design		δ	asymp. MCTP	boot. MCTP	B. asymp.	B. perm.
χ_3^2	bal	hom	0.5	6.26	7.28	5.76	7.24
χ_3^2	bal	neg	0.5	6.10	7.14	5.34	7.16
χ_3^2	bal	pos	0.5	4.34	5.70	3.88	5.20
χ_3^2	unb	hom	0.5	6.72	8.34	6.30	8.42
χ_3^2	unb	neg	0.5	6.84	8.28	6.14	8.84
χ_3^2	unb	pos	0.5	5.38	6.54	4.94	6.46
$\mathcal{LN}(0,1)$	bal	hom	0.5	9.56	14.68	8.96	16.00
$\mathcal{LN}(0,1)$	bal	neg	0.5	7.12	10.76	6.54	13.20
$\mathcal{LN}(0,1)$	bal	pos	0.5	5.28	7.80	4.72	9.32
$\mathcal{LN}(0,1)$	unb	hom	0.5	12.40	18.18	11.46	21.46
$\mathcal{LN}(0,1)$	unb	neg	0.5	9.94	14.80	9.18	18.46
$\mathcal{LN}(0,1)$	unb	pos	0.5	5.60	8.88	5.28	10.86
$\mathcal{N}(0,1)$	bal	hom	0.5	15.96	16.86	15.14	17.26
$\mathcal{N}(0,1)$	bal	neg	0.5	10.80	11.72	9.94	12.30
$\mathcal{N}(0,1)$	bal	pos	0.5	8.12	9.26	7.68	9.24
$\mathcal{N}(0,1)$	unb	hom	0.5	18.30	18.26	17.14	20.02
$\mathcal{N}(0,1)$	unb	neg	0.5	14.10	14.56	13.12	15.86
$\mathcal{N}(0,1)$	unb	pos	0.5	9.46	10.18	9.00	11.32
t_2	bal	hom	0.5	8.66	13.78	7.90	13.20
t_2	bal	neg	0.5	6.00	10.66	5.30	10.18
t_2	bal	pos	0.5	4.82	8.70	4.40	8.48
t_2	unb	hom	0.5	9.40	14.58	8.58	15.54
t_2	unb	neg	0.5	6.20	11.02	5.34	11.34
t_2	unb	pos	0.5	4.42	8.22	4.12	9.30
t_3	bal	hom	0.5	11.78	15.20	11.18	14.62
t_3	bal	neg	0.5	8.28	11.44	7.74	11.58
t_3	bal	pos	0.5	6.36	9.12	5.80	9.42
t_3	unb	hom	0.5	11.98	15.20	11.02	16.14
t_3	unb	neg	0.5	8.64	11.88	7.72	11.94
t_3	unb	pos	0.5	7.32	10.14	6.76	11.00

Table 31: Empirical Power for $\delta = 0.5$ in % of two-sided Grand-mean-type tests with bootstrap estimator

Setting				Method			
Distribution	Design		δ	asymp. MCTP	boot. MCTP	B. asymp.	B. perm.
χ^2_3	bal	hom	0.5	4.58	6.94	4.58	7.74
χ^2_3	bal	neg	0.5	4.54	7.32	4.50	8.14
χ^2_3	bal	pos	0.5	3.36	5.66	3.36	6.26
χ^2_3	unb	hom	0.5	5.52	8.86	5.48	8.98
χ^2_3	unb	neg	0.5	5.78	8.76	5.72	8.92
χ^2_3	unb	pos	0.5	3.74	5.90	3.72	6.78
$\mathcal{LN}(0,1)$	bal	hom	0.5	8.32	16.08	8.30	18.58
$\mathcal{LN}(0,1)$	bal	neg	0.5	5.86	12.90	5.80	16.36
$\mathcal{LN}(0,1)$	bal	pos	0.5	3.46	6.92	3.36	9.36
$\mathcal{LN}(0,1)$	unb	hom	0.5	13.02	21.72	12.94	24.64
$\mathcal{LN}(0,1)$	unb	neg	0.5	9.96	17.48	9.82	20.78
$\mathcal{LN}(0,1)$	unb	pos	0.5	4.76	9.52	4.68	11.82
$\mathcal{N}(0,1)$	bal	hom	0.5	16.62	18.86	16.56	19.74
$\mathcal{N}(0,1)$	bal	neg	0.5	11.32	13.12	11.22	13.44
$\mathcal{N}(0,1)$	bal	pos	0.5	8.50	10.12	8.50	11.00
$\mathcal{N}(0,1)$	unb	hom	0.5	20.04	21.02	19.94	21.38
$\mathcal{N}(0,1)$	unb	neg	0.5	16.14	17.46	15.90	17.12
$\mathcal{N}(0,1)$	unb	pos	0.5	10.04	10.92	10.00	11.40
t_2	bal	hom	0.5	9.32	14.96	9.32	15.62
t_2	bal	neg	0.5	6.72	11.38	6.66	11.86
t_2	bal	pos	0.5	5.42	9.24	5.34	10.14
t_2	unb	hom	0.5	11.38	16.58	11.28	16.58
t_2	unb	neg	0.5	7.50	12.70	7.32	12.06
t_2	unb	pos	0.5	5.16	9.08	5.14	10.30
t_3	bal	hom	0.5	11.80	16.00	11.76	16.24
t_3	bal	neg	0.5	9.48	13.12	9.44	13.44
t_3	bal	pos	0.5	6.38	9.34	6.34	10.32
t_3	unb	hom	0.5	13.34	17.70	13.20	17.26
t_3	unb	neg	0.5	9.92	14.70	9.76	13.26
t_3	unb	pos	0.5	7.92	11.10	7.90	11.16

Table 32: Empirical Power for $\delta = 0.5$ in % of non-inferiority Grand-mean-type tests with bootstrap estimator

Setting				Method			
Distribution	Design		δ	asymp. MCTP	boot. MCTP	B. asymp.	B. perm.
χ_3^2	bal	hom	0.5	10.30	3.24	9.68	7.22
χ_3^2	bal	neg	0.5	9.68	3.14	8.98	8.90
χ_3^2	bal	pos	0.5	7.96	1.98	7.24	5.66
χ_3^2	unb	hom	0.5	8.20	3.14	7.36	8.16
χ_3^2	unb	neg	0.5	8.94	3.36	8.02	11.76
χ_3^2	unb	pos	0.5	7.02	2.60	6.54	8.00
$\mathcal{LN}(0,1)$	bal	hom	0.5	20.46	7.14	19.46	17.84
$\mathcal{LN}(0,1)$	bal	neg	0.5	16.68	5.82	15.32	16.92
$\mathcal{LN}(0,1)$	bal	pos	0.5	12.50	3.72	11.26	10.02
$\mathcal{LN}(0,1)$	unb	hom	0.5	18.94	8.58	17.66	24.74
$\mathcal{LN}(0,1)$	unb	neg	0.5	15.48	7.26	14.36	22.72
$\mathcal{LN}(0,1)$	unb	pos	0.5	9.46	4.10	8.80	11.76
$\mathcal{N}(0,1)$	bal	hom	0.5	13.92	7.22	12.88	16.84
$\mathcal{N}(0,1)$	bal	neg	0.5	10.24	5.48	9.76	14.16
$\mathcal{N}(0,1)$	bal	pos	0.5	6.42	3.26	5.92	8.52
$\mathcal{N}(0,1)$	unb	hom	0.5	13.58	7.32	12.68	19.94
$\mathcal{N}(0,1)$	unb	neg	0.5	9.70	4.78	8.58	16.00
$\mathcal{N}(0,1)$	unb	pos	0.5	7.14	3.68	6.64	12.76
t_2	bal	hom	0.5	5.28	4.32	4.88	12.32
t_2	bal	neg	0.5	4.16	3.30	3.74	11.02
t_2	bal	pos	0.5	3.06	2.50	2.76	8.18
t_2	unb	hom	0.5	4.06	3.26	3.70	13.16
t_2	unb	neg	0.5	1.90	1.42	1.64	8.74
t_2	unb	pos	0.5	1.78	1.54	1.70	9.84
t_3	bal	hom	0.5	8.50	6.58	7.98	13.56
t_3	bal	neg	0.5	6.06	4.14	5.48	11.34
t_3	bal	pos	0.5	4.50	3.08	4.04	8.78
t_3	unb	hom	0.5	5.52	3.92	4.94	14.86
t_3	unb	neg	0.5	3.46	2.34	3.06	10.78
t_3	unb	pos	0.5	3.84	2.64	3.50	11.74

Table 33: Empirical Power for $\delta = 0.5$ in % of two-sided Grand-mean-type tests with interval-based estimator

Setting				Method			
Distribution	Design		δ	asymp. MCTP	boot. MCTP	B. asymp.	B. perm.
χ^2_3	bal	hom	0.5	5.84	2.82	5.84	7.96
χ^2_3	bal	neg	0.5	6.44	3.00	6.42	9.52
χ^2_3	bal	pos	0.5	4.26	1.74	4.18	6.06
χ^2_3	unb	hom	0.5	5.86	3.44	5.86	9.30
χ^2_3	unb	neg	0.5	6.66	3.64	6.58	10.58
χ^2_3	unb	pos	0.5	4.56	2.00	4.50	7.12
$\mathcal{LN}(0,1)$	bal	hom	0.5	15.50	8.56	15.44	21.92
$\mathcal{LN}(0,1)$	bal	neg	0.5	11.96	6.90	11.86	20.66
$\mathcal{LN}(0,1)$	bal	pos	0.5	6.50	3.00	6.40	9.56
$\mathcal{LN}(0,1)$	unb	hom	0.5	18.42	11.96	18.28	28.14
$\mathcal{LN}(0,1)$	unb	neg	0.5	14.08	9.74	13.86	24.44
$\mathcal{LN}(0,1)$	unb	pos	0.5	6.76	3.92	6.72	11.74
$\mathcal{N}(0,1)$	bal	hom	0.5	13.84	10.98	13.76	20.80
$\mathcal{N}(0,1)$	bal	neg	0.5	9.76	7.16	9.72	16.38
$\mathcal{N}(0,1)$	bal	pos	0.5	5.74	4.24	5.74	9.82
$\mathcal{N}(0,1)$	unb	hom	0.5	16.06	12.30	16.02	21.40
$\mathcal{N}(0,1)$	unb	neg	0.5	11.62	8.98	11.58	17.82
$\mathcal{N}(0,1)$	unb	pos	0.5	7.50	5.66	7.42	12.14
t_2	bal	hom	0.5	6.70	7.38	6.62	14.80
t_2	bal	neg	0.5	4.18	4.82	4.16	12.72
t_2	bal	pos	0.5	3.40	3.60	3.40	9.18
t_2	unb	hom	0.5	5.18	5.46	5.06	15.00
t_2	unb	neg	0.5	2.70	2.70	2.64	10.16
t_2	unb	pos	0.5	2.38	2.82	2.30	11.32
t_3	bal	hom	0.5	8.70	8.34	8.66	15.94
t_3	bal	neg	0.5	6.12	5.74	6.04	14.02
t_3	bal	pos	0.5	4.28	4.02	4.26	10.02
t_3	unb	hom	0.5	7.44	7.22	7.38	16.30
t_3	unb	neg	0.5	4.42	4.22	4.32	12.00
t_3	unb	pos	0.5	4.36	4.36	4.32	12.74

Table 34: Empirical Power for $\delta = 0.5$ in % of non-inferiority Grand-mean-type tests with interval-based estimator

Setting				Method			
Distribution	Design		δ	asymp. MCTP	boot. MCTP	B. asymp.	B. perm.
χ^2_3	bal	hom	0.5	9.78	2.52	9.36	7.26
χ^2_3	bal	neg	0.5	9.76	2.26	8.92	8.38
χ^2_3	bal	pos	0.5	7.70	1.58	7.18	5.94
χ^2_3	unb	hom	0.5	9.62	2.62	8.88	7.82
χ^2_3	unb	neg	0.5	10.68	2.86	9.68	10.50
χ^2_3	unb	pos	0.5	7.96	2.32	7.42	6.52
$\mathcal{LN}(0,1)$	bal	hom	0.5	18.00	5.80	16.90	18.44
$\mathcal{LN}(0,1)$	bal	neg	0.5	13.30	4.28	12.32	16.62
$\mathcal{LN}(0,1)$	bal	pos	0.5	9.84	2.62	9.06	9.88
$\mathcal{LN}(0,1)$	unb	hom	0.5	19.34	6.60	18.04	25.10
$\mathcal{LN}(0,1)$	unb	neg	0.5	16.00	6.38	14.64	23.74
$\mathcal{LN}(0,1)$	unb	pos	0.5	9.10	3.08	8.54	10.98
$\mathcal{N}(0,1)$	bal	hom	0.5	19.04	6.38	17.98	17.10
$\mathcal{N}(0,1)$	bal	neg	0.5	13.54	4.50	12.56	12.50
$\mathcal{N}(0,1)$	bal	pos	0.5	10.18	2.70	9.52	9.42
$\mathcal{N}(0,1)$	unb	hom	0.5	21.56	5.74	20.24	19.28
$\mathcal{N}(0,1)$	unb	neg	0.5	18.14	4.48	16.88	16.16
$\mathcal{N}(0,1)$	unb	pos	0.5	11.34	3.08	10.88	10.60
t_2	bal	hom	0.5	8.78	4.48	8.10	13.10
t_2	bal	neg	0.5	6.38	3.18	5.94	9.90
t_2	bal	pos	0.5	5.02	2.54	4.64	8.68
t_2	unb	hom	0.5	11.48	4.58	10.60	15.48
t_2	unb	neg	0.5	8.50	3.12	7.82	12.22
t_2	unb	pos	0.5	5.32	2.22	5.00	8.64
t_3	bal	hom	0.5	11.96	5.76	11.46	13.78
t_3	bal	neg	0.5	8.70	3.90	8.18	11.40
t_3	bal	pos	0.5	7.02	2.68	6.56	9.38
t_3	unb	hom	0.5	13.52	5.00	12.70	16.72
t_3	unb	neg	0.5	10.96	3.46	9.90	12.88
t_3	unb	pos	0.5	8.14	2.66	7.62	10.30

Table 35: Empirical Power for $\delta = 0.5$ in % of two-sided Grand-mean-type tests with kernel estimator

Setting				Method			
Distribution	Design		δ	asymp. MCTP	boot. MCTP	B. asymp.	B. perm.
χ^2_3	bal	hom	0.5	6.10	2.54	6.08	7.80
χ^2_3	bal	neg	0.5	6.68	3.10	6.70	9.42
χ^2_3	bal	pos	0.5	4.62	1.70	4.54	6.20
χ^2_3	unb	hom	0.5	7.08	2.70	7.04	9.44
χ^2_3	unb	neg	0.5	8.10	3.08	8.02	10.86
χ^2_3	unb	pos	0.5	5.24	1.82	5.18	6.52
$\mathcal{LN}(0,1)$	bal	hom	0.5	12.66	7.52	12.60	22.22
$\mathcal{LN}(0,1)$	bal	neg	0.5	9.72	6.50	9.66	20.84
$\mathcal{LN}(0,1)$	bal	pos	0.5	4.72	2.66	4.66	9.68
$\mathcal{LN}(0,1)$	unb	hom	0.5	17.80	9.30	17.62	30.66
$\mathcal{LN}(0,1)$	unb	neg	0.5	14.92	8.24	14.84	27.52
$\mathcal{LN}(0,1)$	unb	pos	0.5	5.64	2.90	5.56	11.80
$\mathcal{N}(0,1)$	bal	hom	0.5	19.16	9.02	19.10	19.54
$\mathcal{N}(0,1)$	bal	neg	0.5	13.34	5.58	13.32	14.04
$\mathcal{N}(0,1)$	bal	pos	0.5	10.12	4.36	10.08	10.80
$\mathcal{N}(0,1)$	unb	hom	0.5	22.66	7.80	22.64	21.60
$\mathcal{N}(0,1)$	unb	neg	0.5	20.20	5.70	20.00	18.32
$\mathcal{N}(0,1)$	unb	pos	0.5	11.28	3.70	11.26	11.30
t_2	bal	hom	0.5	10.06	6.64	10.04	15.78
t_2	bal	neg	0.5	7.12	4.40	7.10	12.20
t_2	bal	pos	0.5	5.74	3.72	5.68	10.06
t_2	unb	hom	0.5	13.08	6.06	13.02	16.52
t_2	unb	neg	0.5	9.98	3.82	9.80	13.46
t_2	unb	pos	0.5	5.72	2.78	5.70	9.36
t_3	bal	hom	0.5	12.12	7.68	12.12	15.86
t_3	bal	neg	0.5	9.88	5.36	9.88	13.50
t_3	bal	pos	0.5	6.96	4.06	6.96	10.62
t_3	unb	hom	0.5	15.40	6.22	15.36	17.90
t_3	unb	neg	0.5	13.00	4.48	12.80	14.16
t_3	unb	pos	0.5	8.72	3.34	8.64	10.70

Table 36: Empirical Power for $\delta = 0.5$ in % of non-inferiority Grand-mean-type tests with kernel estimator

Setting				Method			
Distribution	Design		δ	asymp. MCTP	boot. MCTP	B. asymp.	B. perm.
χ^2_3	bal	hom	1.0	9.90	11.32	9.14	12.22
χ^2_3	bal	neg	1.0	8.36	10.10	7.62	9.92
χ^2_3	bal	pos	1.0	5.94	6.66	5.58	8.18
χ^2_3	unb	hom	1.0	12.08	13.46	11.08	13.06
χ^2_3	unb	neg	1.0	10.38	12.12	9.16	11.58
χ^2_3	unb	pos	1.0	5.98	6.48	5.66	7.48
$\mathcal{LN}(0,1)$	bal	hom	1.0	30.82	36.26	29.38	38.16
$\mathcal{LN}(0,1)$	bal	neg	1.0	17.84	23.16	16.44	23.22
$\mathcal{LN}(0,1)$	bal	pos	1.0	12.96	15.22	12.52	21.64
$\mathcal{LN}(0,1)$	unb	hom	1.0	37.30	42.82	35.30	40.60
$\mathcal{LN}(0,1)$	unb	neg	1.0	22.98	27.50	20.76	24.98
$\mathcal{LN}(0,1)$	unb	pos	1.0	15.88	19.80	15.12	23.80
$\mathcal{N}(0,1)$	bal	hom	1.0	37.26	37.20	35.42	39.30
$\mathcal{N}(0,1)$	bal	neg	1.0	20.64	22.56	18.46	20.76
$\mathcal{N}(0,1)$	bal	pos	1.0	20.00	19.94	19.32	23.18
$\mathcal{N}(0,1)$	unb	hom	1.0	38.94	38.58	35.90	38.38
$\mathcal{N}(0,1)$	unb	neg	1.0	22.30	23.76	19.80	20.74
$\mathcal{N}(0,1)$	unb	pos	1.0	21.60	21.70	20.32	22.72
t_2	bal	hom	1.0	23.54	30.64	22.26	29.96
t_2	bal	neg	1.0	11.98	17.80	10.48	15.68
t_2	bal	pos	1.0	11.62	16.20	11.14	19.08
t_2	unb	hom	1.0	20.32	26.80	18.38	25.72
t_2	unb	neg	1.0	11.30	15.66	9.86	14.10
t_2	unb	pos	1.0	11.50	16.24	10.64	17.94
t_3	bal	hom	1.0	29.68	34.14	27.76	34.18
t_3	bal	neg	1.0	15.04	19.42	13.32	17.48
t_3	bal	pos	1.0	14.74	17.64	14.04	20.42
t_3	unb	hom	1.0	27.70	31.72	25.42	30.72
t_3	unb	neg	1.0	14.90	18.52	13.04	16.12
t_3	unb	pos	1.0	14.68	18.38	13.74	19.78

Table 37: Empirical Power for $\delta = 1.0$ in % of two-sided Dunnett-type tests with bootstrap estimator

Setting				Method			
Distribution	Design		δ	asymp. MCTP	boot. MCTP	B. asymp.	B. perm.
χ_3^2	bal	hom	1.0	13.64	19.90	12.52	16.28
χ_3^2	bal	neg	1.0	10.86	15.84	9.74	13.20
χ_3^2	bal	pos	1.0	8.24	13.26	7.60	10.98
χ_3^2	unb	hom	1.0	16.36	23.38	14.74	15.56
χ_3^2	unb	neg	1.0	13.96	20.12	12.02	13.82
χ_3^2	unb	pos	1.0	8.50	14.08	7.76	8.78
$\mathcal{LN}(0,1)$	bal	hom	1.0	39.24	49.94	37.00	47.06
$\mathcal{LN}(0,1)$	bal	neg	1.0	24.44	32.64	21.60	29.64
$\mathcal{LN}(0,1)$	bal	pos	1.0	19.04	29.60	18.18	28.24
$\mathcal{LN}(0,1)$	unb	hom	1.0	45.72	54.54	42.66	44.40
$\mathcal{LN}(0,1)$	unb	neg	1.0	29.16	37.94	26.34	27.50
$\mathcal{LN}(0,1)$	unb	pos	1.0	23.14	33.76	21.82	28.06
$\mathcal{N}(0,1)$	bal	hom	1.0	47.08	51.90	43.88	49.18
$\mathcal{N}(0,1)$	bal	neg	1.0	28.12	34.20	24.22	27.94
$\mathcal{N}(0,1)$	bal	pos	1.0	26.30	32.02	25.00	30.54
$\mathcal{N}(0,1)$	unb	hom	1.0	49.36	54.30	45.20	44.92
$\mathcal{N}(0,1)$	unb	neg	1.0	29.74	37.76	25.32	24.92
$\mathcal{N}(0,1)$	unb	pos	1.0	28.92	34.98	26.76	27.76
t_2	bal	hom	1.0	32.20	43.74	29.62	39.40
t_2	bal	neg	1.0	17.44	29.74	14.74	21.26
t_2	bal	pos	1.0	16.98	27.36	15.82	25.12
t_2	unb	hom	1.0	29.64	42.94	25.96	32.26
t_2	unb	neg	1.0	16.22	30.04	13.38	17.32
t_2	unb	pos	1.0	16.96	27.18	15.56	22.74
t_3	bal	hom	1.0	38.02	47.04	35.54	42.34
t_3	bal	neg	1.0	20.76	31.30	17.80	23.28
t_3	bal	pos	1.0	20.42	28.50	19.26	26.42
t_3	unb	hom	1.0	36.98	47.44	33.12	37.56
t_3	unb	neg	1.0	20.82	31.66	17.70	20.06
t_3	unb	pos	1.0	21.26	30.58	19.48	24.40

Table 38: Empirical Power for $\delta = 1.0$ in % of non-inferiority Dunnett-type tests with bootstrap estimator

Setting				Method			
Distribution	Design		δ	asymp. MCTP	boot. MCTP	B. asymp.	B. perm.
χ_3^2	bal	hom	1.0	14.38	5.22	13.34	13.78
χ_3^2	bal	neg	1.0	12.52	5.42	11.10	12.78
χ_3^2	bal	pos	1.0	8.00	1.60	7.54	7.70
χ_3^2	unb	hom	1.0	14.36	6.70	13.18	14.94
χ_3^2	unb	neg	1.0	11.90	6.12	10.30	14.42
χ_3^2	unb	pos	1.0	7.06	2.12	6.68	8.30
$\mathcal{LN}(0,1)$	bal	hom	1.0	46.82	26.24	44.74	45.96
$\mathcal{LN}(0,1)$	bal	neg	1.0	29.74	16.76	27.68	30.18
$\mathcal{LN}(0,1)$	bal	pos	1.0	23.92	7.24	22.98	24.00
$\mathcal{LN}(0,1)$	unb	hom	1.0	44.60	30.22	42.72	44.38
$\mathcal{LN}(0,1)$	unb	neg	1.0	28.90	18.16	26.54	29.28
$\mathcal{LN}(0,1)$	unb	pos	1.0	22.38	9.46	21.34	27.22
$\mathcal{N}(0,1)$	bal	hom	1.0	34.48	23.22	32.58	39.60
$\mathcal{N}(0,1)$	bal	neg	1.0	18.84	11.92	16.80	22.52
$\mathcal{N}(0,1)$	bal	pos	1.0	16.50	8.90	15.78	21.16
$\mathcal{N}(0,1)$	unb	hom	1.0	29.28	19.86	26.66	36.56
$\mathcal{N}(0,1)$	unb	neg	1.0	14.42	9.20	12.50	19.72
$\mathcal{N}(0,1)$	unb	pos	1.0	16.20	9.62	15.18	23.96
t_2	bal	hom	1.0	17.72	14.58	16.70	27.14
t_2	bal	neg	1.0	9.46	7.34	8.36	15.60
t_2	bal	pos	1.0	8.28	5.94	7.84	16.96
t_2	unb	hom	1.0	9.90	8.08	8.98	20.46
t_2	unb	neg	1.0	4.96	3.68	4.22	11.48
t_2	unb	pos	1.0	5.52	3.90	5.16	15.02
t_3	bal	hom	1.0	23.74	18.40	21.96	31.54
t_3	bal	neg	1.0	11.54	8.48	10.14	17.66
t_3	bal	pos	1.0	11.44	7.12	11.08	18.26
t_3	unb	hom	1.0	15.20	11.64	13.28	25.02
t_3	unb	neg	1.0	7.48	5.44	6.42	13.50
t_3	unb	pos	1.0	8.34	5.96	7.66	19.48

Table 39: Empirical Power for $\delta = 1.0$ in % of two-sided Dunnett-type tests with interval-based estimator

Setting				Method			
Distribution	Design		δ	asymp. MCTP	boot. MCTP	B. asymp.	B. perm.
χ^2_3	bal	hom	1.0	18.90	14.88	17.52	18.12
χ^2_3	bal	neg	1.0	15.28	12.20	13.30	15.90
χ^2_3	bal	pos	1.0	10.00	8.54	9.54	9.26
χ^2_3	unb	hom	1.0	19.06	17.80	17.26	16.30
χ^2_3	unb	neg	1.0	16.86	15.16	14.10	16.12
χ^2_3	unb	pos	1.0	9.82	9.50	9.28	9.04
$\mathcal{LN}(0,1)$	bal	hom	1.0	55.28	42.62	52.92	54.90
$\mathcal{LN}(0,1)$	bal	neg	1.0	36.16	27.12	33.22	36.14
$\mathcal{LN}(0,1)$	bal	pos	1.0	30.30	22.86	29.08	30.74
$\mathcal{LN}(0,1)$	unb	hom	1.0	53.36	45.86	49.88	46.02
$\mathcal{LN}(0,1)$	unb	neg	1.0	35.48	30.82	32.02	30.20
$\mathcal{LN}(0,1)$	unb	pos	1.0	29.96	25.76	28.62	30.12
$\mathcal{N}(0,1)$	bal	hom	1.0	44.78	41.96	41.64	49.78
$\mathcal{N}(0,1)$	bal	neg	1.0	26.30	26.32	22.88	29.86
$\mathcal{N}(0,1)$	bal	pos	1.0	22.64	23.54	21.22	28.34
$\mathcal{N}(0,1)$	unb	hom	1.0	40.12	40.32	35.62	41.78
$\mathcal{N}(0,1)$	unb	neg	1.0	21.62	24.18	17.56	22.56
$\mathcal{N}(0,1)$	unb	pos	1.0	24.08	24.78	21.96	28.46
t_2	bal	hom	1.0	25.76	31.68	23.46	37.04
t_2	bal	neg	1.0	13.58	19.78	11.74	21.36
t_2	bal	pos	1.0	13.34	17.46	12.46	22.70
t_2	unb	hom	1.0	15.24	22.82	13.06	27.12
t_2	unb	neg	1.0	7.74	13.76	6.32	14.06
t_2	unb	pos	1.0	8.58	13.44	7.62	20.62
t_3	bal	hom	1.0	32.84	35.68	29.92	40.76
t_3	bal	neg	1.0	17.16	21.76	14.68	23.36
t_3	bal	pos	1.0	16.06	19.34	15.14	24.60
t_3	unb	hom	1.0	22.50	28.88	19.38	31.40
t_3	unb	neg	1.0	11.54	17.22	9.52	16.86
t_3	unb	pos	1.0	13.36	17.68	12.02	23.70

Table 40: Empirical Power for $\delta = 1.0$ in % of non-inferiority Dunnett-type tests with interval-based estimator

Setting				Method			
Distribution	Design		δ	asymp. MCTP	boot. MCTP	B. asymp.	B. perm.
χ^2_3	bal	hom	1.0	14.04	4.24	12.74	12.98
χ^2_3	bal	neg	1.0	12.16	4.98	11.02	12.26
χ^2_3	bal	pos	1.0	8.30	1.70	8.10	8.10
χ^2_3	unb	hom	1.0	15.90	5.24	14.60	13.60
χ^2_3	unb	neg	1.0	14.32	5.72	12.84	13.44
χ^2_3	unb	pos	1.0	7.70	1.88	7.28	6.68
$\mathcal{LN}(0,1)$	bal	hom	1.0	44.42	24.44	42.32	45.40
$\mathcal{LN}(0,1)$	bal	neg	1.0	28.10	15.88	25.82	29.00
$\mathcal{LN}(0,1)$	bal	pos	1.0	18.66	5.72	18.12	24.32
$\mathcal{LN}(0,1)$	unb	hom	1.0	46.98	28.36	45.12	45.06
$\mathcal{LN}(0,1)$	unb	neg	1.0	31.36	17.56	28.78	30.04
$\mathcal{LN}(0,1)$	unb	pos	1.0	21.72	7.02	20.66	26.06
$\mathcal{N}(0,1)$	bal	hom	1.0	41.22	20.68	39.26	38.98
$\mathcal{N}(0,1)$	bal	neg	1.0	23.46	10.68	21.12	20.70
$\mathcal{N}(0,1)$	bal	pos	1.0	22.36	8.36	21.44	23.34
$\mathcal{N}(0,1)$	unb	hom	1.0	43.38	18.20	40.18	37.34
$\mathcal{N}(0,1)$	unb	neg	1.0	25.92	10.08	22.90	20.04
$\mathcal{N}(0,1)$	unb	pos	1.0	24.22	8.28	23.12	21.74
t_2	bal	hom	1.0	24.28	15.24	22.30	29.46
t_2	bal	neg	1.0	12.94	7.70	11.30	15.42
t_2	bal	pos	1.0	11.80	5.92	11.32	18.86
t_2	unb	hom	1.0	24.44	11.74	22.38	25.46
t_2	unb	neg	1.0	15.18	6.64	13.04	15.10
t_2	unb	pos	1.0	13.12	5.38	12.26	16.32
t_3	bal	hom	1.0	30.40	17.50	28.38	33.86
t_3	bal	neg	1.0	15.44	8.62	13.82	16.90
t_3	bal	pos	1.0	15.44	6.96	14.70	20.20
t_3	unb	hom	1.0	31.48	13.86	28.90	30.22
t_3	unb	neg	1.0	18.68	8.58	16.24	17.10
t_3	unb	pos	1.0	16.90	6.56	15.76	18.38

Table 41: Empirical Power for $\delta = 1.0$ in % of two-sided Dunnett-type tests with kernel estimator

Setting				Method			
Distribution	Design		δ	asymp. MCTP	boot. MCTP	B. asymp.	B. perm.
χ_3^2	bal	hom	1.0	17.94	12.88	16.64	17.68
χ_3^2	bal	neg	1.0	14.78	11.48	13.32	15.22
χ_3^2	bal	pos	1.0	9.52	7.12	8.88	9.94
χ_3^2	unb	hom	1.0	20.22	15.60	18.48	16.84
χ_3^2	unb	neg	1.0	18.56	13.96	16.12	16.00
χ_3^2	unb	pos	1.0	10.06	6.80	9.16	8.02
$\mathcal{LN}(0,1)$	bal	hom	1.0	53.94	42.68	51.22	55.92
$\mathcal{LN}(0,1)$	bal	neg	1.0	34.82	27.52	32.10	35.80
$\mathcal{LN}(0,1)$	bal	pos	1.0	26.38	21.28	25.14	31.08
$\mathcal{LN}(0,1)$	unb	hom	1.0	55.00	46.06	51.84	49.48
$\mathcal{LN}(0,1)$	unb	neg	1.0	37.32	31.28	33.94	32.52
$\mathcal{LN}(0,1)$	unb	pos	1.0	29.80	23.24	28.40	31.02
$\mathcal{N}(0,1)$	bal	hom	1.0	49.90	37.10	47.18	48.86
$\mathcal{N}(0,1)$	bal	neg	1.0	30.18	24.04	26.38	27.46
$\mathcal{N}(0,1)$	bal	pos	1.0	28.60	20.30	27.54	30.50
$\mathcal{N}(0,1)$	unb	hom	1.0	53.50	37.78	49.20	44.86
$\mathcal{N}(0,1)$	unb	neg	1.0	32.62	24.16	28.20	24.40
$\mathcal{N}(0,1)$	unb	pos	1.0	31.66	20.90	29.56	27.48
t_2	bal	hom	1.0	32.94	31.04	30.38	39.06
t_2	bal	neg	1.0	17.98	20.82	15.08	21.16
t_2	bal	pos	1.0	17.86	16.10	16.62	24.92
t_2	unb	hom	1.0	33.76	29.20	29.82	32.66
t_2	unb	neg	1.0	20.14	19.28	16.90	18.86
t_2	unb	pos	1.0	19.04	15.68	17.40	21.34
t_3	bal	hom	1.0	39.78	33.90	36.66	42.68
t_3	bal	neg	1.0	21.74	21.14	18.50	22.92
t_3	bal	pos	1.0	21.26	17.36	20.28	26.76
t_3	unb	hom	1.0	41.08	32.42	36.96	37.42
t_3	unb	neg	1.0	24.24	21.34	21.02	20.78
t_3	unb	pos	1.0	22.86	17.72	20.90	23.34

Table 42: Empirical Power for $\delta = 1.0$ in % of non-inferiority Dunnett-type tests with kernel estimator

Setting				Method			
Distribution	Design		δ	asymp. MCTP	boot. MCTP	B. asymp.	B. perm.
χ^2_3	bal	hom	1.0	13.56	13.82	12.12	14.14
χ^2_3	bal	neg	1.0	13.42	13.30	11.24	14.56
χ^2_3	bal	pos	1.0	7.44	7.52	6.12	8.04
χ^2_3	unb	hom	1.0	19.80	18.98	17.34	19.22
χ^2_3	unb	neg	1.0	18.86	17.82	16.32	19.86
χ^2_3	unb	pos	1.0	8.28	8.40	6.86	8.00
$\mathcal{LN}(0,1)$	bal	hom	1.0	47.12	50.28	43.30	54.24
$\mathcal{LN}(0,1)$	bal	neg	1.0	37.50	39.72	33.48	44.96
$\mathcal{LN}(0,1)$	bal	pos	1.0	17.48	19.30	14.84	24.28
$\mathcal{LN}(0,1)$	unb	hom	1.0	63.54	64.30	60.44	69.18
$\mathcal{LN}(0,1)$	unb	neg	1.0	53.02	52.88	48.78	59.96
$\mathcal{LN}(0,1)$	unb	pos	1.0	23.84	26.78	20.66	31.16
$\mathcal{N}(0,1)$	bal	hom	1.0	52.06	48.30	48.58	50.74
$\mathcal{N}(0,1)$	bal	neg	1.0	41.04	37.68	37.48	39.86
$\mathcal{N}(0,1)$	bal	pos	1.0	23.96	22.82	21.40	24.58
$\mathcal{N}(0,1)$	unb	hom	1.0	61.14	55.92	58.38	59.70
$\mathcal{N}(0,1)$	unb	neg	1.0	50.42	43.90	46.04	48.10
$\mathcal{N}(0,1)$	unb	pos	1.0	28.34	26.44	25.44	27.44
t_2	bal	hom	1.0	34.28	39.64	30.82	39.64
t_2	bal	neg	1.0	24.18	29.10	21.12	29.98
t_2	bal	pos	1.0	14.10	18.84	12.08	19.60
t_2	unb	hom	1.0	40.26	44.08	36.52	44.78
t_2	unb	neg	1.0	30.96	33.74	27.26	34.88
t_2	unb	pos	1.0	16.32	20.26	14.16	21.40
t_3	bal	hom	1.0	40.82	43.40	37.24	44.22
t_3	bal	neg	1.0	29.42	31.68	26.42	32.22
t_3	bal	pos	1.0	17.80	20.12	15.50	20.96
t_3	unb	hom	1.0	47.88	48.22	44.34	50.20
t_3	unb	neg	1.0	37.56	36.78	33.14	38.62
t_3	unb	pos	1.0	20.04	22.50	17.40	23.28

Table 43: Empirical Power for $\delta = 1.0$ in % of two-sided Tukey-type tests with bootstrap estimator

Setting				Method			
Distribution	Design		δ	asymp. MCTP	boot. MCTP	B. asymp.	B. perm.
χ^2_3	bal	hom	1.0	18.76	22.62	16.52	21.16
χ^2_3	bal	neg	1.0	17.98	20.84	15.98	20.88
χ^2_3	bal	pos	1.0	9.64	13.66	8.14	11.26
χ^2_3	unb	hom	1.0	26.34	29.48	24.02	25.20
χ^2_3	unb	neg	1.0	25.36	27.62	22.72	25.80
χ^2_3	unb	pos	1.0	12.14	15.90	10.58	11.12
$\mathcal{LN}(0,1)$	bal	hom	1.0	57.16	62.74	53.46	65.82
$\mathcal{LN}(0,1)$	bal	neg	1.0	46.30	51.68	42.84	56.64
$\mathcal{LN}(0,1)$	bal	pos	1.0	24.12	33.70	21.14	33.52
$\mathcal{LN}(0,1)$	unb	hom	1.0	72.44	75.04	69.36	76.30
$\mathcal{LN}(0,1)$	unb	neg	1.0	62.00	64.50	58.12	67.56
$\mathcal{LN}(0,1)$	unb	pos	1.0	32.20	42.16	28.74	39.00
$\mathcal{N}(0,1)$	bal	hom	1.0	61.26	60.68	57.90	62.34
$\mathcal{N}(0,1)$	bal	neg	1.0	50.36	51.38	47.00	51.62
$\mathcal{N}(0,1)$	bal	pos	1.0	31.56	33.94	28.66	33.38
$\mathcal{N}(0,1)$	unb	hom	1.0	70.68	69.12	67.58	68.62
$\mathcal{N}(0,1)$	unb	neg	1.0	60.62	59.72	56.68	58.52
$\mathcal{N}(0,1)$	unb	pos	1.0	36.38	38.38	33.74	34.80
t_2	bal	hom	1.0	43.74	53.04	40.60	50.84
t_2	bal	neg	1.0	32.68	42.80	29.54	40.72
t_2	bal	pos	1.0	20.06	28.18	17.70	27.68
t_2	unb	hom	1.0	49.50	57.74	46.10	53.94
t_2	unb	neg	1.0	40.38	49.06	36.20	44.50
t_2	unb	pos	1.0	22.92	30.88	20.46	28.36
t_3	bal	hom	1.0	50.84	55.90	47.30	55.74
t_3	bal	neg	1.0	38.36	44.42	34.86	43.40
t_3	bal	pos	1.0	24.04	29.90	21.52	28.90
t_3	unb	hom	1.0	58.06	62.20	54.26	59.76
t_3	unb	neg	1.0	47.32	52.16	43.20	48.86
t_3	unb	pos	1.0	26.98	33.38	24.82	31.12

Table 44: Empirical Power for $\delta = 1.0$ in % of non-inferiority Tukey-type tests with bootstrap estimator

Setting				Method			
Distribution	Design		δ	asymp. MCTP	boot. MCTP	B. asymp.	B. perm.
χ_3^2	bal	hom	1.0	19.92	6.30	17.48	17.00
χ_3^2	bal	neg	1.0	20.16	6.96	17.68	19.28
χ_3^2	bal	pos	1.0	9.58	2.04	8.14	7.54
χ_3^2	unb	hom	1.0	23.66	9.60	21.40	22.06
χ_3^2	unb	neg	1.0	21.88	9.44	19.38	23.04
χ_3^2	unb	pos	1.0	10.02	3.12	8.60	8.96
$\mathcal{LN}(0,1)$	bal	hom	1.0	67.94	38.32	64.36	65.90
$\mathcal{LN}(0,1)$	bal	neg	1.0	57.48	30.04	53.74	57.40
$\mathcal{LN}(0,1)$	bal	pos	1.0	31.08	9.90	27.42	28.20
$\mathcal{LN}(0,1)$	unb	hom	1.0	74.00	50.38	70.56	73.76
$\mathcal{LN}(0,1)$	unb	neg	1.0	63.78	38.56	59.94	65.02
$\mathcal{LN}(0,1)$	unb	pos	1.0	33.26	13.70	29.52	35.52
$\mathcal{N}(0,1)$	bal	hom	1.0	48.58	30.74	45.20	51.48
$\mathcal{N}(0,1)$	bal	neg	1.0	37.96	22.02	34.60	42.62
$\mathcal{N}(0,1)$	bal	pos	1.0	20.56	10.72	18.34	23.14
$\mathcal{N}(0,1)$	unb	hom	1.0	56.64	37.02	52.40	59.40
$\mathcal{N}(0,1)$	unb	neg	1.0	45.42	25.86	41.26	48.14
$\mathcal{N}(0,1)$	unb	pos	1.0	24.02	13.02	21.24	28.38
t_2	bal	hom	1.0	27.26	19.54	24.52	36.06
t_2	bal	neg	1.0	18.98	12.38	16.52	29.20
t_2	bal	pos	1.0	10.22	6.54	8.88	17.74
t_2	unb	hom	1.0	30.64	21.62	26.66	39.74
t_2	unb	neg	1.0	24.38	15.40	20.62	31.32
t_2	unb	pos	1.0	11.34	7.60	9.40	19.24
t_3	bal	hom	1.0	33.58	23.30	30.42	41.72
t_3	bal	neg	1.0	24.18	15.24	21.08	32.76
t_3	bal	pos	1.0	13.94	7.80	11.86	18.84
t_3	unb	hom	1.0	39.32	26.34	35.44	46.74
t_3	unb	neg	1.0	29.84	18.84	26.22	35.70
t_3	unb	pos	1.0	14.90	9.22	13.02	22.58

Table 45: Empirical Power for $\delta = 1.0$ in % of two-sided Tukey-type tests with interval-based estimator

Setting				Method			
Distribution	Design		δ	asymp. MCTP	boot. MCTP	B. asymp.	B. perm.
χ^2_3	bal	hom	1.0	26.46	15.30	23.94	24.46
χ^2_3	bal	neg	1.0	25.62	14.12	23.28	26.16
χ^2_3	bal	pos	1.0	13.30	7.38	11.16	10.78
χ^2_3	unb	hom	1.0	31.40	18.86	28.42	27.12
χ^2_3	unb	neg	1.0	29.46	16.52	26.14	28.44
χ^2_3	unb	pos	1.0	13.76	8.24	12.06	11.28
$\mathcal{LN}(0,1)$	bal	hom	1.0	76.02	54.30	73.28	76.30
$\mathcal{LN}(0,1)$	bal	neg	1.0	67.00	42.02	63.44	68.56
$\mathcal{LN}(0,1)$	bal	pos	1.0	39.64	24.44	36.02	37.98
$\mathcal{LN}(0,1)$	unb	hom	1.0	81.28	63.28	78.84	80.04
$\mathcal{LN}(0,1)$	unb	neg	1.0	71.96	50.90	68.48	71.42
$\mathcal{LN}(0,1)$	unb	pos	1.0	42.46	29.30	39.16	42.12
$\mathcal{N}(0,1)$	bal	hom	1.0	59.46	47.00	55.66	64.58
$\mathcal{N}(0,1)$	bal	neg	1.0	48.02	35.72	44.30	55.16
$\mathcal{N}(0,1)$	bal	pos	1.0	27.66	22.98	25.16	31.12
$\mathcal{N}(0,1)$	unb	hom	1.0	66.24	50.84	62.92	67.90
$\mathcal{N}(0,1)$	unb	neg	1.0	55.24	39.26	51.26	57.94
$\mathcal{N}(0,1)$	unb	pos	1.0	32.72	23.40	30.02	35.28
t_2	bal	hom	1.0	35.98	33.06	32.92	47.34
t_2	bal	neg	1.0	26.00	24.18	23.08	39.70
t_2	bal	pos	1.0	14.56	15.30	12.74	24.24
t_2	unb	hom	1.0	40.14	34.52	36.12	50.70
t_2	unb	neg	1.0	32.30	27.48	28.20	41.20
t_2	unb	pos	1.0	16.12	15.32	14.00	27.80
t_3	bal	hom	1.0	43.12	38.06	39.82	54.22
t_3	bal	neg	1.0	32.50	26.90	29.04	44.14
t_3	bal	pos	1.0	19.06	17.66	17.06	26.28
t_3	unb	hom	1.0	49.60	40.18	45.12	56.98
t_3	unb	neg	1.0	38.92	31.16	34.68	45.80
t_3	unb	pos	1.0	20.58	17.84	18.26	30.38

Table 46: Empirical Power for $\delta = 1.0$ in % of non-inferiority Tukey-type tests with interval-based estimator

Setting				Method			
Distribution	Design		δ	asymp. MCTP	boot. MCTP	B. asymp.	B. perm.
χ_3^2	bal	hom	1.0	18.50	4.98	16.20	15.68
χ_3^2	bal	neg	1.0	19.28	5.14	17.24	17.90
χ_3^2	bal	pos	1.0	9.68	2.12	8.04	7.68
χ_3^2	unb	hom	1.0	25.10	6.24	22.16	20.76
χ_3^2	unb	neg	1.0	24.42	6.34	21.76	23.44
χ_3^2	unb	pos	1.0	10.16	1.90	8.42	7.48
$\mathcal{LN}(0,1)$	bal	hom	1.0	66.04	33.50	61.70	66.66
$\mathcal{LN}(0,1)$	bal	neg	1.0	55.64	26.06	51.64	56.66
$\mathcal{LN}(0,1)$	bal	pos	1.0	24.96	7.62	21.56	28.44
$\mathcal{LN}(0,1)$	unb	hom	1.0	76.84	43.84	74.22	78.58
$\mathcal{LN}(0,1)$	unb	neg	1.0	65.48	32.34	61.50	69.28
$\mathcal{LN}(0,1)$	unb	pos	1.0	31.00	9.18	27.12	36.12
$\mathcal{N}(0,1)$	bal	hom	1.0	55.74	26.06	52.18	51.16
$\mathcal{N}(0,1)$	bal	neg	1.0	43.62	17.30	40.42	38.56
$\mathcal{N}(0,1)$	bal	pos	1.0	27.06	9.62	24.40	24.94
$\mathcal{N}(0,1)$	unb	hom	1.0	64.90	27.38	61.46	59.62
$\mathcal{N}(0,1)$	unb	neg	1.0	53.94	16.80	49.92	48.12
$\mathcal{N}(0,1)$	unb	pos	1.0	31.12	9.54	27.92	26.94
t_2	bal	hom	1.0	35.48	19.68	32.22	39.44
t_2	bal	neg	1.0	24.74	12.30	21.96	29.26
t_2	bal	pos	1.0	14.54	6.52	12.30	19.60
t_2	unb	hom	1.0	41.90	18.50	38.12	44.76
t_2	unb	neg	1.0	33.56	11.20	28.90	35.62
t_2	unb	pos	1.0	17.12	6.56	14.88	20.18
t_3	bal	hom	1.0	42.32	22.10	38.78	44.26
t_3	bal	neg	1.0	30.84	14.36	27.48	31.58
t_3	bal	pos	1.0	18.58	7.26	16.00	21.00
t_3	unb	hom	1.0	50.10	21.22	45.94	49.88
t_3	unb	neg	1.0	40.04	13.28	36.16	40.36
t_3	unb	pos	1.0	21.30	7.22	18.78	22.68

Table 47: Empirical Power for $\delta = 1.0$ in % of two-sided Tukey-type tests with kernel estimator

Setting				Method			
Distribution	Design		δ	asymp. MCTP	boot. MCTP	B. asymp.	B. perm.
χ_3^2	bal	hom	1.0	25.60	12.14	22.46	23.08
χ_3^2	bal	neg	1.0	25.20	11.46	22.76	25.34
χ_3^2	bal	pos	1.0	12.46	5.88	10.84	10.80
χ_3^2	unb	hom	1.0	32.22	16.74	29.48	27.94
χ_3^2	unb	neg	1.0	31.38	15.32	28.36	30.30
χ_3^2	unb	pos	1.0	14.16	6.56	12.14	10.64
$\mathcal{LN}(0,1)$	bal	hom	1.0	74.78	51.76	71.96	77.42
$\mathcal{LN}(0,1)$	bal	neg	1.0	65.22	39.62	61.88	68.84
$\mathcal{LN}(0,1)$	bal	pos	1.0	33.92	20.26	30.40	38.98
$\mathcal{LN}(0,1)$	unb	hom	1.0	83.96	63.98	81.40	84.78
$\mathcal{LN}(0,1)$	unb	neg	1.0	73.68	52.08	70.18	76.58
$\mathcal{LN}(0,1)$	unb	pos	1.0	40.96	27.36	37.46	46.36
$\mathcal{N}(0,1)$	bal	hom	1.0	64.44	41.08	62.08	62.68
$\mathcal{N}(0,1)$	bal	neg	1.0	54.08	30.96	50.60	51.84
$\mathcal{N}(0,1)$	bal	pos	1.0	34.06	18.66	31.36	33.00
$\mathcal{N}(0,1)$	unb	hom	1.0	74.26	45.94	71.22	69.84
$\mathcal{N}(0,1)$	unb	neg	1.0	63.84	36.04	59.40	59.12
$\mathcal{N}(0,1)$	unb	pos	1.0	39.00	19.98	36.34	35.50
t_2	bal	hom	1.0	44.64	33.78	41.18	50.26
t_2	bal	neg	1.0	34.00	24.58	30.38	40.10
t_2	bal	pos	1.0	20.14	14.58	17.76	27.26
t_2	unb	hom	1.0	51.96	34.58	48.68	55.02
t_2	unb	neg	1.0	42.92	26.78	39.26	45.98
t_2	unb	pos	1.0	23.52	14.12	21.20	27.28
t_3	bal	hom	1.0	52.70	36.50	48.98	55.90
t_3	bal	neg	1.0	39.44	25.48	35.92	42.98
t_3	bal	pos	1.0	24.86	16.04	22.44	28.64
t_3	unb	hom	1.0	60.62	38.72	57.14	60.42
t_3	unb	neg	1.0	50.10	29.22	45.58	50.24
t_3	unb	pos	1.0	28.28	16.16	25.86	30.08

Table 48: Empirical Power for $\delta = 1.0$ in % of non-inferiority Tukey-type tests with kernel estimator

Setting				Method			
Distribution	Design		δ	asymp. MCTP	boot. MCTP	B. asymp.	B. perm.
χ^2_3	bal	hom	1.0	12.40	14.02	11.62	14.56
χ^2_3	bal	neg	1.0	10.34	12.28	9.50	12.74
χ^2_3	bal	pos	1.0	7.42	8.76	6.78	8.74
χ^2_3	unb	hom	1.0	16.22	18.22	15.16	19.08
χ^2_3	unb	neg	1.0	12.52	14.60	11.80	16.66
χ^2_3	unb	pos	1.0	7.40	8.72	6.74	9.46
$\mathcal{LN}(0,1)$	bal	hom	1.0	41.74	49.62	39.78	51.32
$\mathcal{LN}(0,1)$	bal	neg	1.0	27.38	35.16	25.78	36.88
$\mathcal{LN}(0,1)$	bal	pos	1.0	15.60	21.30	14.36	24.14
$\mathcal{LN}(0,1)$	unb	hom	1.0	55.28	61.54	53.14	69.74
$\mathcal{LN}(0,1)$	unb	neg	1.0	36.52	44.34	34.36	50.88
$\mathcal{LN}(0,1)$	unb	pos	1.0	20.48	26.62	19.18	32.52
$\mathcal{N}(0,1)$	bal	hom	1.0	52.24	51.12	50.80	54.86
$\mathcal{N}(0,1)$	bal	neg	1.0	39.86	39.80	38.28	42.76
$\mathcal{N}(0,1)$	bal	pos	1.0	23.82	24.08	22.64	27.06
$\mathcal{N}(0,1)$	unb	hom	1.0	61.48	58.78	59.80	66.04
$\mathcal{N}(0,1)$	unb	neg	1.0	46.64	44.58	44.88	50.52
$\mathcal{N}(0,1)$	unb	pos	1.0	28.06	27.74	26.76	31.84
t_2	bal	hom	1.0	34.94	43.10	33.74	43.26
t_2	bal	neg	1.0	22.54	31.26	21.06	31.52
t_2	bal	pos	1.0	13.70	20.66	12.92	21.42
t_2	unb	hom	1.0	38.42	47.30	36.70	50.46
t_2	unb	neg	1.0	24.40	33.40	22.40	34.08
t_2	unb	pos	1.0	15.54	22.24	14.52	25.52
t_3	bal	hom	1.0	41.86	46.38	40.50	48.14
t_3	bal	neg	1.0	29.18	34.26	27.86	35.10
t_3	bal	pos	1.0	16.92	21.52	15.94	22.78
t_3	unb	hom	1.0	47.62	51.72	45.68	56.12
t_3	unb	neg	1.0	32.08	37.38	30.12	39.74
t_3	unb	pos	1.0	19.82	24.58	18.72	27.44

Table 49: Empirical Power for $\delta = 1.0$ in % of two-sided Grand-mean-type tests with bootstrap estimator

Setting				Method			
Distribution	Design		δ	asymp. MCTP	boot. MCTP	B. asymp.	B. perm.
χ_3^2	bal	hom	1.0	11.10	15.58	11.04	17.40
χ_3^2	bal	neg	1.0	9.26	13.18	9.22	14.94
χ_3^2	bal	pos	1.0	5.82	8.58	5.78	9.54
χ_3^2	unb	hom	1.0	15.80	20.48	15.68	21.54
χ_3^2	unb	neg	1.0	12.66	17.50	12.46	18.54
χ_3^2	unb	pos	1.0	6.52	9.58	6.50	10.34
$\mathcal{LN}(0,1)$	bal	hom	1.0	45.88	57.38	45.68	65.86
$\mathcal{LN}(0,1)$	bal	neg	1.0	29.82	42.56	29.60	51.06
$\mathcal{LN}(0,1)$	bal	pos	1.0	15.16	24.72	14.88	29.50
$\mathcal{LN}(0,1)$	unb	hom	1.0	59.74	69.02	59.48	74.28
$\mathcal{LN}(0,1)$	unb	neg	1.0	40.86	52.14	40.48	55.68
$\mathcal{LN}(0,1)$	unb	pos	1.0	20.52	31.88	20.44	36.86
$\mathcal{N}(0,1)$	bal	hom	1.0	56.84	57.60	56.70	61.96
$\mathcal{N}(0,1)$	bal	neg	1.0	44.84	46.78	44.64	49.80
$\mathcal{N}(0,1)$	bal	pos	1.0	25.34	26.66	25.18	30.32
$\mathcal{N}(0,1)$	unb	hom	1.0	67.80	66.64	67.60	70.06
$\mathcal{N}(0,1)$	unb	neg	1.0	52.96	52.56	52.66	54.62
$\mathcal{N}(0,1)$	unb	pos	1.0	30.84	31.60	30.76	34.12
t_2	bal	hom	1.0	39.50	48.28	39.36	50.10
t_2	bal	neg	1.0	26.68	37.22	26.54	37.06
t_2	bal	pos	1.0	15.68	23.12	15.60	25.08
t_2	unb	hom	1.0	45.52	54.18	45.28	54.68
t_2	unb	neg	1.0	29.76	39.98	29.48	38.30
t_2	unb	pos	1.0	18.74	25.50	18.70	27.24
t_3	bal	hom	1.0	46.38	52.42	46.38	54.86
t_3	bal	neg	1.0	34.34	40.42	34.24	41.92
t_3	bal	pos	1.0	18.44	23.50	18.40	26.08
t_3	unb	hom	1.0	53.90	58.60	53.58	60.24
t_3	unb	neg	1.0	38.74	44.56	38.36	43.94
t_3	unb	pos	1.0	23.18	27.36	23.06	29.16

Table 50: Empirical Power for $\delta = 1.0$ in % of non-inferiority Grand-mean-type tests with bootstrap estimator

Setting				Method			
Distribution	Design		δ	asymp. MCTP	boot. MCTP	B. asymp.	B. perm.
χ_3^2	bal	hom	1.0	19.02	6.84	17.92	15.66
χ_3^2	bal	neg	1.0	16.90	6.24	15.84	16.16
χ_3^2	bal	pos	1.0	11.20	3.40	10.38	8.10
χ_3^2	unb	hom	1.0	19.52	8.84	18.52	21.12
χ_3^2	unb	neg	1.0	16.00	6.72	14.54	20.92
χ_3^2	unb	pos	1.0	9.68	3.88	9.10	10.52
$\mathcal{LN}(0,1)$	bal	hom	1.0	63.44	36.52	61.56	59.14
$\mathcal{LN}(0,1)$	bal	neg	1.0	48.00	23.98	46.60	46.10
$\mathcal{LN}(0,1)$	bal	pos	1.0	30.76	11.86	29.00	26.42
$\mathcal{LN}(0,1)$	unb	hom	1.0	66.32	45.56	64.38	74.74
$\mathcal{LN}(0,1)$	unb	neg	1.0	48.44	30.00	45.84	57.32
$\mathcal{LN}(0,1)$	unb	pos	1.0	28.44	14.24	27.02	36.26
$\mathcal{N}(0,1)$	bal	hom	1.0	50.00	35.00	48.38	54.60
$\mathcal{N}(0,1)$	bal	neg	1.0	37.46	24.54	35.72	45.30
$\mathcal{N}(0,1)$	bal	pos	1.0	20.58	12.36	19.54	25.00
$\mathcal{N}(0,1)$	unb	hom	1.0	55.52	40.74	53.60	66.82
$\mathcal{N}(0,1)$	unb	neg	1.0	37.70	25.04	35.58	49.68
$\mathcal{N}(0,1)$	unb	pos	1.0	23.70	14.18	22.34	33.48
t_2	bal	hom	1.0	27.16	24.54	25.72	40.34
t_2	bal	neg	1.0	16.56	14.62	15.58	29.70
t_2	bal	pos	1.0	9.84	9.00	9.18	19.96
t_2	unb	hom	1.0	19.84	18.32	17.98	41.84
t_2	unb	neg	1.0	9.58	7.78	8.62	25.66
t_2	unb	pos	1.0	7.84	7.46	7.30	24.10
t_3	bal	hom	1.0	34.24	28.08	32.76	45.38
t_3	bal	neg	1.0	22.66	17.52	21.52	35.46
t_3	bal	pos	1.0	12.98	9.74	12.20	20.52
t_3	unb	hom	1.0	31.06	25.78	28.88	51.00
t_3	unb	neg	1.0	16.40	12.28	14.66	33.14
t_3	unb	pos	1.0	12.78	9.90	11.96	27.38

Table 51: Empirical Power for $\delta = 1.0$ in % of two-sided Grand-mean-type tests with interval-based estimator

Setting				Method			
Distribution	Design		δ	asymp. MCTP	boot. MCTP	B. asymp.	B. perm.
χ_3^2	bal	hom	1.0	15.02	8.30	14.96	20.12
χ_3^2	bal	neg	1.0	12.80	7.14	12.70	19.26
χ_3^2	bal	pos	1.0	6.78	3.04	6.72	9.78
χ_3^2	unb	hom	1.0	18.26	11.60	18.18	22.88
χ_3^2	unb	neg	1.0	15.58	9.68	15.42	22.04
χ_3^2	unb	pos	1.0	7.62	4.44	7.62	10.54
$\mathcal{LN}(0,1)$	bal	hom	1.0	64.16	49.24	64.02	76.90
$\mathcal{LN}(0,1)$	bal	neg	1.0	47.34	33.00	47.12	62.68
$\mathcal{LN}(0,1)$	bal	pos	1.0	25.72	15.34	25.56	33.30
$\mathcal{LN}(0,1)$	unb	hom	1.0	68.80	58.00	68.60	79.32
$\mathcal{LN}(0,1)$	unb	neg	1.0	50.76	39.18	50.34	62.10
$\mathcal{LN}(0,1)$	unb	pos	1.0	27.52	19.42	27.38	39.70
$\mathcal{N}(0,1)$	bal	hom	1.0	54.52	47.62	54.48	65.20
$\mathcal{N}(0,1)$	bal	neg	1.0	42.34	34.80	42.14	55.26
$\mathcal{N}(0,1)$	bal	pos	1.0	21.80	17.44	21.72	30.44
$\mathcal{N}(0,1)$	unb	hom	1.0	63.06	55.84	62.92	71.24
$\mathcal{N}(0,1)$	unb	neg	1.0	45.00	37.68	44.58	55.36
$\mathcal{N}(0,1)$	unb	pos	1.0	27.72	22.04	27.68	35.76
t_2	bal	hom	1.0	32.26	33.88	32.14	47.80
t_2	bal	neg	1.0	20.54	21.82	20.38	36.50
t_2	bal	pos	1.0	11.48	13.20	11.44	22.60
t_2	unb	hom	1.0	26.14	28.98	25.70	47.32
t_2	unb	neg	1.0	12.48	14.56	12.14	30.04
t_2	unb	pos	1.0	10.36	11.74	10.24	26.96
t_3	bal	hom	1.0	39.80	39.02	39.76	53.62
t_3	bal	neg	1.0	27.88	26.34	27.80	43.42
t_3	bal	pos	1.0	13.64	13.60	13.64	24.06
t_3	unb	hom	1.0	39.14	38.50	38.58	56.74
t_3	unb	neg	1.0	22.42	21.70	21.94	37.48
t_3	unb	pos	1.0	15.62	16.12	15.50	30.08

Table 52: Empirical Power for $\delta = 1.0$ in % of non-inferiority Grand-mean-type tests with interval-based estimator

Setting				Method			
Distribution	Design		δ	asymp. MCTP	boot. MCTP	B. asymp.	B. perm.
χ^2_3	bal	hom	1.0	17.96	5.12	17.04	14.86
χ^2_3	bal	neg	1.0	16.16	4.20	15.32	15.36
χ^2_3	bal	pos	1.0	10.70	2.68	9.86	8.44
χ^2_3	unb	hom	1.0	20.74	6.30	19.66	19.98
χ^2_3	unb	neg	1.0	17.82	5.08	16.68	19.80
χ^2_3	unb	pos	1.0	10.24	2.64	9.42	9.26
$\mathcal{LN}(0,1)$	bal	hom	1.0	60.54	31.00	58.92	57.82
$\mathcal{LN}(0,1)$	bal	neg	1.0	45.04	20.72	42.98	44.74
$\mathcal{LN}(0,1)$	bal	pos	1.0	25.80	8.86	23.74	26.40
$\mathcal{LN}(0,1)$	unb	hom	1.0	68.42	40.54	66.66	77.28
$\mathcal{LN}(0,1)$	unb	neg	1.0	49.80	26.64	47.56	59.06
$\mathcal{LN}(0,1)$	unb	pos	1.0	27.00	11.20	25.64	35.90
$\mathcal{N}(0,1)$	bal	hom	1.0	56.64	29.98	55.40	54.94
$\mathcal{N}(0,1)$	bal	neg	1.0	44.00	20.44	42.40	43.06
$\mathcal{N}(0,1)$	bal	pos	1.0	26.82	10.94	25.50	26.90
$\mathcal{N}(0,1)$	unb	hom	1.0	66.20	32.22	64.34	66.98
$\mathcal{N}(0,1)$	unb	neg	1.0	51.72	20.16	49.90	51.08
$\mathcal{N}(0,1)$	unb	pos	1.0	30.76	10.72	29.84	31.66
t_2	bal	hom	1.0	36.54	23.38	34.92	43.20
t_2	bal	neg	1.0	23.74	14.18	22.72	30.96
t_2	bal	pos	1.0	14.02	8.24	13.10	21.56
t_2	unb	hom	1.0	42.18	21.90	40.24	51.28
t_2	unb	neg	1.0	29.72	12.96	27.80	36.90
t_2	unb	pos	1.0	16.76	7.98	15.84	24.12
t_3	bal	hom	1.0	43.44	25.20	42.04	47.76
t_3	bal	neg	1.0	31.26	16.38	29.60	34.64
t_3	bal	pos	1.0	18.16	8.58	17.08	22.30
t_3	unb	hom	1.0	51.12	24.80	49.44	57.24
t_3	unb	neg	1.0	36.94	14.90	35.04	41.22
t_3	unb	pos	1.0	21.36	8.58	20.30	26.66

Table 53: Empirical Power for $\delta = 1.0$ in % of two-sided Grand-mean-type tests with kernel estimator

Setting				Method			
Distribution	Design		δ	asymp. MCTP	boot. MCTP	B. asymp.	B. perm.
χ_3^2	bal	hom	1.0	14.02	6.82	13.96	18.68
χ_3^2	bal	neg	1.0	12.42	5.80	12.42	17.14
χ_3^2	bal	pos	1.0	6.84	2.98	6.70	9.50
χ_3^2	unb	hom	1.0	18.84	8.20	18.78	23.06
χ_3^2	unb	neg	1.0	17.46	6.76	17.40	23.22
χ_3^2	unb	pos	1.0	7.76	2.70	7.68	9.76
$\mathcal{LN}(0,1)$	bal	hom	1.0	62.40	45.92	62.34	77.36
$\mathcal{LN}(0,1)$	bal	neg	1.0	45.30	32.04	45.16	62.76
$\mathcal{LN}(0,1)$	bal	pos	1.0	20.60	12.90	20.40	34.28
$\mathcal{LN}(0,1)$	unb	hom	1.0	71.42	52.04	71.26	83.02
$\mathcal{LN}(0,1)$	unb	neg	1.0	52.22	34.90	52.00	66.20
$\mathcal{LN}(0,1)$	unb	pos	1.0	25.74	15.78	25.60	42.00
$\mathcal{N}(0,1)$	bal	hom	1.0	60.76	39.54	60.72	62.58
$\mathcal{N}(0,1)$	bal	neg	1.0	49.22	28.54	49.18	50.10
$\mathcal{N}(0,1)$	bal	pos	1.0	27.64	15.02	27.56	30.68
$\mathcal{N}(0,1)$	unb	hom	1.0	71.60	40.18	71.48	71.48
$\mathcal{N}(0,1)$	unb	neg	1.0	58.02	26.60	57.66	55.82
$\mathcal{N}(0,1)$	unb	pos	1.0	33.06	13.80	33.04	34.44
t_2	bal	hom	1.0	40.72	31.70	40.64	50.28
t_2	bal	neg	1.0	27.92	20.66	27.80	37.78
t_2	bal	pos	1.0	15.90	11.12	15.76	24.62
t_2	unb	hom	1.0	49.00	29.04	48.82	55.46
t_2	unb	neg	1.0	35.62	18.02	35.34	41.46
t_2	unb	pos	1.0	18.88	10.90	18.82	26.26
t_3	bal	hom	1.0	48.62	35.12	48.56	55.00
t_3	bal	neg	1.0	36.28	23.76	36.22	42.14
t_3	bal	pos	1.0	18.86	11.88	18.80	25.76
t_3	unb	hom	1.0	56.76	32.68	56.58	61.32
t_3	unb	neg	1.0	42.90	21.20	42.54	45.76
t_3	unb	pos	1.0	23.38	11.76	23.32	28.54

Table 54: Empirical Power for $\delta = 1.0$ in % of non-inferiority Grand-mean-type tests with kernel estimator

Setting				Method			
Distribution	Design		δ	asymp. MCTP	boot. MCTP	B. asymp.	B. perm.
χ^2_3	bal	hom	1.5	18.50	20.46	17.30	22.48
χ^2_3	bal	neg	1.5	12.78	15.04	11.38	14.96
χ^2_3	bal	pos	1.5	9.30	9.80	9.00	11.74
χ^2_3	unb	hom	1.5	23.70	25.52	21.86	25.04
χ^2_3	unb	neg	1.5	16.12	18.52	14.36	17.44
χ^2_3	unb	pos	1.5	10.84	11.68	10.42	12.78
$\mathcal{LN}(0,1)$	bal	hom	1.5	61.90	66.62	59.50	62.22
$\mathcal{LN}(0,1)$	bal	neg	1.5	38.28	43.52	35.68	39.14
$\mathcal{LN}(0,1)$	bal	pos	1.5	31.02	34.26	29.96	43.36
$\mathcal{LN}(0,1)$	unb	hom	1.5	65.40	69.26	62.78	61.84
$\mathcal{LN}(0,1)$	unb	neg	1.5	40.60	45.48	37.84	37.88
$\mathcal{LN}(0,1)$	unb	pos	1.5	40.26	45.20	38.82	48.66
$\mathcal{N}(0,1)$	bal	hom	1.5	75.00	72.64	72.68	76.50
$\mathcal{N}(0,1)$	bal	neg	1.5	44.90	45.00	41.46	44.84
$\mathcal{N}(0,1)$	bal	pos	1.5	43.34	41.84	42.46	48.44
$\mathcal{N}(0,1)$	unb	hom	1.5	72.46	70.30	69.30	71.24
$\mathcal{N}(0,1)$	unb	neg	1.5	43.18	42.78	39.00	40.12
$\mathcal{N}(0,1)$	unb	pos	1.5	45.74	44.18	44.14	47.62
t_2	bal	hom	1.5	52.34	59.62	50.44	59.56
t_2	bal	neg	1.5	28.82	36.74	26.38	33.52
t_2	bal	pos	1.5	26.42	33.02	25.66	36.84
t_2	unb	hom	1.5	47.16	54.28	44.26	52.32
t_2	unb	neg	1.5	23.20	29.22	20.22	26.18
t_2	unb	pos	1.5	27.38	33.66	25.76	36.62
t_3	bal	hom	1.5	60.84	64.04	58.96	65.08
t_3	bal	neg	1.5	33.74	38.82	30.90	36.14
t_3	bal	pos	1.5	32.66	36.02	31.48	40.62
t_3	unb	hom	1.5	56.94	59.74	53.74	59.26
t_3	unb	neg	1.5	31.16	36.12	27.92	31.86
t_3	unb	pos	1.5	32.78	36.70	31.18	39.62

Table 55: Empirical Power for $\delta = 1.5$ in % of two-sided Dunnett-type tests with bootstrap estimator

Setting				Method			
Distribution	Design		δ	asymp. MCTP	boot. MCTP	B. asymp.	B. perm.
χ_3^2	bal	hom	1.5	25.36	32.02	23.06	29.30
χ_3^2	bal	neg	1.5	16.98	23.52	15.24	19.32
χ_3^2	bal	pos	1.5	12.92	18.28	12.08	16.42
χ_3^2	unb	hom	1.5	30.54	37.88	27.94	29.48
χ_3^2	unb	neg	1.5	21.76	28.36	18.90	20.20
χ_3^2	unb	pos	1.5	14.88	21.34	13.80	15.18
$\mathcal{LN}(0,1)$	bal	hom	1.5	71.68	76.12	68.28	72.44
$\mathcal{LN}(0,1)$	bal	neg	1.5	47.20	54.46	43.44	48.80
$\mathcal{LN}(0,1)$	bal	pos	1.5	41.24	52.06	39.66	53.72
$\mathcal{LN}(0,1)$	unb	hom	1.5	73.60	78.32	70.04	65.12
$\mathcal{LN}(0,1)$	unb	neg	1.5	49.16	56.96	44.34	40.16
$\mathcal{LN}(0,1)$	unb	pos	1.5	50.50	60.14	48.10	53.26
$\mathcal{N}(0,1)$	bal	hom	1.5	82.58	82.00	80.56	83.48
$\mathcal{N}(0,1)$	bal	neg	1.5	55.50	59.34	50.64	55.04
$\mathcal{N}(0,1)$	bal	pos	1.5	53.32	56.50	51.50	58.76
$\mathcal{N}(0,1)$	unb	hom	1.5	81.58	79.90	77.60	76.56
$\mathcal{N}(0,1)$	unb	neg	1.5	53.84	57.96	48.10	46.60
$\mathcal{N}(0,1)$	unb	pos	1.5	55.28	58.00	52.72	54.28
t_2	bal	hom	1.5	64.10	71.68	60.80	69.64
t_2	bal	neg	1.5	38.28	50.76	33.90	42.04
t_2	bal	pos	1.5	35.80	46.40	34.18	45.84
t_2	unb	hom	1.5	59.52	69.16	54.16	61.48
t_2	unb	neg	1.5	32.36	45.98	27.12	32.52
t_2	unb	pos	1.5	36.72	48.10	34.54	43.62
t_3	bal	hom	1.5	70.78	74.54	67.98	74.04
t_3	bal	neg	1.5	43.98	53.12	38.96	45.46
t_3	bal	pos	1.5	42.28	49.32	40.60	50.42
t_3	unb	hom	1.5	67.70	72.56	62.92	65.98
t_3	unb	neg	1.5	41.16	51.90	35.88	37.92
t_3	unb	pos	1.5	42.42	50.86	40.12	46.64

Table 56: Empirical Power for $\delta = 1.5$ in % of non-inferiority Dunnett-type tests with bootstrap estimator

Setting				Method			
Distribution	Design		δ	asymp. MCTP	boot. MCTP	B. asymp.	B. perm.
χ^2_3	bal	hom	1.5	25.96	11.60	24.30	25.44
χ^2_3	bal	neg	1.5	18.14	8.76	16.68	18.72
χ^2_3	bal	pos	1.5	12.52	3.30	12.12	11.40
χ^2_3	unb	hom	1.5	27.08	14.96	25.12	27.90
χ^2_3	unb	neg	1.5	18.04	10.10	16.26	20.10
χ^2_3	unb	pos	1.5	12.30	4.70	11.88	13.96
$\mathcal{LN}(0,1)$	bal	hom	1.5	77.64	58.20	75.36	73.42
$\mathcal{LN}(0,1)$	bal	neg	1.5	52.78	35.36	49.80	49.38
$\mathcal{LN}(0,1)$	bal	pos	1.5	48.70	22.48	47.44	50.58
$\mathcal{LN}(0,1)$	unb	hom	1.5	73.22	58.76	70.54	67.62
$\mathcal{LN}(0,1)$	unb	neg	1.5	47.50	34.40	44.30	43.36
$\mathcal{LN}(0,1)$	unb	pos	1.5	48.74	30.38	46.86	52.58
$\mathcal{N}(0,1)$	bal	hom	1.5	72.70	59.88	70.70	76.78
$\mathcal{N}(0,1)$	bal	neg	1.5	42.56	31.22	39.36	46.76
$\mathcal{N}(0,1)$	bal	pos	1.5	38.94	25.08	37.86	46.38
$\mathcal{N}(0,1)$	unb	hom	1.5	62.70	51.02	58.62	69.32
$\mathcal{N}(0,1)$	unb	neg	1.5	31.56	21.26	27.56	37.12
$\mathcal{N}(0,1)$	unb	pos	1.5	37.68	27.40	36.08	49.08
t_2	bal	hom	1.5	44.10	37.84	41.80	55.32
t_2	bal	neg	1.5	22.40	19.16	20.36	31.34
t_2	bal	pos	1.5	19.68	15.20	19.16	31.82
t_2	unb	hom	1.5	26.80	23.16	24.48	40.42
t_2	unb	neg	1.5	10.60	8.44	9.12	19.28
t_2	unb	pos	1.5	14.90	12.38	14.02	31.24
t_3	bal	hom	1.5	53.94	46.38	51.82	62.34
t_3	bal	neg	1.5	27.96	22.42	25.42	35.78
t_3	bal	pos	1.5	26.66	18.50	25.68	37.02
t_3	unb	hom	1.5	38.90	32.68	35.78	50.26
t_3	unb	neg	1.5	16.06	12.32	14.14	23.52
t_3	unb	pos	1.5	20.92	16.08	19.58	36.38

Table 57: Empirical Power for $\delta = 1.5$ in % of two-sided Dunnett-type tests with interval-based estimator

Setting				Method			
Distribution	Design		δ	asymp. MCTP	boot. MCTP	B. asymp.	B. perm.
χ^2_3	bal	hom	1.5	32.78	25.72	30.70	32.92
χ^2_3	bal	neg	1.5	22.40	18.56	20.44	23.02
χ^2_3	bal	pos	1.5	16.00	12.74	15.40	15.40
χ^2_3	unb	hom	1.5	35.12	30.08	32.18	30.48
χ^2_3	unb	neg	1.5	23.54	21.96	20.84	20.96
χ^2_3	unb	pos	1.5	17.10	15.78	16.02	15.50
$\mathcal{LN}(0,1)$	bal	hom	1.5	83.52	70.20	81.10	79.50
$\mathcal{LN}(0,1)$	bal	neg	1.5	59.26	47.44	55.92	56.50
$\mathcal{LN}(0,1)$	bal	pos	1.5	57.76	44.24	56.22	59.66
$\mathcal{LN}(0,1)$	unb	hom	1.5	79.28	71.08	75.78	68.54
$\mathcal{LN}(0,1)$	unb	neg	1.5	55.20	48.08	50.24	44.22
$\mathcal{LN}(0,1)$	unb	pos	1.5	58.14	49.86	55.80	55.80
$\mathcal{N}(0,1)$	bal	hom	1.5	81.16	74.46	78.38	83.54
$\mathcal{N}(0,1)$	bal	neg	1.5	52.52	49.24	47.54	56.64
$\mathcal{N}(0,1)$	bal	pos	1.5	49.60	46.36	47.64	56.86
$\mathcal{N}(0,1)$	unb	hom	1.5	74.38	67.68	68.98	74.00
$\mathcal{N}(0,1)$	unb	neg	1.5	42.48	41.30	36.24	42.00
$\mathcal{N}(0,1)$	unb	pos	1.5	49.64	46.78	46.62	54.36
t_2	bal	hom	1.5	55.50	57.42	52.06	65.62
t_2	bal	neg	1.5	31.10	37.60	27.40	40.68
t_2	bal	pos	1.5	27.82	32.30	26.52	42.26
t_2	unb	hom	1.5	36.50	44.06	32.46	50.12
t_2	unb	neg	1.5	16.48	24.32	13.20	24.48
t_2	unb	pos	1.5	22.42	28.68	20.42	39.40
t_3	bal	hom	1.5	65.00	63.94	61.72	72.00
t_3	bal	neg	1.5	38.16	41.14	33.86	45.42
t_3	bal	pos	1.5	36.02	37.44	34.32	46.90
t_3	unb	hom	1.5	49.38	53.58	44.60	58.16
t_3	unb	neg	1.5	23.48	30.90	19.02	30.20
t_3	unb	pos	1.5	30.08	34.14	27.72	44.22

Table 58: Empirical Power for $\delta = 1.5$ in % of non-inferiority Dunnett-type tests with interval-based estimator

Setting				Method			
Distribution	Design		δ	asymp. MCTP	boot. MCTP	B. asymp.	B. perm.
χ_3^2	bal	hom	1.5	25.04	9.68	23.56	24.98
χ_3^2	bal	neg	1.5	18.08	7.58	16.52	17.72
χ_3^2	bal	pos	1.5	11.94	3.08	11.54	11.92
χ_3^2	unb	hom	1.5	29.56	12.24	27.90	26.82
χ_3^2	unb	neg	1.5	21.10	9.06	19.20	19.12
χ_3^2	unb	pos	1.5	13.36	3.18	12.88	12.10
$\mathcal{LN}(0,1)$	bal	hom	1.5	77.08	57.42	75.06	71.74
$\mathcal{LN}(0,1)$	bal	neg	1.5	51.04	35.40	48.60	46.60
$\mathcal{LN}(0,1)$	bal	pos	1.5	44.98	19.02	43.72	52.30
$\mathcal{LN}(0,1)$	unb	hom	1.5	74.78	56.60	72.24	66.26
$\mathcal{LN}(0,1)$	unb	neg	1.5	50.12	33.80	47.12	42.00
$\mathcal{LN}(0,1)$	unb	pos	1.5	50.92	25.12	48.80	53.54
$\mathcal{N}(0,1)$	bal	hom	1.5	78.52	53.92	76.82	77.50
$\mathcal{N}(0,1)$	bal	neg	1.5	48.42	28.02	45.20	44.14
$\mathcal{N}(0,1)$	bal	pos	1.5	46.52	22.22	45.42	48.98
$\mathcal{N}(0,1)$	unb	hom	1.5	77.76	45.98	74.88	71.94
$\mathcal{N}(0,1)$	unb	neg	1.5	48.42	20.92	43.90	38.40
$\mathcal{N}(0,1)$	unb	pos	1.5	49.30	21.86	47.76	47.00
t_2	bal	hom	1.5	54.26	39.78	52.26	59.58
t_2	bal	neg	1.5	29.40	20.38	27.14	32.34
t_2	bal	pos	1.5	27.38	16.18	26.54	36.46
t_2	unb	hom	1.5	55.10	32.62	51.68	55.04
t_2	unb	neg	1.5	29.12	14.74	26.02	27.26
t_2	unb	pos	1.5	30.24	14.78	28.82	35.28
t_3	bal	hom	1.5	63.16	44.86	61.02	65.84
t_3	bal	neg	1.5	35.50	22.34	32.82	35.50
t_3	bal	pos	1.5	33.84	18.08	32.82	41.10
t_3	unb	hom	1.5	61.96	37.54	59.22	59.24
t_3	unb	neg	1.5	36.40	17.98	32.72	31.66
t_3	unb	pos	1.5	36.26	17.24	34.62	38.36

Table 59: Empirical Power for $\delta = 1.5$ in % of two-sided Dunnett-type tests with kernel estimator

Setting				Method			
Distribution	Design		δ	asymp. MCTP	boot. MCTP	B. asymp.	B. perm.
χ^2_3	bal	hom	1.5	31.86	22.70	29.82	32.02
χ^2_3	bal	neg	1.5	22.22	17.24	20.08	22.44
χ^2_3	bal	pos	1.5	15.38	10.04	14.76	15.68
χ^2_3	unb	hom	1.5	36.82	27.26	33.94	31.74
χ^2_3	unb	neg	1.5	26.50	20.70	23.46	22.32
χ^2_3	unb	pos	1.5	17.20	12.80	16.06	14.78
$\mathcal{LN}(0,1)$	bal	hom	1.5	83.34	70.86	81.00	79.28
$\mathcal{LN}(0,1)$	bal	neg	1.5	59.42	47.90	55.74	55.06
$\mathcal{LN}(0,1)$	bal	pos	1.5	56.06	41.98	54.08	62.14
$\mathcal{LN}(0,1)$	unb	hom	1.5	80.52	71.12	77.54	70.14
$\mathcal{LN}(0,1)$	unb	neg	1.5	57.46	48.84	52.78	45.46
$\mathcal{LN}(0,1)$	unb	pos	1.5	60.52	48.28	57.80	58.30
$\mathcal{N}(0,1)$	bal	hom	1.5	85.32	70.38	83.62	83.98
$\mathcal{N}(0,1)$	bal	neg	1.5	58.26	46.38	53.76	54.42
$\mathcal{N}(0,1)$	bal	pos	1.5	56.00	40.74	54.58	58.64
$\mathcal{N}(0,1)$	unb	hom	1.5	84.96	66.72	81.90	78.36
$\mathcal{N}(0,1)$	unb	neg	1.5	58.08	42.30	51.56	45.98
$\mathcal{N}(0,1)$	unb	pos	1.5	58.50	39.60	55.92	53.86
t_2	bal	hom	1.5	65.08	58.18	61.94	70.16
t_2	bal	neg	1.5	39.18	37.80	34.40	41.62
t_2	bal	pos	1.5	35.92	31.90	34.22	45.44
t_2	unb	hom	1.5	65.80	54.64	61.22	63.16
t_2	unb	neg	1.5	38.44	32.86	33.04	34.22
t_2	unb	pos	1.5	39.96	31.68	37.44	42.28
t_3	bal	hom	1.5	72.74	62.30	69.98	74.48
t_3	bal	neg	1.5	45.02	40.44	40.58	44.88
t_3	bal	pos	1.5	43.34	34.52	41.62	50.56
t_3	unb	hom	1.5	71.82	58.56	67.48	67.60
t_3	unb	neg	1.5	45.46	37.86	40.22	38.24
t_3	unb	pos	1.5	45.96	34.20	43.42	46.62

Table 60: Empirical Power for $\delta = 1.5$ in % of non-inferiority Dunnett-type tests with kernel estimator

Setting				Method			
Distribution	Design		δ	asymp. MCTP	boot. MCTP	B. asymp.	B. perm.
χ_3^2	bal	hom	1.5	29.62	28.30	26.62	30.84
χ_3^2	bal	neg	1.5	23.58	22.72	20.70	25.92
χ_3^2	bal	pos	1.5	11.36	11.24	9.64	12.76
χ_3^2	unb	hom	1.5	40.56	38.14	37.04	41.04
χ_3^2	unb	neg	1.5	32.96	30.16	29.36	34.74
χ_3^2	unb	pos	1.5	14.48	14.14	12.48	14.42
$\mathcal{LN}(0,1)$	bal	hom	1.5	82.52	83.28	79.94	82.64
$\mathcal{LN}(0,1)$	bal	neg	1.5	69.82	70.58	66.34	71.88
$\mathcal{LN}(0,1)$	bal	pos	1.5	39.94	41.72	36.14	48.88
$\mathcal{LN}(0,1)$	unb	hom	1.5	92.34	91.44	90.90	91.86
$\mathcal{LN}(0,1)$	unb	neg	1.5	84.20	82.08	81.68	85.40
$\mathcal{LN}(0,1)$	unb	pos	1.5	56.84	58.26	53.10	63.78
$\mathcal{N}(0,1)$	bal	hom	1.5	87.00	83.56	85.22	87.74
$\mathcal{N}(0,1)$	bal	neg	1.5	76.84	71.50	73.74	75.86
$\mathcal{N}(0,1)$	bal	pos	1.5	47.76	45.12	44.64	49.80
$\mathcal{N}(0,1)$	unb	hom	1.5	92.50	88.18	90.96	92.08
$\mathcal{N}(0,1)$	unb	neg	1.5	83.76	76.74	80.82	83.18
$\mathcal{N}(0,1)$	unb	pos	1.5	56.84	52.54	53.20	56.24
t_2	bal	hom	1.5	68.86	72.72	65.56	73.26
t_2	bal	neg	1.5	54.84	59.30	50.70	58.94
t_2	bal	pos	1.5	31.10	36.28	27.92	39.50
t_2	unb	hom	1.5	76.82	78.62	73.36	79.94
t_2	unb	neg	1.5	64.40	65.12	60.00	67.38
t_2	unb	pos	1.5	36.08	40.78	33.04	43.38
t_3	bal	hom	1.5	76.50	76.76	73.72	79.04
t_3	bal	neg	1.5	62.80	63.10	58.70	64.22
t_3	bal	pos	1.5	37.18	39.12	34.20	41.96
t_3	unb	hom	1.5	83.24	81.96	80.94	85.14
t_3	unb	neg	1.5	72.48	69.46	68.20	73.04
t_3	unb	pos	1.5	43.30	44.18	40.00	47.52

Table 61: Empirical Power for $\delta = 1.5$ in % of two-sided Tukey-type tests with bootstrap estimator

Setting				Method			
Distribution	Design		δ	asymp. MCTP	boot. MCTP	B. asymp.	B. perm.
χ^2_3	bal	hom	1.5	37.82	41.58	34.80	42.32
χ^2_3	bal	neg	1.5	31.88	33.82	28.60	35.88
χ^2_3	bal	pos	1.5	16.24	19.96	14.06	17.96
χ^2_3	unb	hom	1.5	49.76	51.34	46.68	49.32
χ^2_3	unb	neg	1.5	41.80	42.08	37.66	43.02
χ^2_3	unb	pos	1.5	20.22	24.50	17.64	19.44
$\mathcal{LN}(0,1)$	bal	hom	1.5	88.08	89.38	86.36	89.88
$\mathcal{LN}(0,1)$	bal	neg	1.5	77.70	78.10	74.98	81.30
$\mathcal{LN}(0,1)$	bal	pos	1.5	49.72	58.58	45.76	61.88
$\mathcal{LN}(0,1)$	unb	hom	1.5	95.34	95.22	94.34	94.76
$\mathcal{LN}(0,1)$	unb	neg	1.5	88.90	88.32	86.90	90.02
$\mathcal{LN}(0,1)$	unb	pos	1.5	66.66	72.52	63.30	71.90
$\mathcal{N}(0,1)$	bal	hom	1.5	92.28	90.12	90.72	92.78
$\mathcal{N}(0,1)$	bal	neg	1.5	83.36	81.80	81.36	84.52
$\mathcal{N}(0,1)$	bal	pos	1.5	57.66	58.88	54.20	60.54
$\mathcal{N}(0,1)$	unb	hom	1.5	95.48	93.04	94.58	95.18
$\mathcal{N}(0,1)$	unb	neg	1.5	89.66	86.64	87.84	89.54
$\mathcal{N}(0,1)$	unb	pos	1.5	65.68	65.20	62.60	64.92
t_2	bal	hom	1.5	76.96	81.30	74.46	82.32
t_2	bal	neg	1.5	64.92	71.00	61.56	70.82
t_2	bal	pos	1.5	40.06	48.60	36.54	49.60
t_2	unb	hom	1.5	84.54	86.52	81.84	86.96
t_2	unb	neg	1.5	73.32	77.78	69.98	77.00
t_2	unb	pos	1.5	45.70	53.14	42.22	53.32
t_3	bal	hom	1.5	83.94	84.80	81.52	86.78
t_3	bal	neg	1.5	71.86	74.72	68.82	75.66
t_3	bal	pos	1.5	46.06	51.30	42.62	52.86
t_3	unb	hom	1.5	89.06	89.24	87.16	90.40
t_3	unb	neg	1.5	80.36	81.60	77.46	81.50
t_3	unb	pos	1.5	52.62	57.08	49.68	56.94

Table 62: Empirical Power for $\delta = 1.5$ in % of non-inferiority Tukey-type tests with bootstrap estimator

Setting				Method			
Distribution	Design		δ	asymp. MCTP	boot. MCTP	B. asymp.	B. perm.
χ^2_3	bal	hom	1.5	40.28	17.28	36.72	37.24
χ^2_3	bal	neg	1.5	34.24	13.96	30.72	33.36
χ^2_3	bal	pos	1.5	16.32	4.34	14.34	12.90
χ^2_3	unb	hom	1.5	46.80	24.46	43.04	45.50
χ^2_3	unb	neg	1.5	38.50	18.84	34.38	39.30
χ^2_3	unb	pos	1.5	17.00	6.24	14.46	15.90
$\mathcal{LN}(0,1)$	bal	hom	1.5	94.26	77.50	92.98	92.54
$\mathcal{LN}(0,1)$	bal	neg	1.5	86.14	62.62	84.10	84.04
$\mathcal{LN}(0,1)$	bal	pos	1.5	61.52	28.70	57.46	59.40
$\mathcal{LN}(0,1)$	unb	hom	1.5	96.60	85.56	95.82	95.14
$\mathcal{LN}(0,1)$	unb	neg	1.5	89.96	72.42	87.98	88.42
$\mathcal{LN}(0,1)$	unb	pos	1.5	67.66	42.22	63.80	69.70
$\mathcal{N}(0,1)$	bal	hom	1.5	87.02	72.22	85.24	89.18
$\mathcal{N}(0,1)$	bal	neg	1.5	75.52	55.88	72.52	79.76
$\mathcal{N}(0,1)$	bal	pos	1.5	44.00	26.98	40.10	47.98
$\mathcal{N}(0,1)$	unb	hom	1.5	90.72	77.42	89.10	92.94
$\mathcal{N}(0,1)$	unb	neg	1.5	80.80	60.86	77.42	83.38
$\mathcal{N}(0,1)$	unb	pos	1.5	52.48	35.38	48.76	57.82
t_2	bal	hom	1.5	59.38	48.36	55.14	68.76
t_2	bal	neg	1.5	44.74	33.78	41.06	56.04
t_2	bal	pos	1.5	23.58	16.82	20.88	34.04
t_2	unb	hom	1.5	68.00	55.30	63.40	75.12
t_2	unb	neg	1.5	56.40	43.16	51.56	62.94
t_2	unb	pos	1.5	27.22	20.00	24.32	40.08
t_3	bal	hom	1.5	70.68	56.90	67.22	76.50
t_3	bal	neg	1.5	54.62	39.58	50.32	64.12
t_3	bal	pos	1.5	30.52	19.90	27.46	38.42
t_3	unb	hom	1.5	77.88	63.70	74.24	82.54
t_3	unb	neg	1.5	64.52	48.34	59.86	68.96
t_3	unb	pos	1.5	34.08	23.80	30.42	45.08

Table 63: Empirical Power for $\delta = 1.5$ in % of two-sided Tukey-type tests with interval-based estimator

Setting				Method			
Distribution	Design		δ	asymp. MCTP	boot. MCTP	B. asymp.	B. perm.
χ_3^2	bal	hom	1.5	49.22	31.54	46.00	48.76
χ_3^2	bal	neg	1.5	42.30	23.82	39.26	43.46
χ_3^2	bal	pos	1.5	21.42	12.84	18.60	18.30
χ_3^2	unb	hom	1.5	56.34	37.68	52.66	52.86
χ_3^2	unb	neg	1.5	48.28	28.64	44.30	47.00
χ_3^2	unb	pos	1.5	23.56	14.82	20.96	19.70
$\mathcal{LN}(0,1)$	bal	hom	1.5	96.70	85.38	95.88	96.18
$\mathcal{LN}(0,1)$	bal	neg	1.5	90.10	71.08	88.94	90.44
$\mathcal{LN}(0,1)$	bal	pos	1.5	70.10	48.88	66.76	70.68
$\mathcal{LN}(0,1)$	unb	hom	1.5	98.28	91.50	97.78	96.74
$\mathcal{LN}(0,1)$	unb	neg	1.5	93.04	80.44	91.78	91.70
$\mathcal{LN}(0,1)$	unb	pos	1.5	76.50	59.40	73.38	75.90
$\mathcal{N}(0,1)$	bal	hom	1.5	92.24	82.84	90.64	94.26
$\mathcal{N}(0,1)$	bal	neg	1.5	82.98	69.04	80.50	87.56
$\mathcal{N}(0,1)$	bal	pos	1.5	54.14	45.02	50.38	59.14
$\mathcal{N}(0,1)$	unb	hom	1.5	95.12	84.98	93.88	95.68
$\mathcal{N}(0,1)$	unb	neg	1.5	87.54	72.74	85.00	89.30
$\mathcal{N}(0,1)$	unb	pos	1.5	62.00	49.52	58.78	65.64
t_2	bal	hom	1.5	69.12	64.08	65.96	78.56
t_2	bal	neg	1.5	55.06	49.46	51.64	68.80
t_2	bal	pos	1.5	31.70	30.60	29.02	45.16
t_2	unb	hom	1.5	76.98	68.74	73.16	84.56
t_2	unb	neg	1.5	65.70	56.54	60.84	73.12
t_2	unb	pos	1.5	35.66	32.52	32.00	51.24
t_3	bal	hom	1.5	78.70	71.78	75.96	84.84
t_3	bal	neg	1.5	65.48	55.80	62.02	76.18
t_3	bal	pos	1.5	39.60	35.30	36.60	49.50
t_3	unb	hom	1.5	84.60	75.38	82.30	88.80
t_3	unb	neg	1.5	73.76	61.18	69.74	78.18
t_3	unb	pos	1.5	44.20	37.26	41.04	55.56

Table 64: Empirical Power for $\delta = 1.5$ in % of non-inferiority Tukey-type tests with interval-based estimator

Setting				Method			
Distribution	Design		δ	asymp. MCTP	boot. MCTP	B. asymp.	B. perm.
χ^2_3	bal	hom	1.5	38.54	13.44	35.52	35.50
χ^2_3	bal	neg	1.5	32.84	10.68	29.72	31.28
χ^2_3	bal	pos	1.5	15.26	3.86	13.30	12.70
χ^2_3	unb	hom	1.5	49.12	17.14	45.36	46.48
χ^2_3	unb	neg	1.5	41.28	13.10	37.60	41.24
χ^2_3	unb	pos	1.5	17.26	3.98	15.08	13.86
$\mathcal{LN}(0,1)$	bal	hom	1.5	95.02	74.38	93.78	92.64
$\mathcal{LN}(0,1)$	bal	neg	1.5	85.76	59.42	83.02	82.34
$\mathcal{LN}(0,1)$	bal	pos	1.5	57.64	23.74	52.24	61.80
$\mathcal{LN}(0,1)$	unb	hom	1.5	97.54	82.28	96.98	96.38
$\mathcal{LN}(0,1)$	unb	neg	1.5	91.10	66.60	89.68	90.44
$\mathcal{LN}(0,1)$	unb	pos	1.5	69.80	34.48	65.64	74.18
$\mathcal{N}(0,1)$	bal	hom	1.5	90.48	64.22	88.68	88.62
$\mathcal{N}(0,1)$	bal	neg	1.5	79.96	47.22	77.44	76.10
$\mathcal{N}(0,1)$	bal	pos	1.5	51.30	23.70	47.96	50.36
$\mathcal{N}(0,1)$	unb	hom	1.5	94.60	65.78	93.46	93.32
$\mathcal{N}(0,1)$	unb	neg	1.5	86.40	45.54	83.76	83.98
$\mathcal{N}(0,1)$	unb	pos	1.5	59.98	26.08	56.44	56.44
t_2	bal	hom	1.5	69.82	49.84	66.88	73.84
t_2	bal	neg	1.5	55.66	34.26	52.08	59.18
t_2	bal	pos	1.5	31.36	17.88	28.50	38.96
t_2	unb	hom	1.5	78.98	49.74	75.92	80.72
t_2	unb	neg	1.5	66.68	32.96	62.18	68.44
t_2	unb	pos	1.5	37.52	17.88	34.12	42.18
t_3	bal	hom	1.5	78.32	55.16	75.62	80.24
t_3	bal	neg	1.5	63.68	37.00	59.98	64.68
t_3	bal	pos	1.5	38.16	20.22	35.24	42.44
t_3	unb	hom	1.5	85.52	55.08	83.10	85.58
t_3	unb	neg	1.5	74.76	37.32	71.36	73.78
t_3	unb	pos	1.5	44.60	20.04	41.14	46.92

Table 65: Empirical Power for $\delta = 1.5$ in % of two-sided Tukey-type tests with kernel estimator

Setting				Method			
Distribution	Design		δ	asymp. MCTP	boot. MCTP	B. asymp.	B. perm.
χ^2_3	bal	hom	1.5	47.74	26.38	44.28	47.38
χ^2_3	bal	neg	1.5	41.76	19.84	38.58	41.98
χ^2_3	bal	pos	1.5	20.04	10.02	17.64	18.04
χ^2_3	unb	hom	1.5	58.82	34.58	55.34	56.04
χ^2_3	unb	neg	1.5	50.88	26.90	47.26	50.30
χ^2_3	unb	pos	1.5	23.88	12.20	21.16	19.60
$\mathcal{LN}(0,1)$	bal	hom	1.5	97.34	85.90	96.70	96.88
$\mathcal{LN}(0,1)$	bal	neg	1.5	90.66	70.46	89.10	89.94
$\mathcal{LN}(0,1)$	bal	pos	1.5	69.20	45.78	64.98	74.32
$\mathcal{LN}(0,1)$	unb	hom	1.5	98.82	92.28	98.44	98.02
$\mathcal{LN}(0,1)$	unb	neg	1.5	94.58	81.70	93.34	94.30
$\mathcal{LN}(0,1)$	unb	pos	1.5	78.90	59.52	76.26	82.40
$\mathcal{N}(0,1)$	bal	hom	1.5	94.20	77.30	93.16	93.78
$\mathcal{N}(0,1)$	bal	neg	1.5	86.68	63.54	84.66	85.18
$\mathcal{N}(0,1)$	bal	pos	1.5	60.08	38.32	57.18	60.84
$\mathcal{N}(0,1)$	unb	hom	1.5	97.32	81.64	96.68	96.56
$\mathcal{N}(0,1)$	unb	neg	1.5	92.18	68.52	90.24	90.32
$\mathcal{N}(0,1)$	unb	pos	1.5	68.52	41.92	65.66	65.74
t_2	bal	hom	1.5	78.16	64.62	75.88	82.62
t_2	bal	neg	1.5	65.52	51.36	62.70	70.88
t_2	bal	pos	1.5	40.22	29.96	37.12	49.54
t_2	unb	hom	1.5	86.22	68.12	83.82	87.56
t_2	unb	neg	1.5	75.82	55.66	72.48	78.10
t_2	unb	pos	1.5	46.76	30.90	43.96	52.08
t_3	bal	hom	1.5	85.14	69.48	83.26	87.42
t_3	bal	neg	1.5	74.06	54.26	71.08	75.50
t_3	bal	pos	1.5	47.58	32.72	44.34	52.84
t_3	unb	hom	1.5	90.66	73.52	89.22	91.28
t_3	unb	neg	1.5	82.64	60.38	79.64	82.78
t_3	unb	pos	1.5	54.50	34.66	51.28	56.50

Table 66: Empirical Power for $\delta = 1.5$ in % of non-inferiority Tukey-type tests with kernel estimator

Setting				Method			
Distribution	Design		δ	asymp. MCTP	boot. MCTP	B. asymp.	B. perm.
χ_3^2	bal	hom	1.5	27.06	28.00	25.62	31.52
χ_3^2	bal	neg	1.5	18.50	20.16	17.42	23.12
χ_3^2	bal	pos	1.5	11.12	12.82	10.16	13.50
χ_3^2	unb	hom	1.5	35.50	36.36	33.76	42.62
χ_3^2	unb	neg	1.5	24.04	26.24	22.52	31.48
χ_3^2	unb	pos	1.5	13.44	15.02	12.74	16.66
$\mathcal{LN}(0,1)$	bal	hom	1.5	80.36	84.08	79.16	82.94
$\mathcal{LN}(0,1)$	bal	neg	1.5	62.74	67.72	60.32	67.30
$\mathcal{LN}(0,1)$	bal	pos	1.5	37.34	44.36	35.36	49.26
$\mathcal{LN}(0,1)$	unb	hom	1.5	89.98	90.62	88.74	93.46
$\mathcal{LN}(0,1)$	unb	neg	1.5	71.82	75.22	69.56	79.32
$\mathcal{LN}(0,1)$	unb	pos	1.5	51.56	57.74	49.50	67.68
$\mathcal{N}(0,1)$	bal	hom	1.5	88.02	85.84	87.10	90.12
$\mathcal{N}(0,1)$	bal	neg	1.5	77.76	75.56	76.42	79.84
$\mathcal{N}(0,1)$	bal	pos	1.5	47.46	47.56	45.66	52.50
$\mathcal{N}(0,1)$	unb	hom	1.5	92.82	90.72	92.16	94.66
$\mathcal{N}(0,1)$	unb	neg	1.5	82.80	79.14	81.40	85.50
$\mathcal{N}(0,1)$	unb	pos	1.5	56.94	55.10	55.66	62.34
t_2	bal	hom	1.5	70.16	75.98	68.66	77.38
t_2	bal	neg	1.5	55.16	62.88	53.76	63.38
t_2	bal	pos	1.5	30.68	39.48	29.32	42.16
t_2	unb	hom	1.5	75.96	81.20	74.20	84.48
t_2	unb	neg	1.5	56.94	64.88	54.56	67.12
t_2	unb	pos	1.5	36.06	44.62	34.48	50.00
t_3	bal	hom	1.5	77.26	79.12	76.24	82.22
t_3	bal	neg	1.5	62.60	66.44	60.72	68.40
t_3	bal	pos	1.5	37.28	41.98	35.74	45.68
t_3	unb	hom	1.5	83.52	84.30	82.52	88.88
t_3	unb	neg	1.5	68.12	70.26	65.56	73.76
t_3	unb	pos	1.5	43.28	47.78	42.02	54.08

Table 67: Empirical Power for $\delta = 1.5$ in % of two-sided Grand-mean-type tests with bootstrap estimator

Setting				Method			
Distribution	Design		δ	asymp. MCTP	boot. MCTP	B. asymp.	B. perm.
χ^2_3	bal	hom	1.5	27.84	33.14	27.72	38.14
χ^2_3	bal	neg	1.5	19.36	24.78	19.30	28.70
χ^2_3	bal	pos	1.5	9.76	13.26	9.72	15.76
χ^2_3	unb	hom	1.5	38.28	43.02	38.12	46.88
χ^2_3	unb	neg	1.5	26.30	32.02	26.08	35.32
χ^2_3	unb	pos	1.5	12.42	16.56	12.34	18.20
$\mathcal{LN}(0,1)$	bal	hom	1.5	83.16	88.82	83.00	91.78
$\mathcal{LN}(0,1)$	bal	neg	1.5	66.74	75.18	66.42	82.04
$\mathcal{LN}(0,1)$	bal	pos	1.5	38.42	52.00	38.16	59.78
$\mathcal{LN}(0,1)$	unb	hom	1.5	92.28	94.24	92.20	95.34
$\mathcal{LN}(0,1)$	unb	neg	1.5	76.04	82.04	75.46	83.96
$\mathcal{LN}(0,1)$	unb	pos	1.5	55.58	66.72	55.36	72.96
$\mathcal{N}(0,1)$	bal	hom	1.5	91.52	90.64	91.44	93.52
$\mathcal{N}(0,1)$	bal	neg	1.5	82.14	81.68	82.08	85.20
$\mathcal{N}(0,1)$	bal	pos	1.5	51.48	53.00	51.40	58.56
$\mathcal{N}(0,1)$	unb	hom	1.5	94.98	93.72	94.96	95.90
$\mathcal{N}(0,1)$	unb	neg	1.5	86.50	84.76	86.32	87.82
$\mathcal{N}(0,1)$	unb	pos	1.5	61.64	60.80	61.54	65.14
t_2	bal	hom	1.5	74.88	81.08	74.78	82.74
t_2	bal	neg	1.5	61.90	70.26	61.72	71.20
t_2	bal	pos	1.5	34.30	43.80	34.12	47.30
t_2	unb	hom	1.5	81.62	86.24	81.40	87.24
t_2	unb	neg	1.5	64.18	71.90	63.64	70.72
t_2	unb	pos	1.5	42.28	50.24	42.14	53.20
t_3	bal	hom	1.5	81.66	84.10	81.54	87.16
t_3	bal	neg	1.5	69.32	73.32	69.22	76.48
t_3	bal	pos	1.5	41.10	46.64	40.92	50.90
t_3	unb	hom	1.5	87.88	88.86	87.78	91.04
t_3	unb	neg	1.5	74.16	77.10	73.72	78.08
t_3	unb	pos	1.5	49.36	54.06	49.24	57.34

Table 68: Empirical Power for $\delta = 1.5$ in % of non-inferiority Grand-mean-type tests with bootstrap estimator

Setting				Method			
Distribution	Design		δ	asymp. MCTP	boot. MCTP	B. asymp.	B. perm.
χ_3^2	bal	hom	1.5	37.74	16.68	35.94	34.52
χ_3^2	bal	neg	1.5	28.12	11.92	26.64	27.78
χ_3^2	bal	pos	1.5	17.28	5.98	16.34	13.16
χ_3^2	unb	hom	1.5	40.80	21.54	39.20	47.24
χ_3^2	unb	neg	1.5	29.32	14.90	27.32	37.38
χ_3^2	unb	pos	1.5	16.60	7.04	15.66	18.18
$\mathcal{LN}(0,1)$	bal	hom	1.5	93.32	77.22	92.96	89.36
$\mathcal{LN}(0,1)$	bal	neg	1.5	81.16	57.40	79.50	76.08
$\mathcal{LN}(0,1)$	bal	pos	1.5	58.70	30.42	56.56	54.50
$\mathcal{LN}(0,1)$	unb	hom	1.5	95.18	83.70	94.40	96.16
$\mathcal{LN}(0,1)$	unb	neg	1.5	80.38	62.84	78.66	84.40
$\mathcal{LN}(0,1)$	unb	pos	1.5	62.86	42.20	60.86	73.18
$\mathcal{N}(0,1)$	bal	hom	1.5	88.08	77.08	87.26	90.86
$\mathcal{N}(0,1)$	bal	neg	1.5	76.54	62.38	75.20	82.86
$\mathcal{N}(0,1)$	bal	pos	1.5	43.88	31.38	42.22	50.26
$\mathcal{N}(0,1)$	unb	hom	1.5	92.04	83.50	91.28	96.12
$\mathcal{N}(0,1)$	unb	neg	1.5	76.40	63.20	74.38	86.06
$\mathcal{N}(0,1)$	unb	pos	1.5	52.88	39.58	51.28	64.68
t_2	bal	hom	1.5	60.56	57.64	59.22	73.44
t_2	bal	neg	1.5	45.06	41.50	43.28	60.98
t_2	bal	pos	1.5	23.06	20.88	21.66	37.66
t_2	unb	hom	1.5	52.14	51.22	49.60	74.06
t_2	unb	neg	1.5	28.70	26.84	26.60	51.30
t_2	unb	pos	1.5	22.58	21.22	20.98	47.08
t_3	bal	hom	1.5	71.26	64.52	70.16	79.54
t_3	bal	neg	1.5	55.46	47.32	53.66	68.66
t_3	bal	pos	1.5	30.30	25.18	29.32	41.94
t_3	unb	hom	1.5	70.66	66.02	68.68	84.62
t_3	unb	neg	1.5	44.18	38.24	41.50	63.58
t_3	unb	pos	1.5	32.14	26.46	30.66	53.26

Table 69: Empirical Power for $\delta = 1.5$ in % of two-sided Grand-mean-type tests with interval-based estimator

Setting				Method			
Distribution	Design		δ	asymp. MCTP	boot. MCTP	B. asymp.	B. perm.
χ_3^2	bal	hom	1.5	35.28	24.52	35.18	44.78
χ_3^2	bal	neg	1.5	26.18	16.32	26.14	36.48
χ_3^2	bal	pos	1.5	12.38	6.36	12.30	15.90
χ_3^2	unb	hom	1.5	42.70	31.50	42.50	50.70
χ_3^2	unb	neg	1.5	31.08	21.86	30.78	40.00
χ_3^2	unb	pos	1.5	14.38	8.84	14.34	18.62
$\mathcal{LN}(0,1)$	bal	hom	1.5	94.00	86.72	93.86	97.04
$\mathcal{LN}(0,1)$	bal	neg	1.5	81.96	69.70	81.80	90.40
$\mathcal{LN}(0,1)$	bal	pos	1.5	57.14	41.46	56.92	69.54
$\mathcal{LN}(0,1)$	unb	hom	1.5	96.02	90.64	95.90	97.52
$\mathcal{LN}(0,1)$	unb	neg	1.5	83.26	73.28	82.84	88.54
$\mathcal{LN}(0,1)$	unb	pos	1.5	65.62	53.02	65.34	78.52
$\mathcal{N}(0,1)$	bal	hom	1.5	91.80	87.38	91.78	95.36
$\mathcal{N}(0,1)$	bal	neg	1.5	81.78	75.78	81.68	89.16
$\mathcal{N}(0,1)$	bal	pos	1.5	48.24	41.36	48.10	59.24
$\mathcal{N}(0,1)$	unb	hom	1.5	95.20	91.66	95.16	97.32
$\mathcal{N}(0,1)$	unb	neg	1.5	82.60	76.26	82.26	89.22
$\mathcal{N}(0,1)$	unb	pos	1.5	58.74	51.72	58.68	67.98
t_2	bal	hom	1.5	67.84	68.92	67.76	79.42
t_2	bal	neg	1.5	52.42	54.20	52.28	69.26
t_2	bal	pos	1.5	27.36	30.04	27.24	43.10
t_2	unb	hom	1.5	60.44	65.30	59.76	79.72
t_2	unb	neg	1.5	35.56	39.62	34.86	58.34
t_2	unb	pos	1.5	28.72	31.42	28.52	51.82
t_3	bal	hom	1.5	76.66	75.02	76.58	85.82
t_3	bal	neg	1.5	63.20	61.40	63.00	77.48
t_3	bal	pos	1.5	35.10	33.50	35.00	47.80
t_3	unb	hom	1.5	77.38	77.92	76.82	88.18
t_3	unb	neg	1.5	52.76	52.94	51.94	69.90
t_3	unb	pos	1.5	38.38	38.44	38.04	57.32

Table 70: Empirical Power for $\delta = 1.5$ in % of non-inferiority Grand-mean-type tests with interval-based estimator

Setting				Method			
Distribution	Design		δ	asymp. MCTP	boot. MCTP	B. asymp.	B. perm.
χ^2_3	bal	hom	1.5	36.06	12.44	34.48	32.84
χ^2_3	bal	neg	1.5	27.36	8.96	25.72	26.20
χ^2_3	bal	pos	1.5	16.66	4.66	15.54	13.20
χ^2_3	unb	hom	1.5	42.54	15.38	40.78	46.88
χ^2_3	unb	neg	1.5	32.04	10.86	30.06	37.14
χ^2_3	unb	pos	1.5	16.96	4.74	15.84	15.92
$\mathcal{LN}(0,1)$	bal	hom	1.5	94.04	73.76	93.48	90.32
$\mathcal{LN}(0,1)$	bal	neg	1.5	80.46	54.20	79.06	75.82
$\mathcal{LN}(0,1)$	bal	pos	1.5	54.50	24.68	51.94	55.14
$\mathcal{LN}(0,1)$	unb	hom	1.5	96.04	80.06	95.50	96.58
$\mathcal{LN}(0,1)$	unb	neg	1.5	82.62	58.64	80.82	84.66
$\mathcal{LN}(0,1)$	unb	pos	1.5	63.98	35.62	61.64	75.72
$\mathcal{N}(0,1)$	bal	hom	1.5	91.14	69.94	90.24	91.12
$\mathcal{N}(0,1)$	bal	neg	1.5	82.34	55.28	81.22	81.20
$\mathcal{N}(0,1)$	bal	pos	1.5	51.50	26.92	49.88	52.26
$\mathcal{N}(0,1)$	unb	hom	1.5	95.44	74.54	95.12	96.44
$\mathcal{N}(0,1)$	unb	neg	1.5	86.56	52.90	85.30	87.30
$\mathcal{N}(0,1)$	unb	pos	1.5	60.12	30.46	58.82	63.12
t_2	bal	hom	1.5	71.90	57.12	70.92	77.76
t_2	bal	neg	1.5	57.00	41.00	55.62	64.24
t_2	bal	pos	1.5	31.02	20.66	29.56	42.08
t_2	unb	hom	1.5	80.22	57.26	79.12	86.30
t_2	unb	neg	1.5	63.24	36.76	61.02	70.60
t_2	unb	pos	1.5	38.14	21.42	36.64	49.30
t_3	bal	hom	1.5	78.78	60.60	77.86	82.40
t_3	bal	neg	1.5	65.40	44.60	63.46	68.74
t_3	bal	pos	1.5	38.42	22.92	37.04	45.50
t_3	unb	hom	1.5	86.16	63.18	85.28	90.30
t_3	unb	neg	1.5	72.74	42.08	70.66	76.22
t_3	unb	pos	1.5	45.30	23.94	43.84	53.90

Table 71: Empirical Power for $\delta = 1.5$ in % of two-sided Grand-mean-type tests with kernel estimator

Setting				Method			
Distribution	Design		δ	asymp. MCTP	boot. MCTP	B. asymp.	B. perm.
χ^2_3	bal	hom	1.5	33.46	18.96	33.40	42.08
χ^2_3	bal	neg	1.5	25.30	13.62	25.26	34.70
χ^2_3	bal	pos	1.5	11.68	5.30	11.70	15.38
χ^2_3	unb	hom	1.5	44.78	22.34	44.60	53.14
χ^2_3	unb	neg	1.5	33.82	15.78	33.72	41.92
χ^2_3	unb	pos	1.5	14.06	5.82	13.96	17.96
$\mathcal{LN}(0,1)$	bal	hom	1.5	94.92	85.68	94.86	97.54
$\mathcal{LN}(0,1)$	bal	neg	1.5	81.72	68.62	81.64	90.10
$\mathcal{LN}(0,1)$	bal	pos	1.5	53.52	37.52	53.26	71.84
$\mathcal{LN}(0,1)$	unb	hom	1.5	97.04	87.60	96.84	98.18
$\mathcal{LN}(0,1)$	unb	neg	1.5	84.96	68.12	84.44	90.20
$\mathcal{LN}(0,1)$	unb	pos	1.5	67.52	47.96	67.28	82.26
$\mathcal{N}(0,1)$	bal	hom	1.5	93.78	79.30	93.76	95.00
$\mathcal{N}(0,1)$	bal	neg	1.5	85.68	66.32	85.68	86.38
$\mathcal{N}(0,1)$	bal	pos	1.5	54.24	34.96	54.14	58.86
$\mathcal{N}(0,1)$	unb	hom	1.5	97.36	81.58	97.34	97.60
$\mathcal{N}(0,1)$	unb	neg	1.5	90.28	62.48	90.12	89.56
$\mathcal{N}(0,1)$	unb	pos	1.5	64.24	37.16	64.22	66.78
t_2	bal	hom	1.5	76.70	66.78	76.66	83.06
t_2	bal	neg	1.5	64.40	52.34	64.24	71.58
t_2	bal	pos	1.5	34.54	27.22	34.46	46.78
t_2	unb	hom	1.5	85.34	66.88	85.26	89.12
t_2	unb	neg	1.5	70.84	46.62	70.32	74.10
t_2	unb	pos	1.5	42.90	27.60	42.86	52.88
t_3	bal	hom	1.5	83.56	70.88	83.54	88.06
t_3	bal	neg	1.5	71.64	56.24	71.54	76.66
t_3	bal	pos	1.5	41.98	30.24	41.88	50.78
t_3	unb	hom	1.5	89.92	71.20	89.88	92.28
t_3	unb	neg	1.5	78.28	51.78	78.06	80.12
t_3	unb	pos	1.5	50.94	30.32	50.82	57.68

Table 72: Empirical Power for $\delta = 1.5$ in % of non-inferiority Grand-mean-type tests with kernel estimator

3 Plots for Empirical Local Power

In this section, boxplots to analyze the local power of the different methods are provided in Figures 11–18. In detail, the rejection rates for the false local hypotheses are plotted separately. Here, we only show the results with median difference $\delta = 1.5$ for the sake of clarity. Note that for the Grand-mean-type contrast matrix, the two-sided hypotheses $\mathcal{H}_{0,1} : m_1 - \bar{m} = 0, \mathcal{H}_{0,2} : m_2 - \bar{m} = 0$ and $\mathcal{H}_{0,3} : m_2 - \bar{m} = 0$ are false under the considered simulation scenario with $m_1 = m_2 = m_3 = 0$ and $m_4 = 1.5$ but the non-

inferiority hypotheses $\mathcal{H}_{0,1}^I : m_1 - \bar{m} \leq 0, \mathcal{H}_{0,2}^I : m_2 - \bar{m} = 0$ and $\mathcal{H}_{0,3}^I : m_3 - \bar{m} = 0$ are true, which is why only the rejection rates of the two-sided hypotheses are shown in the corresponding figures. Moreover, the empirical local power is relatively small for these hypotheses, which can be explained by the smaller effect parameter of $m_\ell - \bar{m} = -\delta/4$ for $\ell \in \{1, 2, 3\}$.

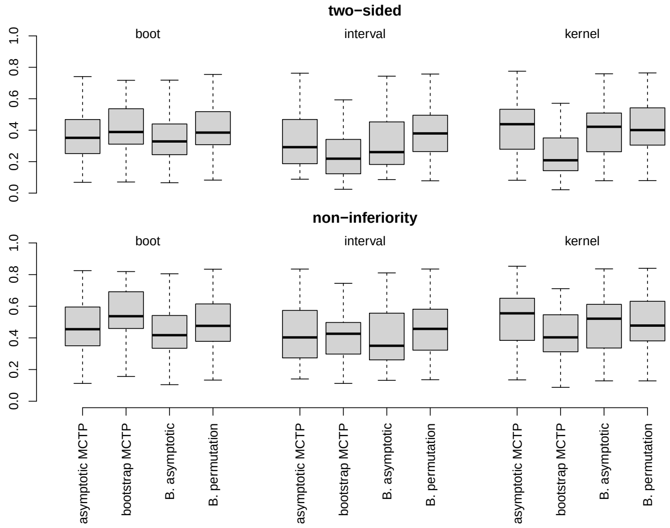

Figure 11: Empirical local power of $\mathcal{H}_{0,3} : m_4 - m_1 = 0$ (top) and $\mathcal{H}_{0,3}^I : m_4 - m_1 \leq 0$ (bottom) with $\delta = 1.5$ for Dunnett-type contrasts with different variance estimators.

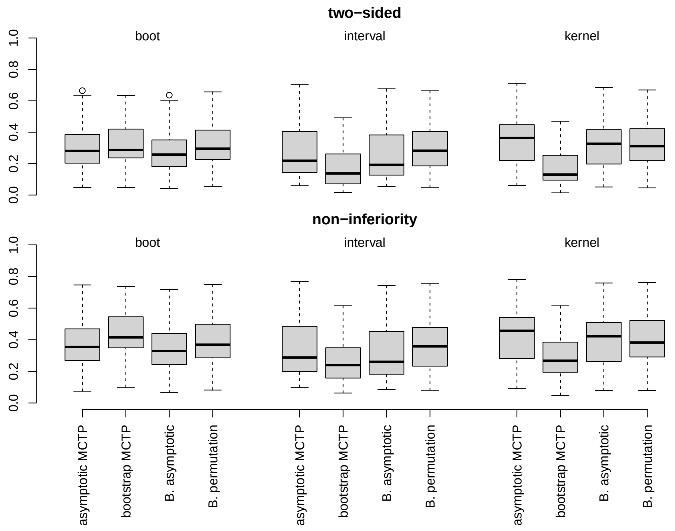

Figure 12: Empirical local power of $\mathcal{H}_{0,3} : m_4 - m_1 = 0$ (top) and $\mathcal{H}^I_{0,3} : m_4 - m_1 \leq 0$ (bottom) with $\delta = 1.5$ for Tukey-type contrasts with different variance estimators.

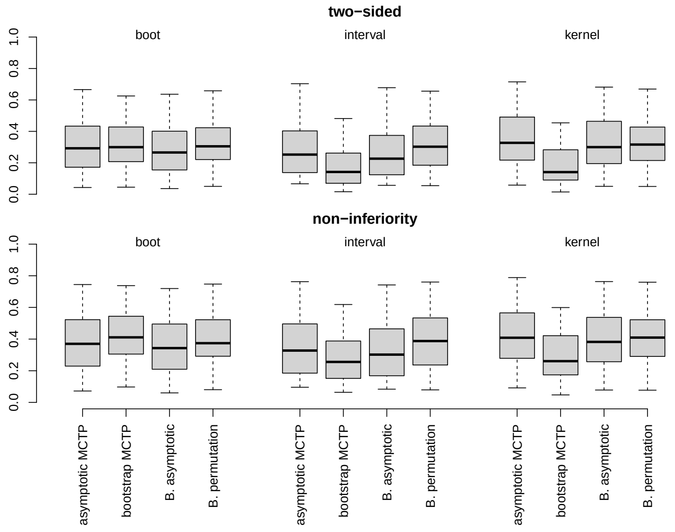

Figure 13: Empirical local power of $\mathcal{H}_{0,5} : m_4 - m_2 = 0$ (top) and $\mathcal{H}^I_{0,5} : m_4 - m_2 \leq 0$ (bottom) with $\delta = 1.5$ for Tukey-type contrasts with different variance estimators.

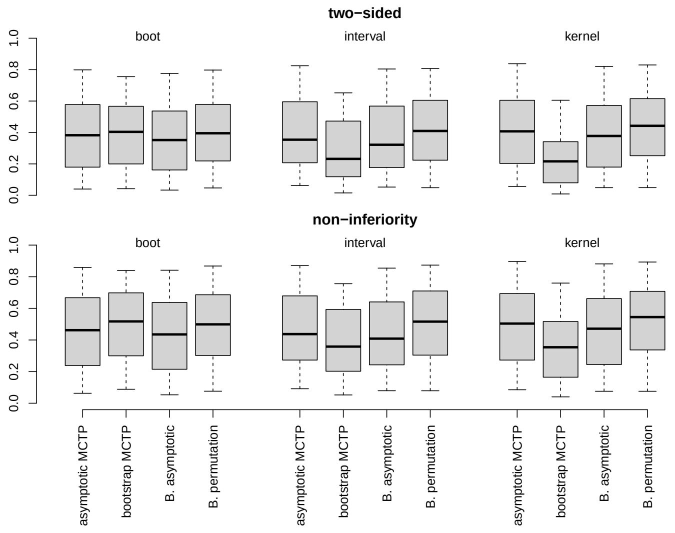

Figure 14: Empirical local power of $\mathcal{H}_{0,6} : m_4 - m_3 = 0$ (top) and $\mathcal{H}^I_{0,6} : m_4 - m_3 \leq 0$ (bottom) with $\delta = 1.5$ for Tukey-type contrasts with different variance estimators.

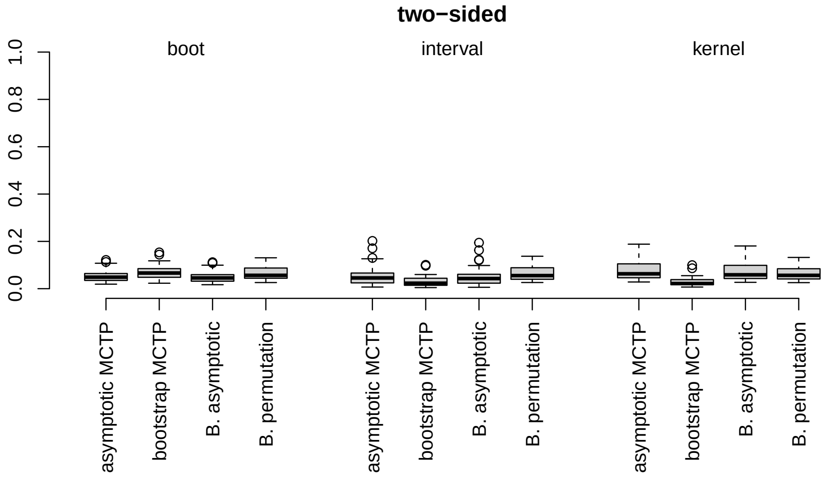

Figure 15: Empirical local power of $\mathcal{H}_{0,1} : m_1 - \bar{m} = 0$ with $\delta = 1.5$ for Grand-mean-type contrasts with different variance estimators.

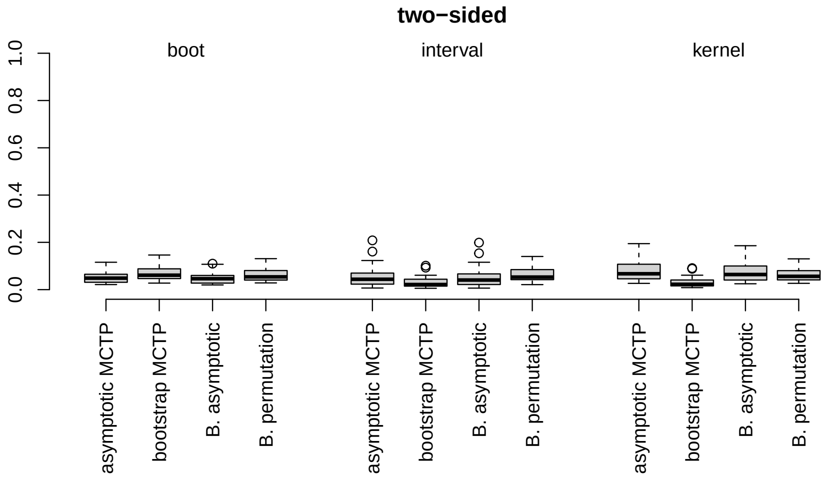

Figure 16: Empirical local power of $\mathcal{H}_{0,2} : m_2 - \bar{m} = 0$ with $\delta = 1.5$ for Grand-mean-type contrasts with different variance estimators.

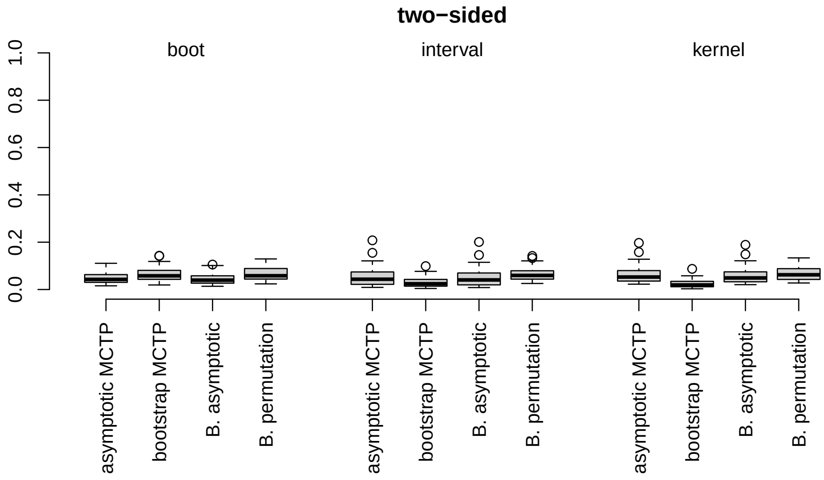

Figure 17: Empirical local power of $\mathcal{H}_{0,3} : m_3 - \bar{m} = 0$ with $\delta = 1.5$ for Grand-mean-type contrasts with different variance estimators.

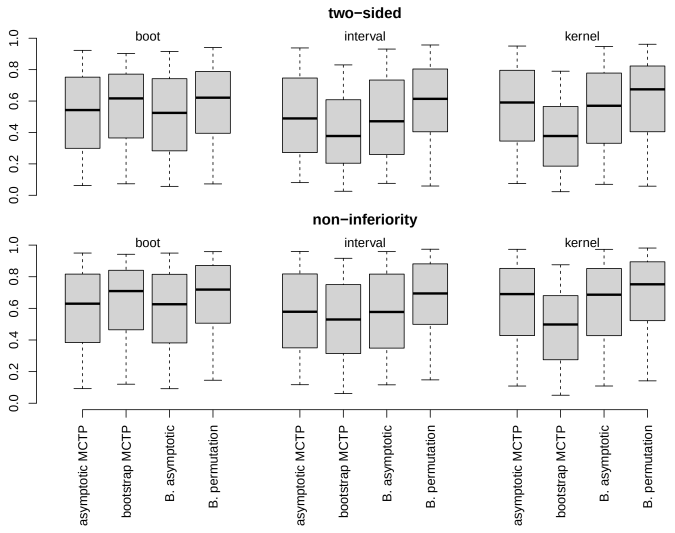

Figure 18: Empirical local power of $\mathcal{H}_{0,4} : m_4 - \bar{m} = 0$ (top) and of $\mathcal{H}^I_{0,4} : m_4 - \bar{m} \leq 0$ (bottom) with $\delta = 1.5$ for Grand-mean-type contrasts with different variance estimators.

4 Additional Simulation Study for medians and interquartile ranges

We conducted additional simulations to consider the type I error and power for simultaneous comparisons of medians and interquartile ranges (IQRs) across $k = 4$ different groups. Hence, we choose the probabilities $p_1 = 0.25, p_2 = 0.5, p_3 = 0.75$ with $m = 3$. As contrast matrices, the proposed matrices in the end of Section 2 of the paper are used, i.e. Kronecker products of Dunnett-, Tukey-, and Grand-mean-type matrices with

$$\begin{bmatrix} 0 & 1 & 0 \\ -1 & 0 & 1 \end{bmatrix}.$$

All other parameters as, e.g., the data generation is as described in Section 4.1 of the paper. Now, also the IQRs are compared and, thus, the settings with positive and negative pairing $(\boldsymbol{\sigma}_2, \boldsymbol{\sigma}_3)$ become settings under the alternative. Hence, the model implicates that the data is exchangeable under the null hypothesis and, thus, the global permutation test should be exact.

In Figures 19–21, it is observable that the Bonferroni-adjusted permutation test is accurate or slightly conservative (due to the Bonferroni-correction) regarding the type I error

control. The asymptotic approaches tend to be conservative except for the two-sided Gand-mean-type hypotheses in combination with the kernel estimator, where it occurs a liberal behaviour of the asymptotic tests. The groupwise bootstrap tests perform too conservative in nearly all scenarios. Only for Dunnett-type contrasts in combination with the bootstrap variance estimator, the groupwise bootstrap tests seem to perform accurate regarding the empirical FWER. The permutation approach can outperform the other methods regarding the power in nearly all scenarios, see Figures 22–33. All in all, the results of this additional simulation study are similar to the results in Section 4 of the paper.

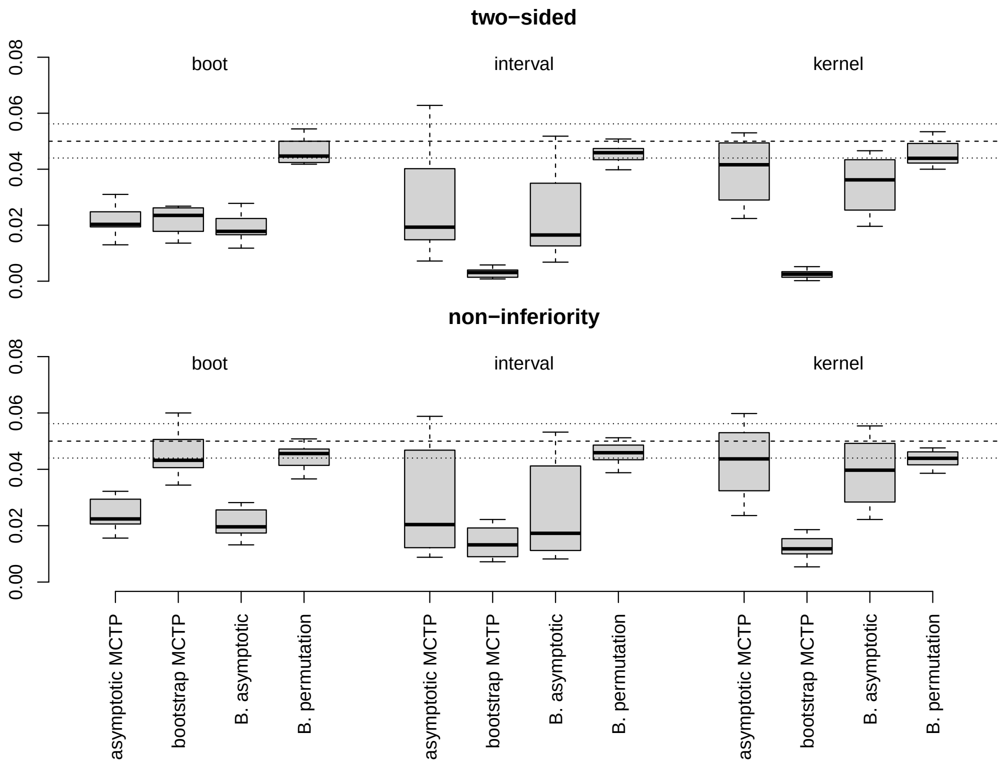

Figure 19: Empirical FWERs for Dunnett-type contrasts with different hypotheses (top: two-sided and bottom: non-inferiority) and variance estimators (from left to right: bootstrap, interval-based or kernel). The dashed line represents the desired level of significance of $\alpha = 5\%$ and the dotted lines represent the Binomial interval $[0.044, 0.0562]$ for $N_{sim} = 5000$ repetitions.

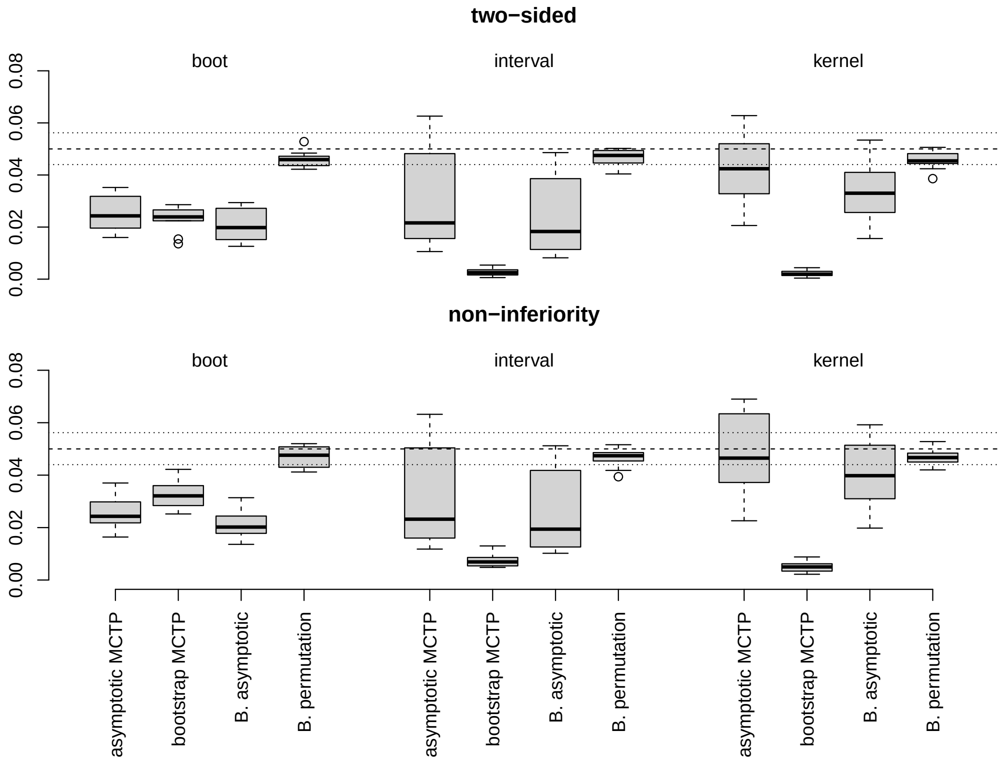

Figure 20: Empirical FWERs for Tukey-type contrasts with different hypotheses (top: two-sided and bottom: non-inferiority) and variance estimators (from left to right: bootstrap, interval-based or kernel). The dashed line represents the desired level of significance of $\alpha = 5\%$ and the dotted lines represent the Binomial interval $[0.044, 0.0562]$ for $N_{sim} = 5000$ repetitions.

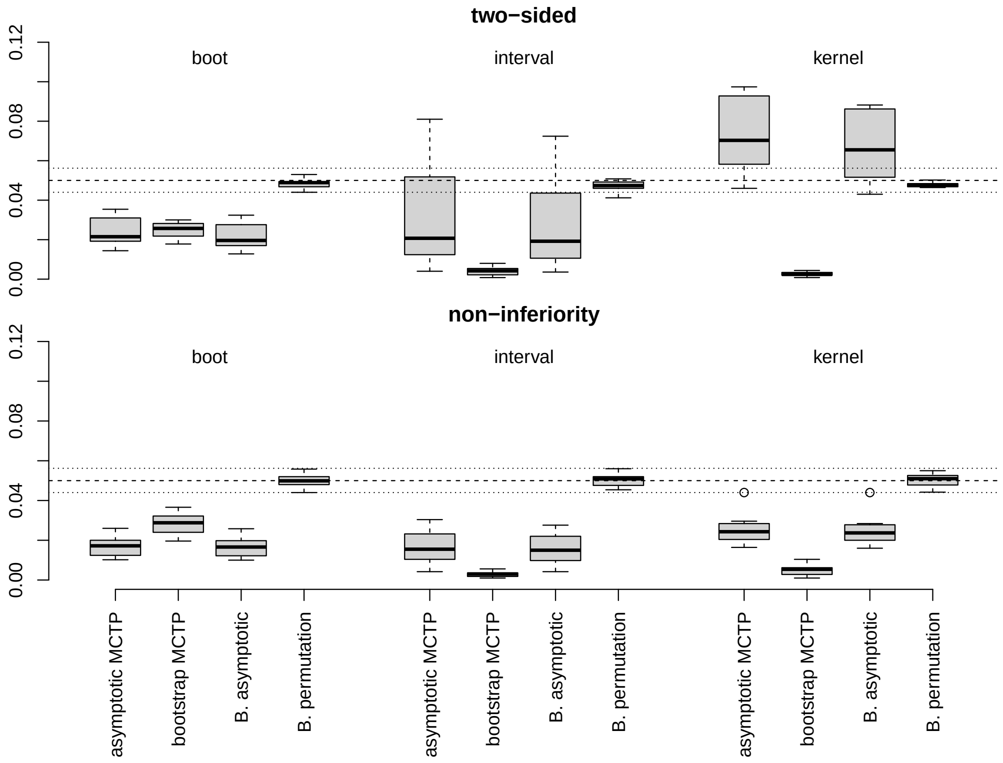

Figure 21: Empirical FWERs for Grand-mean-type contrasts with different hypotheses (top: two-sided and bottom: non-inferiority) and variance estimators (from left to right: bootstrap, interval-based or kernel). The dashed line represents the desired level of significance of $\alpha = 5\%$ and the dotted lines represent the Binomial interval $[0.044, 0.0562]$ for $N_{sim} = 5000$ repetitions.

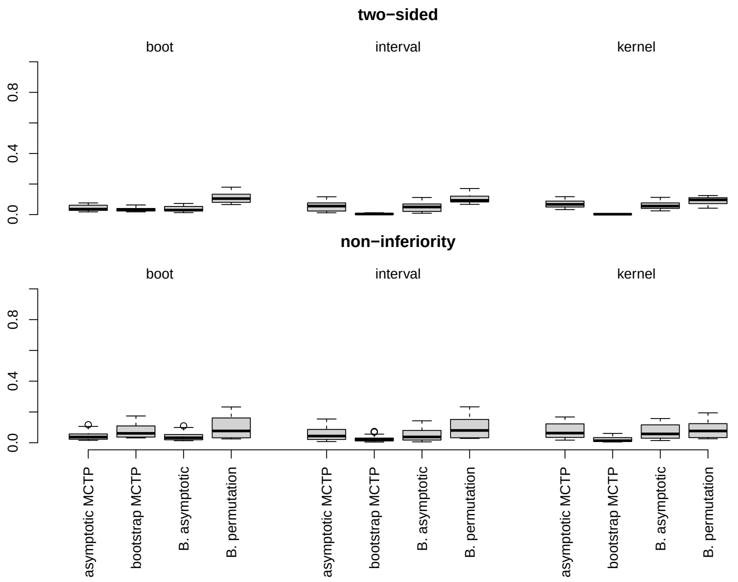

Figure 22: Empirical power with $\delta = 0.0$ for Dunnett-type contrasts with different hypotheses (top: two-sided and bottom: non-inferiority) and variance estimators.

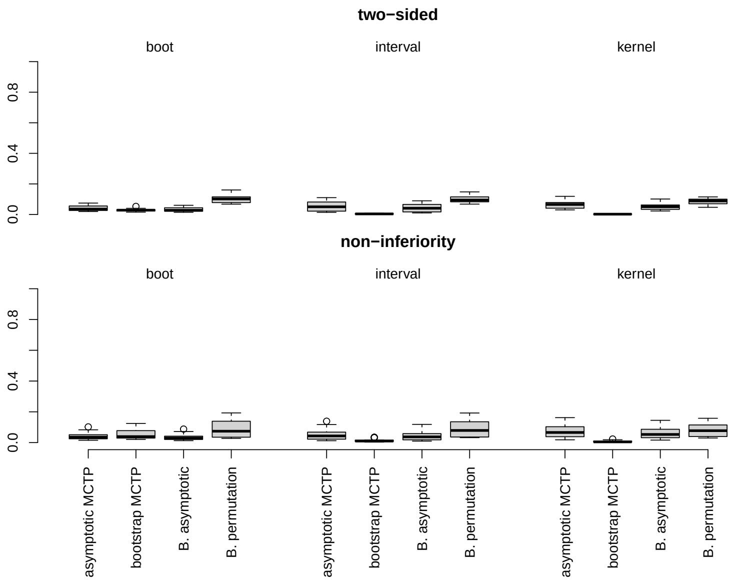

Figure 23: Empirical power with $\delta = 0.0$ for Tukey-type contrasts with different hypotheses (top: two-sided and bottom: non-inferiority) and variance estimators.

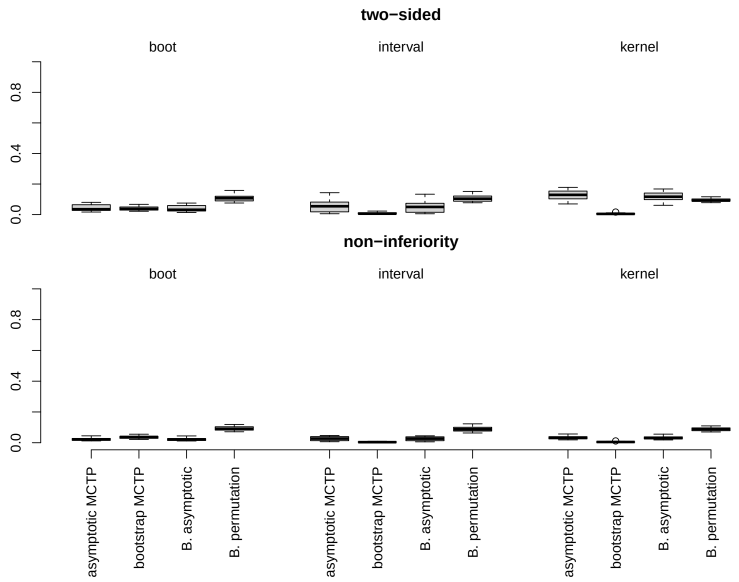

Figure 24: Empirical power with $\delta = 0.0$ for Grand-mean-type contrasts with different hypotheses (top: two-sided and bottom: non-inferiority) and variance estimators.

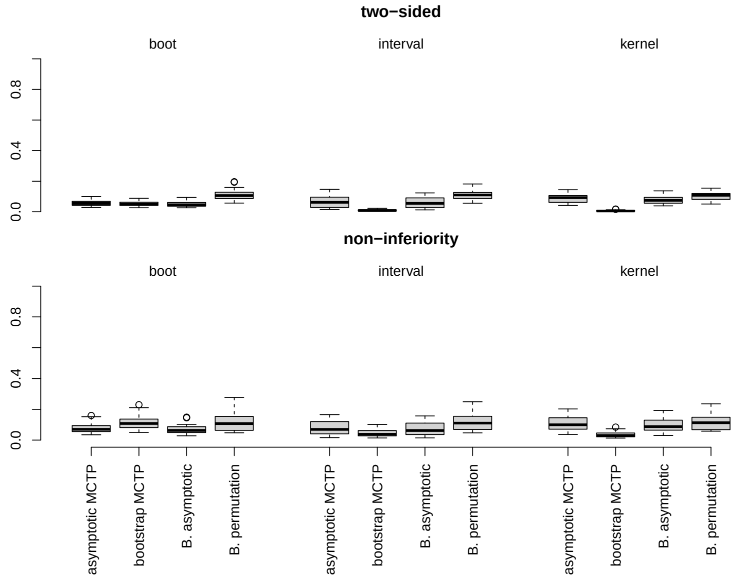

Figure 25: Empirical power with $\delta = 0.5$ for Dunnett-type contrasts with different hypotheses (top: two-sided and bottom: non-inferiority) and variance estimators.

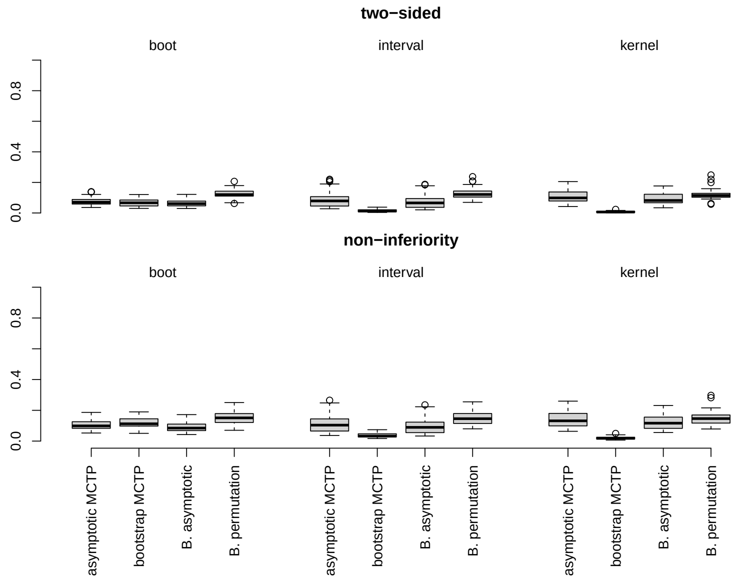

Figure 26: Empirical power with $\delta = 0.5$ for Tukey-type contrasts with different hypotheses (top: two-sided and bottom: non-inferiority) and variance estimators.

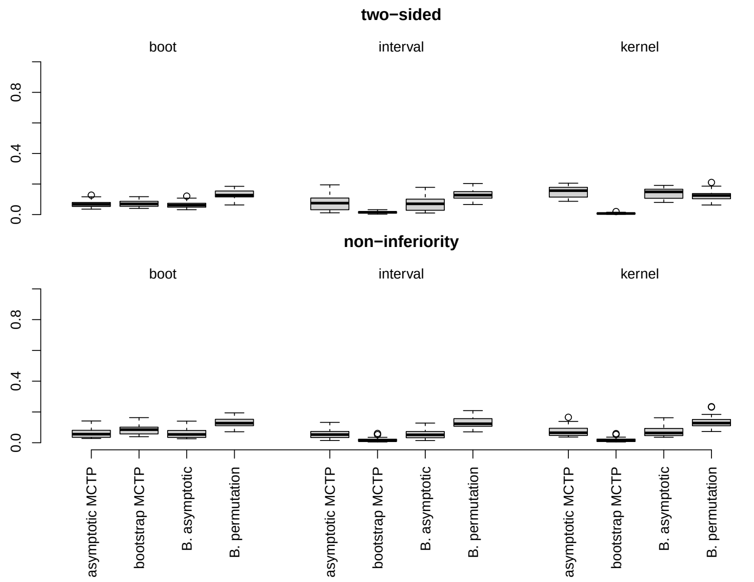

Figure 27: Empirical power with $\delta = 0.5$ for Grand-mean-type contrasts with different hypotheses (top: two-sided and bottom: non-inferiority) and variance estimators.

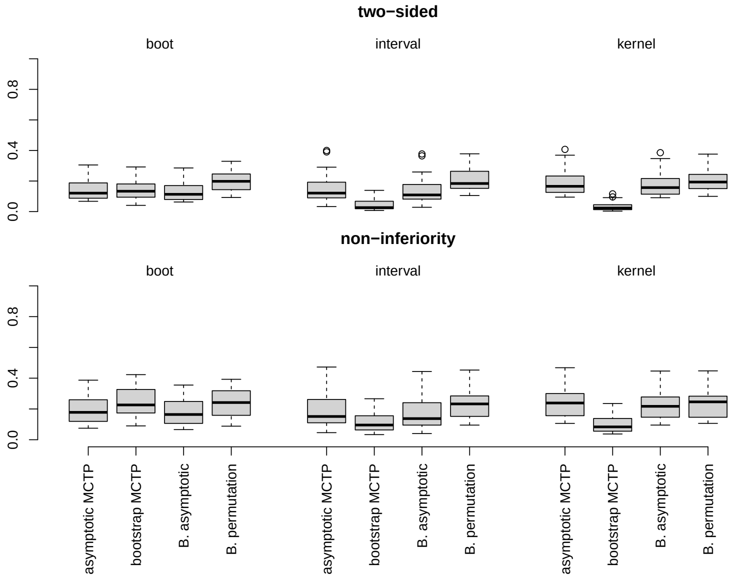

Figure 28: Empirical power with $\delta = 1.0$ for Dunnett-type contrasts with different hypotheses (top: two-sided and bottom: non-inferiority) and variance estimators.

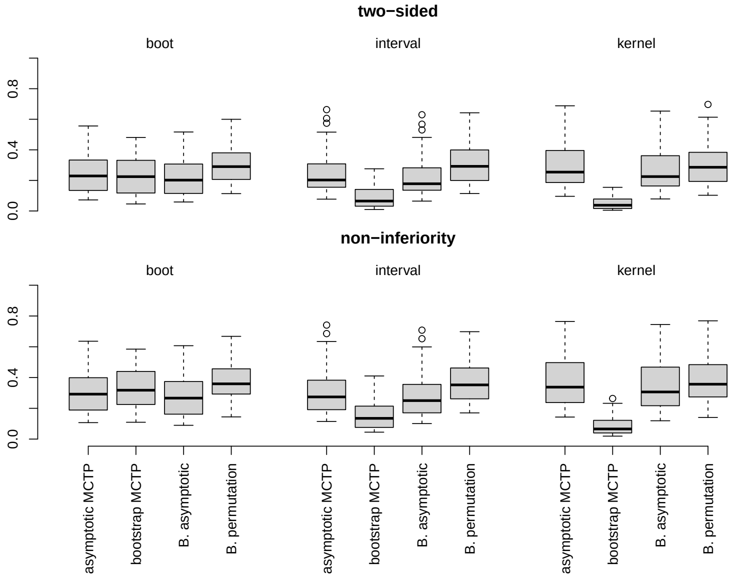

Figure 29: Empirical power with $\delta = 1.0$ for Tukey-type contrasts with different hypotheses (top: two-sided and bottom: non-inferiority) and variance estimators.

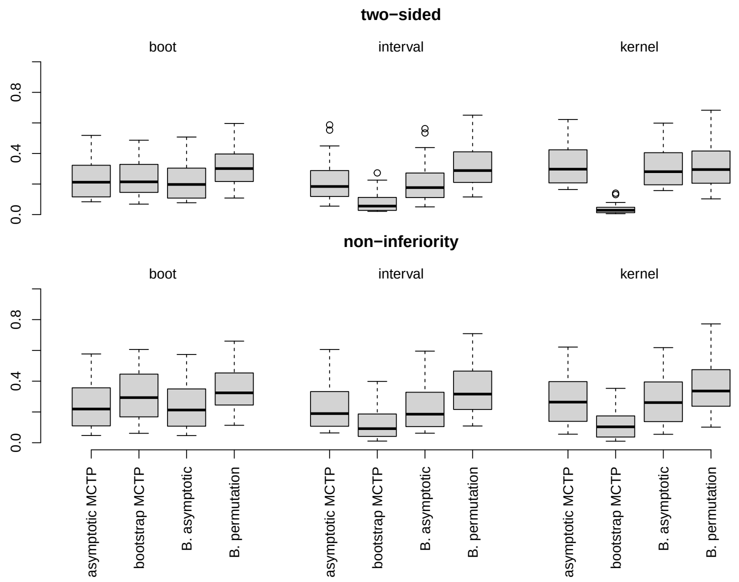

Figure 30: Empirical power with $\delta = 1.0$ for Grand-mean-type contrasts with different hypotheses (top: two-sided and bottom: non-inferiority) and variance estimators.

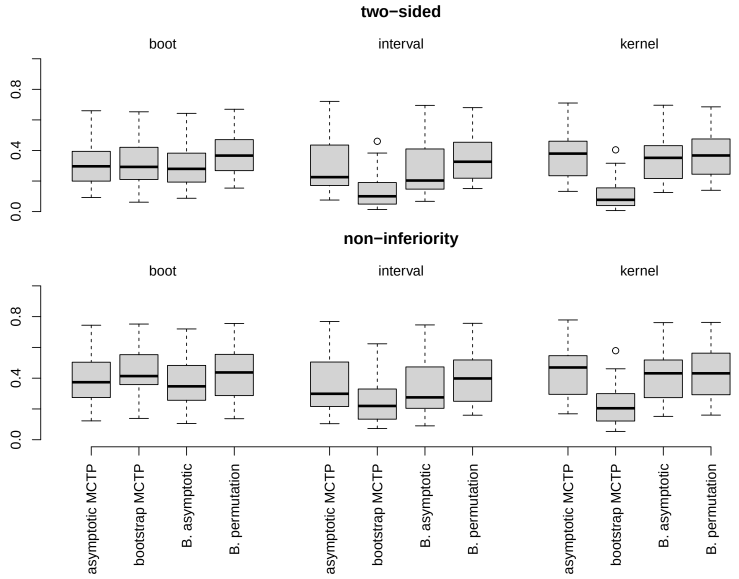

Figure 31: Empirical power with $\delta = 1.5$ for Dunnett-type contrasts with different hypotheses (top: two-sided and bottom: non-inferiority) and variance estimators.

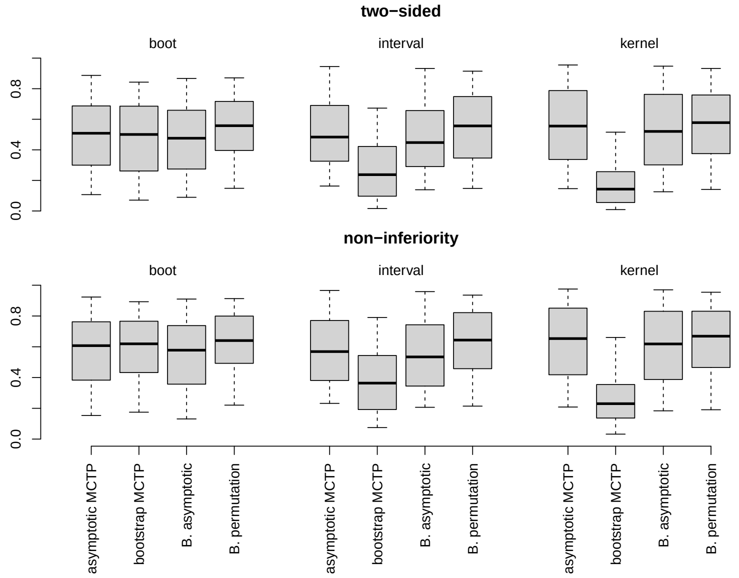

Figure 32: Empirical power with $\delta = 1.5$ for Tukey-type contrasts with different hypotheses (top: two-sided and bottom: non-inferiority) and variance estimators.

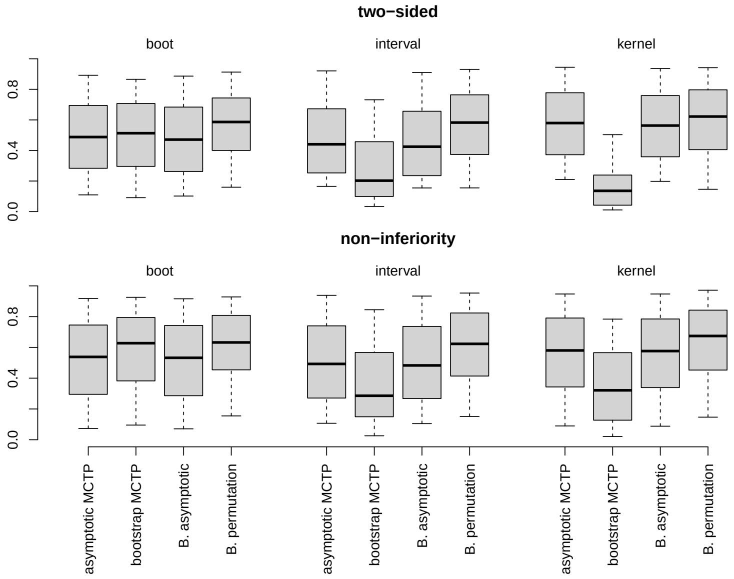

Figure 33: Empirical power with $\delta = 1.5$ for Grand-mean-type contrasts with different hypotheses (top: two-sided and bottom: non-inferiority) and variance estimators.
